# Decoding the regulatory landscape of melanoma reveals TEADS as regulators of the invasive cell state

**DOI:** 10.1038/ncomms7683

**Published:** 2015-04-09

**Authors:** Annelien Verfaillie, Hana Imrichova, Zeynep Kalender Atak, Michael Dewaele, Florian Rambow, Gert Hulselmans, Valerie Christiaens, Dmitry Svetlichnyy, Flavie Luciani, Laura Van den Mooter, Sofie Claerhout, Mark Fiers, Fabrice Journe, Ghanem-Elias Ghanem, Carl Herrmann, Georg Halder, Jean-Christophe Marine, Stein Aerts

**Affiliations:** 1 Laboratory of Computational Biology, Center for Human Genetics, University of Leuven, 3000 Leuven, Belgium; 2 Laboratory for Molecular Cancer Biology, Center for Human Genetics, University of Leuven, 3000 Leuven, Belgium; 3 VIB Center for the Biology of Disease, 3000 Leuven, Belgium; 4 Laboratory of Growth Control and Cancer Research, Center for Human Genetics, University of Leuven, 3000 Leuven, Belgium; 5 Medical Oncology Clinic, Institut Jules Bordet, Université Libre de Bruxelles, 1000 Brussels, Belgium; 6 Department of Theoretical Bioinformatics, DKFZ Heidelberg, 69117 Heidelberg, Germany

## Abstract

Transcriptional reprogramming of proliferative melanoma cells into a phenotypically distinct invasive cell subpopulation is a critical event at the origin of metastatic spreading. Here we generate transcriptome, open chromatin and histone modification maps of melanoma cultures; and integrate this data with existing transcriptome and DNA methylation profiles from tumour biopsies to gain insight into the mechanisms underlying this key reprogramming event. This shows thousands of genomic regulatory regions underlying the proliferative and invasive states, identifying SOX10/MITF and AP-1/TEAD as regulators, respectively. Knockdown of TEADs shows a previously unrecognized role in the invasive gene network and establishes a causative link between these transcription factors, cell invasion and sensitivity to MAPK inhibitors. Using regulatory landscapes and *in silico* analysis, we show that transcriptional reprogramming underlies the distinct cellular states present in melanoma. Furthermore, it reveals an essential role for the TEADs, linking it to clinically relevant mechanisms such as invasion and resistance.

## Abstract (web summary)

The key regulators that allow transition from proliferative to invasive phenotype in melanoma cells have not been identified yet. The authors perform chromatin and transcriptome profiling followed by comprehensive bioinformatics analysis identifying new candidate regulators for two distinct cell states of melanoma.

## Graphical abstract

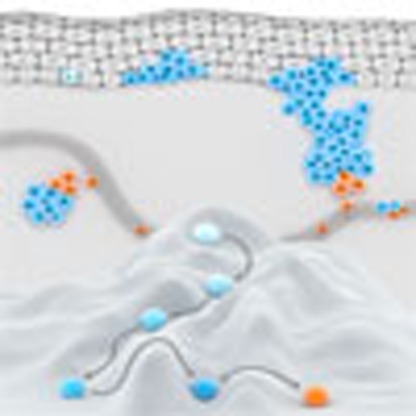

Melanoma is one of the most aggressive cancers and, although investigation into the genetic underpinnings of melanoma have led to promising therapeutics, clinical outcome remains poor, with most patients rapidly acquiring resistance^1^. The difficulty in eradicating melanoma lies in its high degree of heterogeneity and plasticity. Melanoma comprises multiple phenotypically distinct subpopulations of cancer cells, all with a potentially variable sensitivity to therapy^2^. However, the mechanisms evoking this heterogeneity are largely uncharacterized.

Gene expression profiling of cultured melanoma cell lines^3^,^4^,^5^ identified two types of cultures characterized by very distinct transcriptomes. Samples of the ‘proliferative' type express high levels of the melanocyte-lineage-specific transcription factor (TF) MITF^6^ as well as SOX10 and PAX3 (ref. 7, 8). In contrast, samples of the ‘invasive' type express low levels of MITF, high levels of the epithelial-to-mesenchymal transition (EMT)-related TF ZEB1 (ref. 5, 9) and genes involved in TGF-ß signalling. It has been proposed that melanoma invasion is triggered by the appearance of clusters of MITF-low/ZEB1-high cells at the edge of the primary lesions^5^. These cells acquire migratory properties allowing them to invade the dermis, enter the blood stream and eventually contribute to metastatic dissemination. Interestingly, MITF-positive cells are also found at metastatic sites, suggesting an ability of melanoma cells to switch back and forth between these transcriptional states. While several models have been proposed to explain these observations, the initial event always involves a transition in the primary tumour from a proliferative to an invasive cell state. This (reversible) transition is likely caused by dynamic transcriptional changes driven by differential chromatin architecture, and changes in the activity of master regulators and gene regulatory networks^4^,^10^. In support of this, no ‘metastasis-driving' mutations have thus far been found in primary and metastatic tumours from the same patient.

Importantly, it has been proposed that distinct transcriptional cell states characterized by variable MITF or SOX10 activity influence resistance to MAPK pathway inhibitors^1^,^11^. Interestingly, enforcing MITF expression ‘pushes' cells towards a different cell state^12^, which could then be exploited therapeutically. This illustrates how a better understanding of the molecular processes underlying the proliferative-to-invasive transition can be used to overcome drug resistance and improve current therapies. As these processes are largely driven by changes in gene-regulatory networks, new insight may be gained by genome-wide mapping and decoding of the chromatin landscapes and the master regulators that control the distinct transcriptomic states in melanoma.

In this study, we first provide evidence that the cell states described *in vitro* are also recapitulated in microarray and RNA-seq data sets across tumour biopsies. Next, we map the transcriptome and chromatin landscape of 10 short-term melanoma cultures and find thousands of genomic regulatory regions underlying the proliferative and invasive states. Using an integrated approach for motif and track discovery, we confirm SOX10/MITF as master regulators of the proliferative gene network and identify AP-1/TEAD as new master regulators of the invasive gene network. We experimentally validate chromatin interactions upstream of SOX9 by 4C-seq, and we test the TEAD-predicted network using knockdown (KD) experiments. These experiments establish a previously unrecognized role for the TEADs in the invasive gene network and reveal a causative link between these TFs, cell invasion and sensitivity to MAPK inhibitors.

## Results

### Proliferative and invasive gene signatures in tumour samples

**Figure 1:**
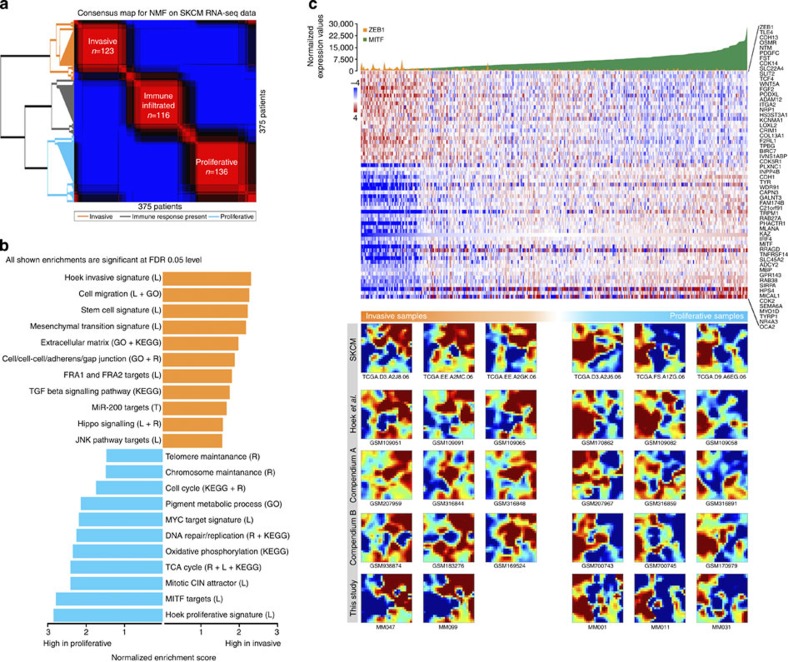
Proliferative and invasive cellular states in melanoma biopsies and cultures. (**a**) Non-negative matrix factorization on TCGA-SKCM RNA-seq data results in three sample clusters. (**b**) Functional characteristics of two states revealed by GSEA on the invasive and the proliferative meta-rankings integrated across SKCM RNA-seq and two microarray compendia, using various sources of functional data (L) Literature; (R) Reactome; (KEGG) KEGG pathways; (GO) Gene Ontology; (T) TargetScan. (**c**) Expression heatmap for TCGA samples showing a core subset of invasive and proliferative gene signatures (the GSEA overlap between the Hoek signatures and our ranking). The samples are ranked according to MITF expression, and the expression levels of both MITF and ZEB1 are indicated on top of the heatmap. Below the heatmap are mosaic plots of several samples. Each mosaic shows the expression of all variable genes in a 25 × 26 grid, whereby each field contains one or more genes. Genes and clusters with similar expression profiles across the cohort are placed close to each other in the grid. The mosaics show a high similarity among the invasive samples, and a strong difference between invasive and proliferative samples. SKCM is RNA-seq data from TCGA; Hoek *et al.*^16^, is microarray data from melanoma cultures; Compendium A and B are melanoma microarray data from GEO (see Supplementary Table 1 for accession numbers used).

The invasive and proliferative transcriptional cell states have thus far only been described *in vitro*. We asked whether the transcriptome of these two distinct cell populations could be observed in clinical samples. To this end, we assembled three compendia of publicly available melanoma gene expression data (Supplementary Table 1). Unsupervised clustering revealed that each of the three compendia clustered into three similar-sized clusters, two exhibiting features reminiscent of the proliferative and invasive cell states and a third exhibiting an immune-related signature presumably due to the presence of a high number of tumour-infiltrating lymphocytes (Fig. 1a,b and Supplementary Figs 1 and 2). Accordingly, the gene signatures derived for both the proliferative and invasive clusters show very significant overlap with the Hoek^3^ gene signatures representing curated gene lists for proliferative and invasive melanoma states in culture (Supplementary Fig. 3a). As expected, samples in the invasive cluster have high expression of genes identified as miR-200 targets or implicated in TGF-ß and JNK signalling, cell migration, stemness and EMT (Fig. 1b). Genes that are upregulated in this cluster include *ZEB1*, *SNAI1* and *TGFB2*. In contrast, genes with high expression in the proliferative sample cluster are significantly enriched for cell cycle, proliferation and melanocytic processes. Genes upregulated in this cluster include known markers of the melanocyte lineage and melanoma such as *SOX10*, *MITF* and *PAX3* (Supplementary Fig. 3b). Consistently, when the entire gene expression pattern of a sample is visualized using self-organizing maps (SOMs)^13^ some of the invasive and proliferative samples show remarkable similarities (Fig. 1c and Supplementary Note 2). In addition, these transcriptomes are highly similar to the transcriptomes of the previously defined invasive and proliferative melanoma cultures. These observations indicate that the clinical samples cluster into distinct groups and that these represent cellular subpopulations in either the proliferative or the invasive cell state. However, whether mutations or transcriptional reprogramming forms the driver of these subpopulations is a matter of debate. Our analysis of the exome re-sequencing data from the same TCGA cohort did not identify specific enrichments for mutations in BRAF, NRAS or any of the other well-established melanoma driver genes (*n*=116) in either of the clusters (Supplementary Note 1). This observation favours the possibility that these very distinct cell states are driven largely by transcriptional reprogramming rather than by common genetic mutations.

Together, these data indicate that, despite the fact that all of these samples originate from different human patients with very divergent mutational profiles, most tumours fall into one of these two dominant states. Note that when analysing the immune-infiltrated cluster separately, the samples also show a tendency towards either of these two states, suggesting that the immune signature may, at least partly, represent a layer that confounds the underlying melanoma states (Supplementary Fig. 4).

### Changes in the chromatin landscape underlie cellular states

**Figure 2:**
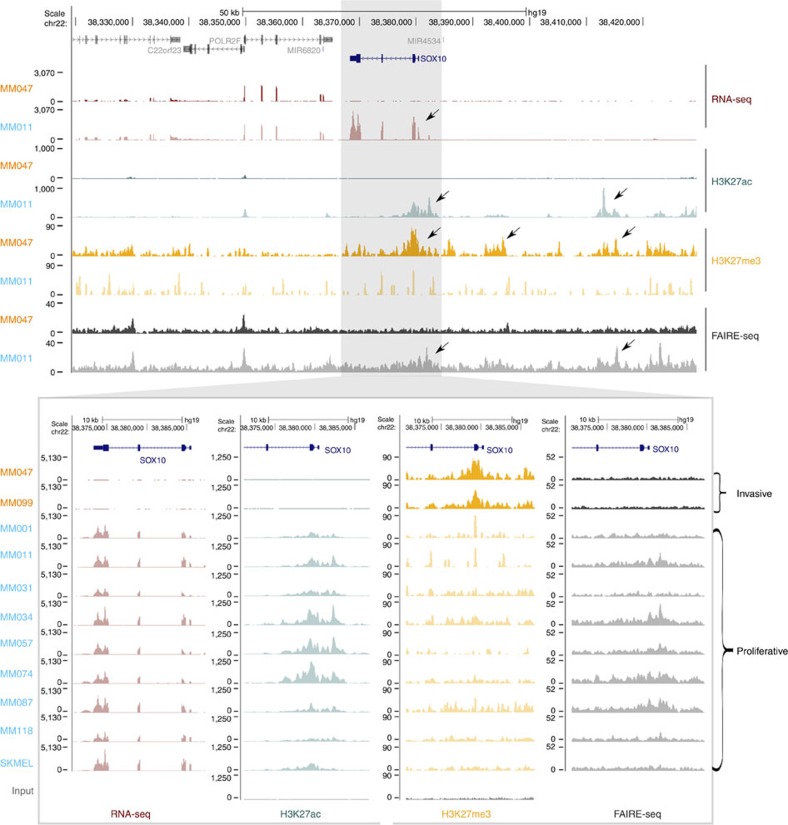
Transcriptome and epigenome profiling in 11 melanoma cell cultures. RNA-seq, FAIRE-seq and ChIP-seq against H3K27Ac and H3K27me3 across 10 short-passage melanoma cultures and one melanoma cell line SK-MEL-5. The *SOX10* gene shows high expression and its upstream regions contain high H3K27ac and FAIRE but low H3K27me3 signal in the nine proliferative (blue) samples. In the two invasive (orange) samples, there is no *SOX10* expression, no H3K27Ac and FAIRE peaks but high H3K27me3 peaks. Upper panel shows one invasive sample (MM047) and one proliferative sample (MM011). Lower panels showing zoom in around the promoter region of *SOX10* with tracks for all 11 samples for each of the four data types. Vertical axes represent normalized coverage for each data track. Arrows indicate regions of interest that are different between proliferative and invasive states. Other genes are illustrated in Supplementary Figs 4 and 5 and in the UCSC Genome Browser using our Melanoma Track Hub (see Methods).

The above findings support *in vitro* short-term cultures as a valid model system that can be exploited to decipher the chromatin landscapes and regulatory networks underlying these two transcriptional cell states. Therefore, we profiled the transcriptome and chromatin landscape of 10 short-passage melanoma cultures previously described^14^ and one classical melanoma cell line (SK-MEL-5; see accession code for data availability^15^). The transcriptome of all 11 samples was compared with publicly available gene expression data from melanoma cultures and with the clusters of tumour biopsies described above using SOMs (Fig. 1c). This comparison indicated that cultures MM047 and MM099 are in an invasive transcriptional state, while the remaining harbour a transcriptome reminiscent of the proliferative state (Supplementary Table 2). This correspondence was further supported by a significant enrichment of the invasive and proliferative gene signatures from Hoek *et al.*^3^, and by the high expression of invasive marker genes such as *ZEB1*, *SOX9* and *WNT5A* (Supplementary Figs 3, 5 and6). In contrast, these samples have undetectable *SOX10* and *MITF*, while the other nine samples express high *SOX10* and *MITF* levels (Fig. 2, Supplementary Figs 3a and 7). Using these data we established a new gene signature for each state, consisting of 772 and 643 genes for the proliferative and invasive phenotypes, respectively (Supplementary Data 1). Comparing our data with the Hoek gene signatures^3^,^16^ identified 100% of Hoek's proliferative genes upregulated in our proliferative samples, while 100% of Hoek's invasive genes are upregulated in our invasive samples (Supplementary Fig. 3a). Importantly, the cells with an invasive transcriptional profile do exhibit enhanced capabilities to invade in a Matrigel assay compared with the cell lines with a transcriptional proliferative state (Supplementary Fig. 8). In addition, similar to the results obtained using the TCGA cohort, the proliferative versus invasive split is not correlated with any specific mutations in known melanoma driver genes, such as *BRAF* (Supplementary Fig. 9). Again, this is consistent with the view that acquisition of the invasive cell state is likely to be a consequence of transcriptional reprogramming rather than being driven by any specific genetic alterations.

**Figure 3:**
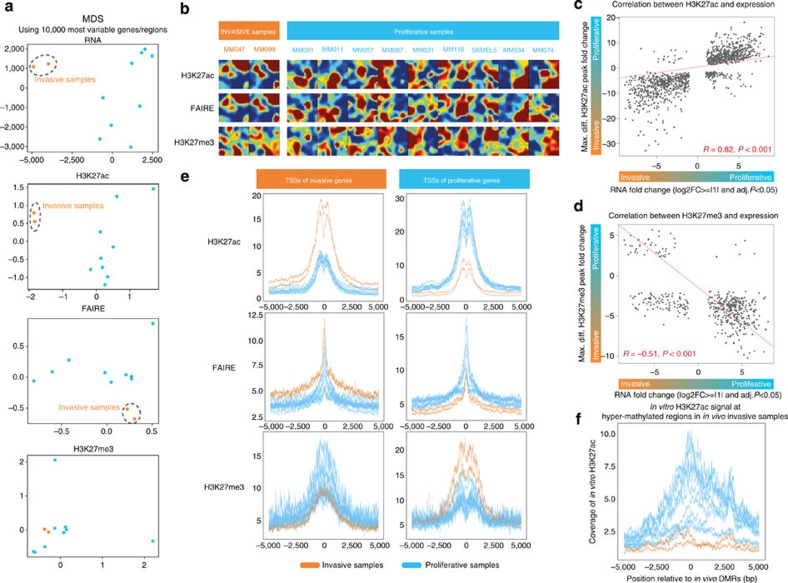
Global changes in the chromatin landscape between proliferative and invasive states. (**a**) Multidimensional scaling using RNA-seq, H3K27ac and FAIRE-seq data reveals a clear separation of the invasive samples, MM047 and MM099, from the samples in the proliferative state. (**b**) Mosaic plots obtained by clustering 55,919 regulatory regions show very similar chromatin profiles for MM047 and MM099, while the proliferative samples are characterized by higher heterogeneity. (**c**) Gene expression changes between invasive and proliferative samples correlate with changes in H3K27ac (for each gene from our signatures, the H3K27Ac differential peak called by MACS2 with the largest fold change in 20 kb around the TSS is selected). Spearman's rank correlation of coefficient is shown. (**d**) Inverse correlation between H3K27me3 peaks and gene expression changes. (**e**) Aggregation plots of the read coverage (*y* axis) indicate that the TSS (*x* axis) of genes that are expressed higher in invasive samples (643 genes, left column) show higher FAIRE and H3K27ac signal but lower H3K27me3 signal in the same invasive (orange) samples than in the proliferative samples. *Vice versa*, the TSS of genes that are more expressed in the proliferative samples (772 genes, right column) show higher activating signals in the proliferative samples, and higher repressive signal in the two invasive samples. (**f**) Concordance between chromatin landscape *in vitro* and *in vivo*, where *in vivo* hypermethylated regions in the invasive samples from TCGA data show high activity (H3K27ac) in proliferative *in vitro* cultures (nine blue curves), but no activity in the invasive cultures (two orange curves).

The two expression profiles we identified likely arise through gene regulation by *cis*-regulatory modules. Therefore, we next investigated active *cis*-regulatory regions underlying the invasive and proliferative transcriptional states using open chromatin profiling (FAIRE-seq^17^) and ChIP-seq against two important histone modifications representing activated (H3K27ac) and repressed (H3K27me3) chromatin marks. Interestingly, the invasive samples are clustered separately on the basis of their chromatin activity profiles, similarly to the clustering on the basis of RNA-seq data. For example, the H3K27ac and FAIRE-seq tracks indicate an active and open *SOX10* promoter, respectively, in the nine proliferative samples with high *SOX10* expression. In contrast, the *SOX10* promoter lacks activating marks, but carries repressing H3K27me3 marks in the two invasive cultures (Fig. 2). This reciprocity is a genome-wide property since the two invasive samples clearly segregate from the other samples in a multidimensional scaling unsupervised analysis of the H3K27ac or FAIRE-seq peaks (but not H3K27me3; Fig. 3a). An overview of the entire chromatin landscape, using SOMs based on 55,919 genomic enhancers and promoters, indicates that the difference between the two cellular states is widespread and involves thousands of regulatory regions (Fig. 3b and Supplementary Note 2). Using two complementary computational approaches we predicted 13,453 and 6,669 regions to have a regulatory role in the invasive and proliferative states, respectively (Supplementary Fig. 10, see Methods). Interestingly, when comparing enhancers active in the proliferative transcriptional state to 110 different sets of tissue-specific enhancers identified by the expression of enhancer-RNA^18^, melanocyte is identified as the cell type with the highest overlap of enhancers. In contrast, the invasive melanoma enhancers overlap most strongly with enhancers specifically active in skin fibroblasts, which are known to harbour a mesenchymal regulatory programme (Supplementary Table 3).

Next, we investigated whether there is a global correlation between active chromatin and gene expression. Although the assignment of active promoters and enhancers to candidate target genes is not a trivial task, an analysis of (differential) H3K27ac, FAIRE and H3K27me3 peaks around the transcription start site (TSS) of their nearest differentially expressed genes (<20 kb around TSS) indicates a strong correlation between both layers (Fig. 3c,d and Supplementary Fig. 11). Particularly, the TSS of invasive genes shows strong regulatory activity in the invasive samples, but low activity in the proliferative samples, while the repressive H3K27me3 mark shows the opposite (Fig. 3e and Supplementary Fig. 12). These data confirm that these specific chromatin marks are robust predictors of the transcriptional activity in a specific cellular state.

We then asked whether the invasive and proliferative chromatin landscapes are also reflected in *in vivo* tumour biopsies. Hence, we used both the RNA-seq and DNA methylation data from TCGA. The TSSs of the differentially expressed genes between invasive and proliferative sample clusters are correlated with active chromatin in the corresponding in-house samples (Supplementary Fig. 13). Remarkably, differentially methylated regions between the invasive and proliferative clinical samples are correlated as well with both gene expression and active and repressed chromatin in the melanoma in-house samples (Supplementary Figs 14 and 15). Particularly, regions that are significantly hypomethylated in the proliferative group (that is, likely active in the tumour) are also activated (H3K27ac and FAIRE) in the proliferative cultures, but not in the invasive cultures (Fig. 3f). Likewise, enhancers that are only active in the invasive cultures are significantly hypomethylated in the invasive clinical samples (Supplementary Fig. 16). These findings show that the regulatory landscape can reflect transcriptional programme changes, thus forming an interesting basis to search for causal TFs driving these distinct regulatory programmes.

### Sequence analysis and ChIP reveal regulators for each state

**Figure 4:**
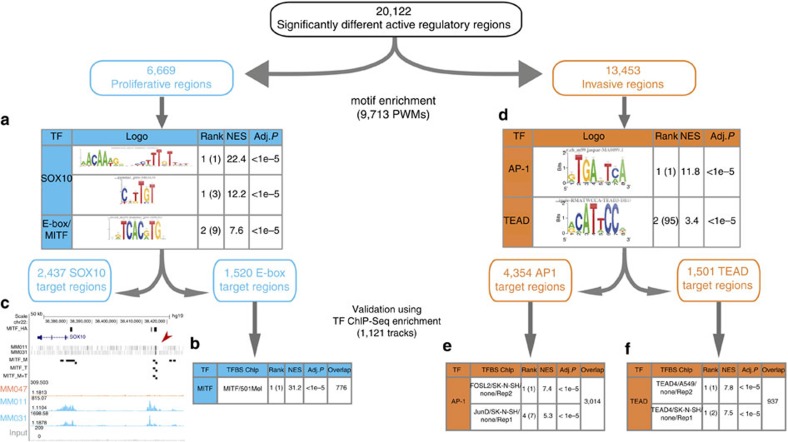
Enhancer signatures are enriched for transcription factor motifs and ChIP-seq tracks. (**a**) The proliferative enhancer signature (6,669 regions) is most strongly enriched for SOX10 motifs (a SOX dimer motif is most significant), and E-box motifs (the second best scoring motif cluster, where the most significant E-box motif is ranked ninth after eight SOX10-like motifs). (**b**) The E-box-predicted enhancers are correlated with publicly available MITF ChIP-seq data (against HA-tagged MITF) in a proliferative melanoma culture. (**c**) The same public ChIP-seq data (MITF_HA) and in-house ChIP-seq data against endogenous MITF in two proliferative cultures (MM011 and MM031) confirm that *SOX10* is a MITF target gene through the predicted upstream enhancer (arrowhead). MITF predicted binding sites (MITF_M) inside H3K27Ac peaks (blue peaks in proliferative samples) ∼30 kb upstream of SOX10 MITF overlap with MITF_HA and MITF ChIP-seq peaks. (**d**) The invasive enhancer signature (13,453 regions) is most strongly enriched for AP-1 motifs (best scoring motif cluster) and TEAD motifs (the second best scoring motif cluster; Supplementary Fig. 15). (**e**) The predicted AP-1 enhancers are tested against all ENCODE ChIP-seq data and are correlated most strongly with ChIP-seq peaks of the AP-1 complex members such as FOSL2 and JUND, derived from a neuroblastoma cell line (SK-N-SH; ranked first out of 1,121 tested ChIP-seq data sets). (**f**) Likewise, TEAD-predicted target enhancers are most strongly correlated with TEAD4 ChIP-seq in a neuroblastoma (SK-N-SH) and lung cancer (A549) cell line.

Having identified differentially active regulatory regions and differentially expressed genes between the invasive and proliferative states, we searched for TFs contributing to the chromatin and transcriptional cell state distinction. We employed a large collection of public data sets to search for TFs, of which the DNA-binding motif and/or ChIP-seq tracks significantly overlap with active regulatory regions (see Methods). Within the 6,669 regulatory regions activated in the proliferative cell state, the SOX10 motif is the most significantly enriched and is predicted to target 2,437 of these regions (36.5%; Fig. 4a and Supplementary Fig. 17a). Moreover, one of its highest scoring target enhancers (position 292 out of 1,223,024 regions in the genome) is located immediately upstream of the *SOX10* gene itself, confirming earlier observations that SOX10 is autoregulatory^19^. Another SOX10 candidate target region (ranked 4,089) is located upstream of the *MITF* locus, confirming the possibility that SOX10 directly regulates *MITF*^20^.

The second most enriched motif is an E-box motif with 1,520 predicted target regions (22.8%). Since MITF binds DNA through E-boxes, we assessed whether these enhancers represent *bona fide* MITF target sites. The first ranked ChIP-seq track found is the one against an overexpressed haemagglutinin (HA)-tagged MITF^21^, indicating a highly significant correlation between the E-box/MITF predictions and the ChIP-seq data (hypergeometric adjusted *P*-value<1.0e−05, Normalized Enrichment Score=31.2). This analysis enabled us to define an optimal set of direct MITF targets having both the motif and a ChIP-seq peak, and contains 776 regulatory regions (Fig. 4b and Supplementary Fig. 17). Interestingly, one of the predicted MITF-binding sites is located upstream of the *SOX10* gene, indicating the existence of a direct cross-activation between MITF and SOX10. In addition, we performed a ChIP-seq experiment against endogenous MITF in two of the proliferative cell lines (MM011 and MM031), confirming that MITF ChIP peaks are enriched among the predicted E-box enhancers, and that the *SOX10* gene is likely a direct target of MITF (Fig. 4c and Supplementary Fig. 17).

The above results confirm that our approach for inferring master regulators using the regulatory landscape is valid as it identified the known master regulators of the proliferative state. Therefore, we repeated this analysis for the 13,453 regulatory regions activated in the invasive cell state, since no pronounced master regulators have been postulated for this state. Interestingly, the AP-1 motif is most significantly enriched, being present in 4,354 (32%) active regulatory regions (Fig. 4d and Supplementary Fig. 18). To validate these predictions we performed track discovery and found as first ranked track the ChIP-seq of FOSL2, an AP-1 family member, obtained from the neuroblastoma SK-N-SH cell line, strongly indicating that the predicted regions are *bona fide* AP-1-binding sites (Fig. 4e). Even more intriguing is the detection of the DNA-binding motifs of the TEAD factors, known effectors of the Hippo signalling pathway^22^, as the second most enriched motif cluster in the invasive cell state (Supplementary Fig. 18). Eleven variants of the TEAD motifs are enriched, together yielding 1,501 (11%) predicted TEAD target regions transcriptionally active in the invasive state (Fig. 4d and Supplementary Fig. 18). Importantly, we confirmed that these regulatory regions are likely direct TEAD targets as they are strongly enriched for TEAD ChIP peaks; in particular, TEAD4 ChIP-seq tracks from SK-N-SH and A549 cell lines ranked first (Fig. 4f).

In conclusion, these analyses allowed the identification of TFs that regulate a large number of promoters and enhancers, thereby shedding light on how transcriptional reprogramming can distinguish two distinct melanoma cell states. Most intriguing is the fact that our data allow us to concretely put forward two putative candidate master regulators for the invasive state.

### Mapping and validating the proliferative and invasive networks

**Figure 5:**
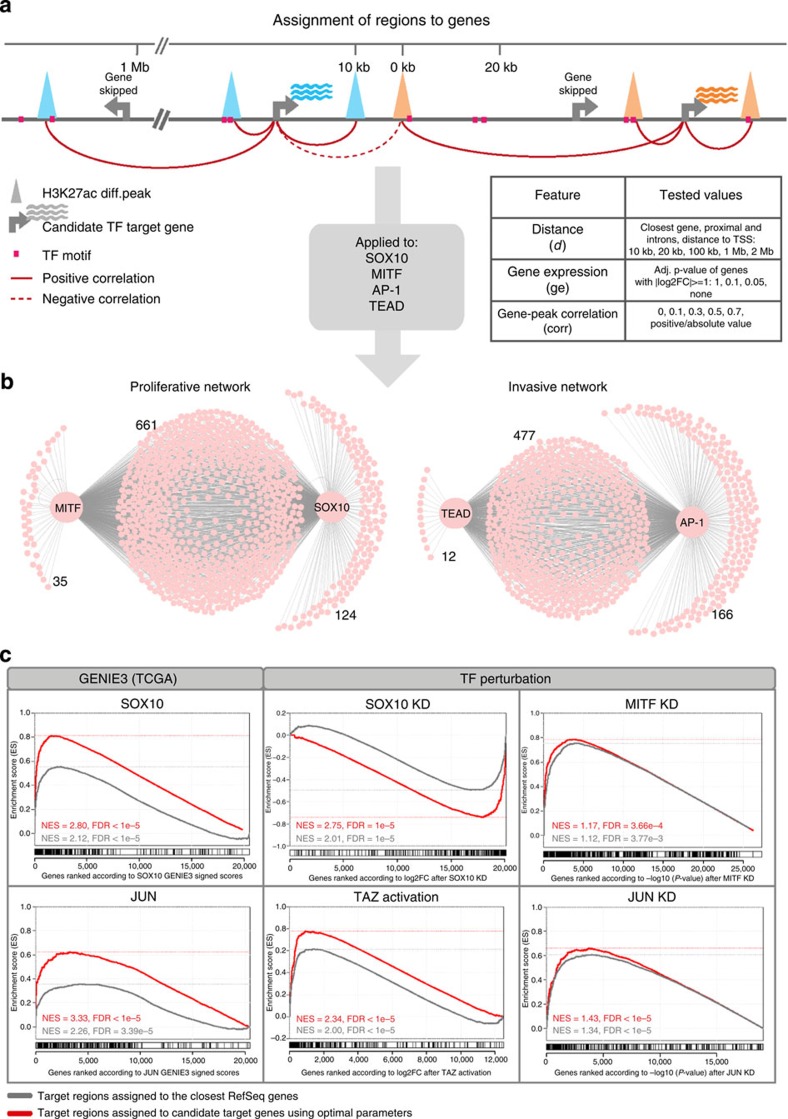
Mapping gene regulatory networks from the enhancer signatures. (**a**) Cartoon showing the assignment of regions to genes. Each enhancer from either the invasive or proliferative enhancer signature is associated (red arches) with zero, one or more candidate target genes using various parameter settings, allowing very distal interactions up to 10 kb, 100 kb, 1 Mb, or 2 Mb from the TSS, with or without filtering for target genes having corresponding gene expression data. (**b**) Predicted invasive (right) and proliferative (left) networks showing high overlap between AP-1 and TEAD targets in the invasive network, and high overlap between MITF and SOX10 targets in the proliferative network. Region-to-gene association parameters used for this network are (*d*=1 Mb; ge=0.1; corr=0.3; see Methods). (**c**) Network validation using GSEA showing that predicted target genes for SOX10, AP-1, TEAD and MITF are functional targets based on co-expression (GENIE3-based co-expression network on TCGA RNA-seq) and publicly available perturbation data for each factor (see Methods). All shown enrichments are significant with FDR<0.0001 (except results for MITF KD where the shown enrichments are significant with FDR=0.0004 and FDR=0.0038). Target genes predicted by distal assignments (red curves) have more accurate predictions than assignments based on the closest genes (grey curves). Optimal region-to-gene association parameters used for the gene sets represented by red curves are: SOX10 (*d*=100 kb and closest, ge=0.05, corr=0.1), MITF (*d*=2 Mb, ge=0.05, corr=0.3), AP-1 (*d*=20 kb, ge=1, corr=0.1), TEAD (*d*=100 kb and closest, ge=1, corr=0.1 and absolute value).

Deciphering functional gene regulatory networks underlying cellular states requires an integration, or ‘projection' of the *cis*-regulatory landscape on the differentially expressed genes of each state to identify potential target genes of the enhancers. By connecting the master regulators to functional target genes, via the active regulatory regions, a functional network can be inferred. Consequently, we predicted genome-wide enhancer-target gene interactions for MITF, SOX10, TEAD and AP-1. Most studies limit enhancer-to-gene mappings to a relatively small intergenic space around each gene. By limiting this analysis to 20 kb around the TSS, only 1,404 (7%) enhancers can be assigned to genes. This is because enhancer–promoter interactions can occur over distances of upto a few mega base pairs by looping^23^. To associate distal enhancers to their candidate target genes, we tested various parameter settings (Fig. 5a, see Methods) linking 4,599 enhancers (23%) to 1,477 target genes and thus yielding a large gene regulatory network for each state (Fig. 5b and Supplementary Data 2). To assess the quality of the target gene predictions, and to compare different assignment procedures, we used two rounds of validation with public data. In the primary validation round, we tested whether the assigned target genes are overall *co-expressed* with their respective regulator across the TCGA data set, whereby co-expression values were calculated using GENIE3 (ref. 24; Fig. 5c and Supplementary Fig. 19). Particularly, this network inference method yielded a ranked list of downstream targets of SOX10 and JUND on the basis of (linear and nonlinear) co-expression in the TCGA data. As shown in Fig. 5c, the predicted direct targets of SOX10 and AP-1 in the network are significantly enriched in the top of this co-expression ranking (False Discovery Rate <1E−5 by gene set enrichment analysis (GSEA)). Therefore, this analysis demonstrates the usefulness of including distal assignments in addition to the proximal assignments, and that assignments to the closest correlated gene are better than assignments to the closest gene without using any information on the expression of that gene.

**Figure 6:**
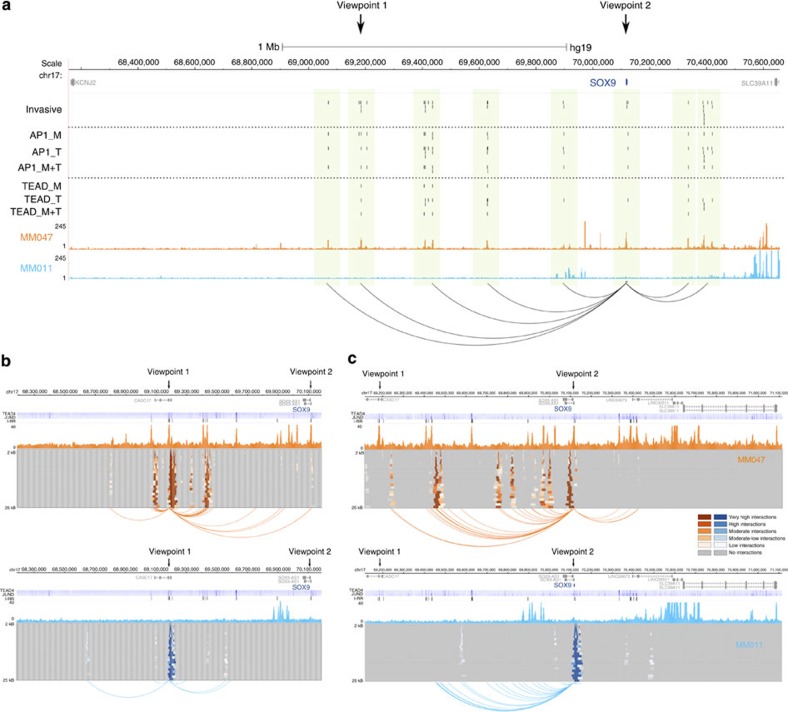
Long-range enhancer–promoter interactions at the SOX9 locus. (**a**) View of a 2-Mb region around *SOX9* showing eight clusters of predicted AP-1 and TEAD enhancers that are specifically active in the invasive state, as reflected by the H3K27ac peaks present for MM047 (orange) and absent in MM011 (blue). Different tracks show the motifs (AP1_M, TEAD_M) or ChIP-seq tracks (AP1_T, TEAD_T) detected for AP-1 and TEAD within these clusters. Arcs indicate correlations between the H3K27ac profile (vector of 11 peak values) of distal enhancers and the expression profile (vector of 11 expression values) of *SOX9*. Viewpoints 1 and 2 indicate the selected anchor points for 4C interaction analysis. (**b**) 4C-seq performed in an invasive (MM047) and a proliferative culture (MM011) showing chromatin interactions with viewpoint 1, a region 1 Mb upstream of *SOX9* TSS. This enhancer interacts with other distal enhancers and with the *SOX9* promoter, only in the invasive sample. I-RR represents invasive regulatory regions, while TEAD4 and JUND represent tracks from publicly available ENCODE ChIP-seq data on the SKSH cell line. The domainograms show the identified interactions after a window-based analysis (from 2 to 25 kb). Colour gradients represent the interaction signal strength, *x* axis represents the analysis window sizes and the arcs below represent significant interactions at *P*-value<0.05 threshold. (**c**) 4C-seq performed in an invasive (MM047) and a proliferative culture (MM011) showing chromatin interactions with viewpoint 2, the promoter of *SOX9*. The *SOX9* promoter interacts mostly with upstream enhancers.

In a secondary validation round, we used publicly available genetic perturbation data for each of the predicted master regulators to examine whether the predicted targets are functionally dependent or downstream of their regulator^8^,^21^,^25^,^26^. For each of the four TFs, we found a significant overlap between the target gene predictions and the genes expressed downstream of the corresponding TF after perturbation, again using ranked gene lists and GSEA analysis (Fig. 5c). These results validate our predictions and indicate that these targets are likely to be functionally important. Interestingly, the invasive network shows a very high and significant overlap between the TEAD and AP-1 target genes (Fig. 5b), indicating that the regulatory function of TEAD is strongly related to AP-1 function in this particular context. This finding is corroborated by a high degree of overlap (*r*=0.38 with *P*-value<2.2e−16) between JUND and TEAD4 ChIP-seq peaks in the SK-N-SH neuroblastoma cell line (Fig. 6a). Together, these data indicate that TEAD may bind cooperatively with AP-1 to regulate its target genes.

In conclusion, *in silico* exploitation of epigenomic data allowed us to infer master regulators for each melanoma cellular state and identify many downstream targets, without discarding distal regulatory regions. The networks thus created are likely to represent an important part of the global gene regulatory network underlying melanoma cell state distinction.

### 4C-seq shows long-range interactions at the SOX9 locus

The inferred gene regulatory networks are on the basis of predictions of long-range enhancer–promoter interactions to associate distal enhancers to candidate target genes. These predictions are derived from correlations between enhancer activity profiles and gene expression profiles. To test whether such associations indeed reflect true three-dimensional (3D) chromatin interactions, we focused on one particularly relevant target gene of the invasive network, *SOX9* (Fig. 6) and performed Circularized Chromosome Conformation Capture sequencing (4C-seq)^27^. SOX9 is a TF specifically expressed in the invasive state and is mostly known for its involvement in early neural crest development^28^. In addition, SOX9 has previously been implicated in invasive growth in other cancer types such as prostate cancer^29^. The *SOX9* locus contains a very large intergenic region of ∼2 Mb upstream of the promoter region. We identified 31 significantly active regulatory regions—clustered into eight subregions—within 1.4 Mb around *SOX9* (Fig. 6a). Each of these distal enhancers is positively correlated with both the activity of the *SOX9* promoter and its expression levels across the 11 melanoma in-house samples indicating that the distal elements may indeed interact with the *SOX9* promoter and regulate *SOX9* transcription. To further test this we performed 4C-seq on both MM047 and MM011 using the promoter as well as a 1-Mb upstream enhancer as viewpoints (Fig. 6b,c). A large number of interactions can be observed in the SOX9-positive and invasive MM047, but not in the SOX9-negative MM011. Interestingly, 35/44 (79.5%) of all interactions identified are found upstream, showing a strong bias towards the upstream region. Remarkably, no specific interactions between distal enhancers and the promoter can be observed in MM011, a strong indicator that these interactions can drive *SOX9* activation. Thus, these results indicate that multiple distal enhancers can interact with a single promoter, and that (long-range) chromatin interactions can differ between melanoma cellular states. This also shows that correlations between enhancers and genes can be used to predict enhancer–promoter interactions, as previously shown for DNAseI hypersensitivity sites and chromatin marks^30^,^31^.

### TEADs are the key regulators of the invasive state and phenotype

**Figure 7:**
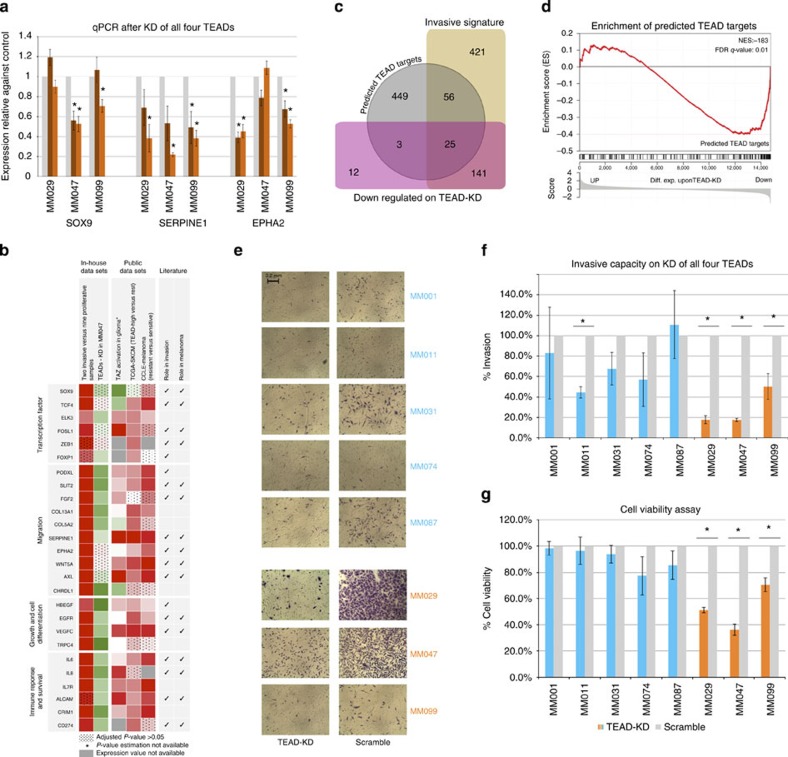
TEAD as a master regulator for the invasive phenotype. (**a**) Simultaneous knockdown of all four TEADs causes downregulation of *SOX9, SERPINE1* and *EPHA2* expression in the invasive cultures as measured using qPCR. Measurements were normalized against the non-target control within each culture and are averaged across at least three biological replicates. Error bars represent s.e.m., (asterisk=*P-*value<0.05). *P*-values were determined using Student's *t*-test. Dark orange bars=72 h after transfection; lighter orange bars=96 h after Transfection. (**b**) Selection of genes highly expressed in the invasive state and downregulated on TEAD knockdown categorized into several functional groups relevant to the invasive phenotype (see Supplementary Data 3 for the entire list of annotated TEAD targets). In addition, expression information of TCGA and CCLE data for each gene is provided. (**c**) Significant overlap of genes predicted as TEAD targets (grey) with genes assigned to the invasive signature (yellow; hypergeometric *P*-value=5.83E−11) or with genes downregulated on TEAD knockdown (pink; hypergeometric *P*-value=1.37E−23). (**d**) GSEA with genes ranked according to their differential expression on TEAD knockdown show a strong enrichment of predicted TEAD targets among the downregulated genes. (**e**) Images showing the reduced invasive capacity of MM029, MM047 and MM099 on knockdown of the TEADs (all images were made at magnification × 20, scale bar, 0.2 mm). (**f**) Knockdown of all four TEADs using a siRNA pool leads to a significant (*P*-value<0.05) reduction of the invasive capacity compared with a non-target control siRNA for all three invasive cultures. Results are averaged across at least three biological replicates. (**g**) Cell viability on knockdown of all four TEADs decreases significantly. *P*-values were determined using Student's *t*-test and the error bars represent s.e.m.

The four TEADs in the human genome have all been implicated as the key effectors of the Hippo pathway, a pathway previously shown to confer invasive properties to various cancers including breast^32^, oesophageal^33^ and more recently melanoma^34^. Note, however, that the implication of the Hippo pathway in melanoma was linked to the Hippo transducers YAP and TAZ, which exhibit multiple TEAD-independent functions among which modifying TGF-ß and WNT signalling^35^,^36^. In contrast, by using *in silico* tools and public data sets we raise the possibility that the TEADs are directly involved—as master regulators—in the invasive melanoma cell state. For example, we find multiple invasive-specific H3K27ac peaks with predicted TEAD motifs that overlap with TEAD ChIP-seq data from ENCODE, in the neighbourhood of invasive genes (Supplementary Fig. 20). To test whether these predictions indeed point to functional targets we KD all TEADs simultaneously and asked whether our predicted TEAD target genes are affected by this perturbation. The transcriptome of MM047 on TEADs' KD was established by RNA-seq and showed significantly decreased levels of all *TEADs* (3.8- to 7.1-fold; Supplementary Fig. 21). Importantly, a significant subset of all predicted TEAD target genes were downregulated including *SOX9*, *SERPINE*, *EHPA2* and several Hippo pathway genes (Fig. 7a,b,d and Supplementary Fig. 22). Interestingly, many of the TEAD-regulated genes have already been linked to cell migration, invasion or metastasis, where their involvement in these processes are often experimentally validated either in melanoma or in other cancer types^37^,^38^,^39^ (Fig. 7b and Supplementary Data 3 and 4). Accordingly, ‘locomotion' is the most over-represented Gene Ontology term among the set of predicted TEAD targets (GO:0040011, adj. *P*-value 8.05e−26).

After screening a new series of short-term melanoma cultures by assessing 18 selected genes, we identified an additional invasive culture (MM029) and confirmed that it also exhibits a high invasive propensity (Supplementary Fig. 23). Importantly, the expression of *SOX9*, *SERPINE1* and *EPHA2* was also decreased on TEADs' KD in these cells (Fig. 7a). Interestingly, KD of individual TEAD members indicates that TEADs function in a redundant manner and that the complete TEAD transcriptional network shown in Fig. 5 likely depends on the joint activity of several or possibly all four TEAD members (Fig. 7a and Supplementary Figs 24 and 25).

To establish a functional link between TEADs and melanoma cell invasion, we KD all TEADs simultaneously in eight melanoma cell cultures and measured invasive capacity and cell viability. Strikingly, a significant decrease in all three invasive cultures (MM047, MM099 and MM029) was observed on TEADs' KD (Fig. 7e–g).

Collectively, these experiments indicate that the TEADs contribute to the establishment of the invasive transcriptional cell state and its associated cellular phenotype. These data also underline the ability of the TEADs to promote a survival advantage to the melanoma-invasive cells.

### TEADs sensitize invasive cells to MAPK-targeted therapy

**Figure 8:**
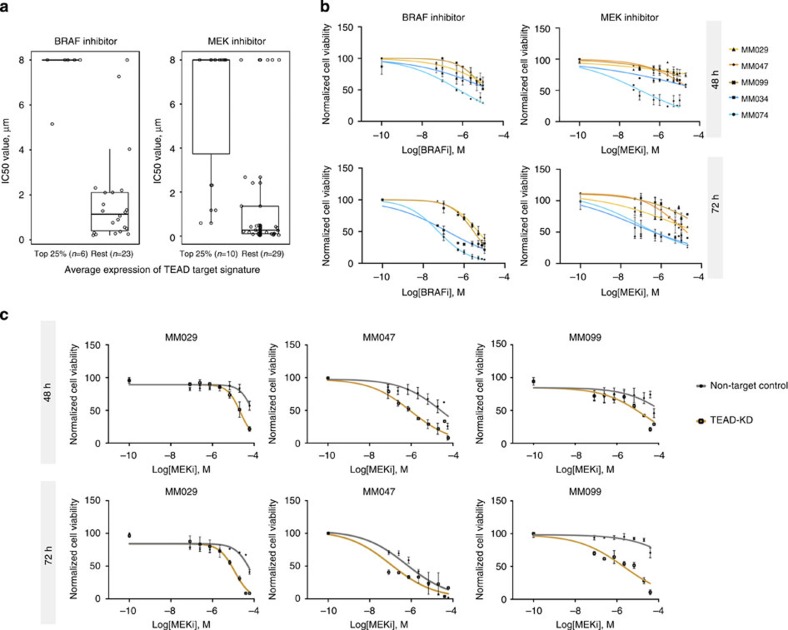
The role of the TEADs in drug resistance of the invasive melanoma state. (**a**) Analysis of CCLE data (*n*=39) shows a significant difference of IC50 values for both the BRAF and MEK inhibitors (PXL4032 and AZD6244), where cell lines with a high TEAD signature (top 25%) are more resistant compared with the other cell lines. (**b**) IC50 curves showing a strong resistance of invasive cultures (MM029, MM047 and MM099, orange shades) for both BRAF and MEK inhibitors (PLX4032 and Pimasertib) compared with two proliferative cultures (MM074 and MM034, blue shades) both at 48 and 72 h of exposure. MM047 data were not incorporated in BRAF-related plots since this culture does not harbour the V600E BRAF mutation. (**c**) IC50 shifts indicating a sensitization of the invasive lines for the MEK inhibitor measured at 48 and 72 h of treatment when treated with siRNAs against all four TEADs. All error bars represent s.e.m. and are the result of at least three biological replicates. *P*-values were determined using Student's *t*-test.

The therapeutic relevance of the two-class distinction in melanoma was recently highlighted by the observation that the two cell states are associated with differential susceptibility to MAPK pathway inhibition^11^,^40^. To test whether this drug resistance likewise correlates with high expression of the TEAD targets, we examined the Cancer Cell Line Encyclopedia (CCLE)^41^. Interestingly, there is a significant positive correlation (0.82 with *P*-value<1e−5) between expression of the TEAD target gene signature and BRAF inhibitor (PLX4032) response in 29 *BRAF*^*V600E*^-mutant cell lines. A similar trend, albeit with a lower correlation coefficient of 0.60 (*P*-value<1e-5), is also observed for resistance to the MEK inhibitor AZD6244 (*n*=39; Fig. 8a and Supplementary Fig. 26). These results suggest that TEAD-mediated transcription is one of the determinants that contribute to the increased resistance of the invasive melanoma cells to MAPK pathway inhibition.

To further confirm such a correlation experimentally, we established the IC_50_ values for both a BRAF and a MEK inhibitor (PLX4032 and Pimasertib) in several of the short-term cultures. Strikingly, invasive cultures are significantly more resistant to these inhibitors than proliferative cultures (Fig. 8b). Furthermore, simultaneous KD of all four TEADs significantly (re)sensitizes the invasive cultures to the MEK inhibitor (Fig. 8c and Supplementary Fig. 27).

Together, these results indicate that the TEADs' transcriptional network—selectively induced in melanoma cells adopting an invasive cell state—confers intrinsic resistance to MAPK therapeutics.

## Discussion

Cell state transition *in vivo* is likely to be driven by changes in the microenvironment, ultimately leading to transcriptional reprogramming^42^. The plasticity and reversibility of phenotype switching indeed favours a model in which cell state transition is dependent on reprogramming of the transcriptome rather than being dictated by the acquisition of specific DNA mutations. In agreement, we found no enrichment for specific gene mutations in the invasive or proliferative clinical samples (TCGA). Unexpectedly, when analysing copy number alterations in the TCGA cohort a 7q34 duplication was found to be enriched in the invasive samples. This region harbours 89 genes and includes the *BRAF* gene (Supplementary Note 1). Interestingly, overexpression of BRAF was recently shown to drive a rapid and reversible switch in a specific subset of invasive-related TFs^5^. Together, these data raise the possibility that, although melanoma phenotype switching is, by and large, governed through transcriptional reprogramming, specific genetic lesions may render melanoma cells susceptible to such reprogramming.

The gene expression patterns that define this transcriptional reprogramming have been defined to some extent in ref. 3 as a gene signature of 97 genes; 45 specifically expressed in invasive and 52 in proliferative cells. We have extended this gene signature to better capture the entire repertoire of genes involved in both states and identified 643 and 772 genes that are upregulated in invasive and proliferative cells, respectively. In addition, we have included (poly-adenylated) lncRNAs in the analysis by using the GENCODE annotation, classifying 17 into the invasive and 49 into the proliferative signature (Supplementary Data 1).

At a first level, our data allowed us to generate a more extended view of the transcriptional reprogramming underlying melanoma cell state transition. However, incorporating regulatory profiling and performing profound *in silico* analyses allowed us to go a step further and identify potential regulators behind this reprogramming. Particularly, by decoding the sequences of the differentially active regulatory regions, we identified SOX10 and MITF as master regulators of the melanoma-proliferative cell state. MITF has been extensively studied in the context of normal melanocyte development, where it is expressed downstream of PAX3 and SOX10 (ref. 7). In addition, it has been shown that *MITF* is often amplified and overexpressed in melanomas^43^. The MITF target prediction we present herein combines information on active genomic regions based on H3K27ac profiles with active gene expression, and includes distal enhancer–promoter interactions. Comparing our results to MITF KD data indicated that our MITF target gene prediction is more accurate than those based on ChIP-seq or microarray data alone, and includes hundreds of previously unknown MITF targets (Supplementary Fig. 28). The second proliferative master regulator is SOX10, a key regulator of neural crest cell development and melanocytic differentiation. It is expressed in nearly all primary melanomas and its overexpression causes the formation of giant congenital naevi in mice^8^. Interestingly, many genes that are part of our invasive gene signature, including *EGFR* and *TGF-ß*, are upregulated 48 h after SOX10 KD (Supplementary Fig. 29). This observation indicates that the gene regulatory network can be steered into a different attractor state simply by perturbing one of the master regulators and is consistent with the ability in melanoma to switch from a proliferative to an invasive state through transcriptional reprogramming. Moreover, although no SOX10 ChIP-seq data are available for melanoma to date, our integrative genomics approach identified a high-confidence set of direct SOX10 candidate target genes, including known targets *MITF* and *DCT* and showing a large overlap with direct MITF target genes, thus indicating a SOX10 MITF feed-forward loop.

Contrary to the proliferative state, the analysis of the invasive transcriptome puts forward AP-1 and TEAD as strong candidates for key regulators, neither of which have been directly implicated in the melanoma-invasive gene network before. However, both AP-1 and TEAD have been implicated in EMT, either separately or together, as illustrated by recent evidence supporting a direct interaction between YAP and the AP-1 family member FOS during EMT^44^. Notably, in support of our findings on TEAD, two recent studies have attributed pro-invasive roles for YAP and TAZ in melanoma^34^. We find that AP-1 and TEAD share many of their targets, indicating that these factors may act cooperatively to regulate gene expression. Consistently, independent public data from ENCODE, as well as our own predicted invasive enhancers, support the hypothesis that AP-1- and TEAD-binding sites often overlap at the same regulatory region. Note, however, that many of these predicted target regions are located at a considerable distance from potential target genes. This fact poses a challenge to associate differentially active regulatory regions with differentially expressed candidate target genes. Distal enhancers have been shown to be able to regulate their target genes even when located thousands of bp from the TSS, and with intermittent ‘bystander genes' present. By establishing correlations between enhancer and gene activity and performing 4C-seq, we have predicted and confirmed that for *SOX9* these enhancers indeed interact at a long distance by enhancer–enhancer and/or enhancer–promoter looping. Notably, the observed architecture of loops differs strongly between invasive and proliferative samples, elegantly explaining the differential expression of this target gene. This indicates that long-range chromatin interactions are dynamic and play a role in activating transcription. These results form compelling evidence for the role of the chromatin landscape in shaping the transcriptome underlying different cellular states.

One of the consequences of this complex regulatory system is that upregulation of genes involved in invasion seems to correlate with an increased therapy resistance in patients. For instance, an increase in *EGFR* and a concomitant decrease in *SOX10* expression have been linked to the development of resistance against BRAF inhibitors^1^. Here we show for the first time that the invasive melanoma state is functionally dependent on TEADs, and that blocking the activity of this family of TFs increases the sensitivity of invasive cells to MAPK-targeted drugs. On the basis of these observations we propose that the intrinsic sensitivity of melanomas to MAPK pathway inhibitors is dictated by their transcriptional cell states, which are in turn controlled by specific master regulators.

In conclusion, our study shows that integrating existing data sets with carefully designed *in vitro* experiments is a valid approach to tackle a clinically relevant cancer issue. By investigating the information flow from the genome sequence, via the chromatin landscape to the transcriptome output, we indeed gained insights into how gene regulatory networks instruct cells to adopt the phenotypically distinct invasive and proliferative melanoma states. Our results raise the possibility that intratumour heterogeneity and therapeutic sensitivity can be under the control of the cancer cell regulatory genome.

## Methods

### Analysis of publicly available microarray data

Two microarray platform gene expression compendia (Compendium A and Compendium B) were created from the publicly available gene expression data sets. Compendium A consisted of two data sets generated with Affymetrix Human Genome U133A Arrays totalling to 135 samples, while Compendium B consisted of 7 data sets generated with Affymetrix U133 PLUS 2.0 arrays and totalling to 263 samples (Supplementary Table 1). Raw intensities were downloaded from GEO, normalized with *limma*^45^ package and merged (per platform) with the COMBAT batch effect removal procedure using the *inSilicoDB*^46^ package. For both of the data sets, non-negative matrix factorization (NMF) was performed in TIGR MultipleExperiment Viewer (TMEV)^47^ using all genes with an expression s.d. above 1 (572 and 1,868 genes, respectively) across all samples. Cluster-specific gene rankings were obtained by contrasting the samples with the rest of the samples and signed −log10(adjusted *P*-values) were used for ranking the genes. Differential expression analysis was performed with *limma* package in R/Bioconductor. The rankings were analysed with GSEA^48^ using the pre-ranked analysis option. The gene signatures that are used in the GSEA included known pathways from KEGG and REACTOME; functional terms from GO; curated gene signatures from msigdb (v4); and literature mined signatures (Widmer^3^, Cheng^49^, Hoek^4^, Messina^50^), in total comprising 10,302 signatures.

### Analysis of TCGA/SKCM data

The raw count matrix composed of 375 samples was downloaded from the Firehose (stddata, timestamp: 16_03_2014, cohort: SKCM). NMF was performed using 501 genes that have expression s.d. above 1 across all samples. Once the sample clusters were identified, the same procedures as above were implemented for generating cluster-specific gene rankings and performing functional enrichment analysis. Differential expression analysis was carried out using R/Bioconductor *DESeq2* (ref. 51) package. Somatic mutation calls for 345 samples and significantly mutated genes list (Mutation Analysis (MutSig v2.0 and MutSigCV v0.9 merged result) were downloaded from Firehose (SKCM analyses, timestamp: 2014_06_12, doi:10.7908/C1668BVC). There were 343 samples that had both a cluster assignment in the NMF analysis and a somatic mutation called. These 343 samples were used to analyse whether there was an association between mutation frequency and NMF cluster assignment using a Kruskal–Wallis rank-sum test. In addition, Fisher's exact test was used to test the association between mutation status of significantly mutated genes with the NMF clusters. Bonferroni's method was used to correct for multiple hypothesis testing. Illumina Infinium Human DNA Methylation 450 data file (platform code: HumanMethylation450) was downloaded from Firehose (SKCM, timestamp 16_03_2014). Two-sample *t*-test between the proliferative and invasive groups was performed to assess statistical significance of the methylated CpGs. This test yielded 1,813 significantly methylated CpGs between the groups (*α*=1e−10). Predicted regulatory regions from the invasive and proliferative groups were intersected with the 450-K array probes. The median methylation values were computed for the regulatory regions with at least two CpG probes. Differentially methylated *cis*-regulatory modules were defined using a *t*-test with *α*=0.05.

### GENIE3

Co-expression networks from the expression data sets were generated using Genie3 (ref. 24) on the same data sets as the NMF analysis. The input list of TFs we used for GENIE3 (2,245 factors) was compiled from the factor list from the TRANSFAC Professional database combined with a list of factors from the Molecular Signatures Database (MSigDB) collection (v4). A threshold of 0.007 was used to generate the final co-expression network.

### Meta-analysis

Within each data set (TCGA, Compendium A, Compendium B and in-house RNA-seq) invasive and proliferative gene rankings were generated by comparing invasive and proliferative samples to the rest of the samples. The rankings were then combined with order statistics, used previously for gene prioritization^52^,^53^. The gene meta-ranking for each state was subsequently analysed with GSEA.

### Mosaic plots

To generate mosaic plots we used GEDI^13^ (Gene Expression Dynamics Inspector) v2.1 on our gene expression and regulatory data to generate SOMs for each sample, which allowed to visualize the high number of genes/regions in maps of size 26 × 25 tiles, where each tile can include no or several similar genes/regions. Static analysis was used with default settings. For Fig. 1d, row-median normalized expression values across 829 samples (135 Compendia A, 263 Compendia B, 375 SKCM, 11 in-house and 45 short-term melanoma cultures^6^) were combined, and genes with expression s.d. above 0.5 (1,135 genes) were plotted with GEDI. For Fig. 3b, the regulatory regions having s.d.≥1 for H3K27ac and any signal for both H3K27me3 and FAIRE after data normalization were selected (the regions with H3K27m3 and FAIRE do not necessarily have s.d.>1). This yielded a set of 55,919 regulatory regions with signal across three different regulatory data layers. The values were row-median-centred within a data set, then scaled across the data sets.

### Cell culture

Cells were all kept at 37 °C, with 5% CO_2_. The 10 primary melanoma cultures are all short-term cultures derived from patient biopsies and were described before^14^. In addition, MM029 is a novel acquired short-term culture obtained under the conditions as the other cultures. All cultures were obtained with written consent from each subject and as part of a study for which ethical approval was granted. These cultures were maintained in Ham's F10 nutrient mix (Invitrogen) supplemented with 10% fetal bovine serum (FBS; Invitrogen), 4.8 mM Ala-Gln (Sigma) and 100 μg ml^−1^ penicillin/streptomycin (Invitrogen). When performing KD experiments, antibiotics were omitted from the medium. SK-MEL-5 cells were purchased from ATCC and cultured in EMEM (Gibco) supplemented with 10% FBS (Invitrogen) and 100 μg ml^−1^ penicillin/streptomycin (Invitrogen).

### RNA-seq

All 11 melanoma cultures were plated on 15-cm plates and grown to ∼85% confluence. Similarly, when performing KD experiments cells were plated into six-well plates and grown to ∼85% confluence. Cells were then collected and prepared for RNA extraction according to the RNeasy protocol (Qiagen), yielding between 2 and 40 μg of total RNA per cell line. Quality checks were performed using the Bioanalyzer 1,000 DNA chip (Agilent) after which libraries were constructed according to the Illumina TruSeq RNA Sample preparation guide. Final libraries were pooled and sequenced on the HISeq 2000 (Illumina), generating between 15 and 30 million paired-end or single-end reads (TEAD KD).

RNA-seq reads were mapped to the genome (Gencode v18) using TopHat2 2.0.9 with Bowtie2 2.1.0 applying the *--read-realign-edit-dist* 0 option to enable combined mapping^54^. The *sensitive-local* setting for Bowtie2 was used to correct for a high percentage of mismatches at the start of a read, prompting the removal of the first base pair for each read. Read counts per gene were obtained from the aligned reads using *htseq-count* command from the HTSeq framework^55^. The Bioconductor/R packages *EDASeq*, *edgeR* and DESeq2 (ref. 51) were used for normalization and differential gene expression analysis (Supplementary Data 1 and 5).

### Gene signatures

Using a cutoff of log2 fold change ≥|1| and adjusted *P*-value ≤0.05 (R/Bioconductor package DESeq2_1.4.5), gene signatures of 643 and 772 genes were defined as invasive and proliferative signatures, respectively. These cutoffs were confirmed using GSEA analyses using a ranking of all genes based on the signed −log10 (adjusted *P*-value) of the differential expression contrasting invasive and proliferative cultures. Input sets used are the Hoek signatures (45 invasive and 52 proliferative genes). Significant correlations (FDR<0.001) and a signature-derived leading edge provided a set of 660 proliferative and 499 invasive genes. These signatures are very comparable to the ones obtained using the aforementioned arbitrary cutoffs, and thus justify their usage.

### Variant/mutation calling

Single-nucleotide variations and small insertion/deletions (INDELs) were called from the RNA-seq data with SAMTools v0.1.19+ (ref. 56). Variants observed in regions with less than 20 reads and INDELs located in homopolymer stretches of >4 bp were filtered out, as were polymorphisms matching the dbSNP138-common variants. The remaining variants were annotated with Variant Effect Predictor v2.7 (ref. 57). The following terms were used for selecting the protein-altering mutations: splice_donor_variant, splice_acceptor_variant, stop_gained, initiator_codon_variant, missense_variant, splice_region_variant, inframe_insertion, inframe_deletion, frameshift_variant.

### ChIP-Seq

All 11 melanoma samples were grown to ∼85% confluence per 15-cm dish. A total of 20 million cells per sample were collected, yielding ∼20 fractions of chromatin. ChIP samples were prepared following the Magna ChIP-Seq preparation kit using at least two chromatin fractions and 2–2.5 μg of antibody per fraction. Following antibodies were used for each ChIP: anti-histone H3 acetyl K27 antibody (ab4729, Abcam); anti-trimethyl-Histone H3 (Lys27) antibody (07-499, Millipore); anti-MITF antibody (ab12039, Abcam); anti-SOX10 antibody (sc-17342, Santa Cruz). Per sample, 5–30 ng of precipitated DNA or input was used to perform library preparation according to the Illumina TruSeq DNA Sample preparation guide. In brief, the immunoprecipitated DNA was end-repaired, A-tailed and ligated to diluted sequencing adapters (1/100). After PCR amplification (15–18 cycles) and bead purification (Agencourt AmpureXp, Analis), the libraries with fragment size of 300–500 bp were sequenced using the HiSeq 2000 (Illumina).

### FAIRE-seq

Eleven melanoma cultures were grown to ∼85% confluence per 15-cm dish. A total of 10 million cells were collected per cell line, after which cells were fixed for 10 min with 4% formaldehyde and quenched (125 mM Glycine; 0.01% Triton X-100 in PBS) for 10 min. Cells were then washed twice with PBS and pelleted in 2 ml PBS with protease inhibitor cocktail. Chromatin was collected, subjecting the cells to three lysis steps, starting with 1 ml of lysis buffer 1 (50 mM Hepes-KOH pH 7.5; 140 mM NaCl; 1 mM EDTA; and 10% glycerol) at 4 °C for 10 min and spun down at 1,300*g* for 5 min. Second, 1 ml of lysis buffer 2 was applied (10 mM Tris-HCl pH 8.0; 200 mM NaCl; 1 mM EDTA; and 0.5 mM EGTA) and left to incubate for 10 min. After another spin down, 300 μl of buffer 3 (10 mM Tris-HCl pH 8.0; 100 mM NaCl; 1 mM EDTA; 0.5 mM EGTA; and 0.1% Na-deoxycholate) was added and cells were sonicated (Bioruptor UCD-200, Diagenode) for 12 cycles of 30-s pulses. Phenol/chloroform extraction was performed using Maxtract high-density tubes (Qiagen) to separate the aqueous and organic phases. DNA was precipitated using sodium acetate (0.3 M, pH 5.2), 20 μg glycogen and 95% ethanol. The pellet was resuspended in 50 μl TE buffer and incubated at 37 °C for 1 h with 1 μl RNaseA (10 mg ml^−1^). DNA was purified using the QiaQuick MinElute kit (Qiagen). Final libraries were prepared identical to ChIP-Seq libraries.

### Candidate regulatory regions

Candidate regulatory regions in human were defined using publicly available regulatory data: DHS from ENCODE^58^, General Binding Preference models^59^, CpG islands, proximal promoters, conserved non-coding sequences, ultraconserved elements, regulatory elements from OregAnno^60^, VistaEnhancers^61^ and predicted *cis*-regulatory modules^62^ (Supplementary Table 4). The UCSC liftover tool was used to convert genome coordinates to hg19 if needed. All these features were merged. In a first step, regions having an overlap of at least 20 or 80% with insulator elements in the genome or coding exons, respectively, were removed. Next, regions with an overlap smaller then 20 or 80% with insulators or exons are split and the regions containing the insulator or coding exons were removed. Remaining regions are then filtered on the basis of size and regions <30 bp are removed. Finally, any resulting regions shorter than 1,000 bp were extended if possible to 1,000 bp in a direction that prevents overlap with an insulator or exon. The complete procedure of creating candidate regulatory regions yielded 1,223,024 regions (representing ∼35% of the genome) with average size 818 bp, which can be found as a track in the Melanoma Track Hub (see Accession codes).

### Motif and track discovery

We previously developed a tool for the Drosophila genome called *i-cisTarget*^63^ allowing the identification of the most enriched/correlated NGS tracks and motifs for a given set of genes or loci by an enrichment detection method. Here we ported this framework to the human genome, starting from a set of predefined candidate regulatory regions. These regions were scored and ranked in the same way as described for the *Drosophila* version of i-cisTarget^63^, now using 1,121 human regulatory tracks with ChIP-seq data for 247 sequence-specific TFs across 43 different cell types and conditions. These data sets were mainly obtained from ENCODE database (999 tracks) but also include ChIP-seq data published by the Taipale lab (117 tracks^64^; coordinates of peaks converted from hg18 to hg19 using the UCSC liftover tool), MITF ChIP-seq in the 501Mel melanoma cell line^21^ and four in-house tracks (ChIP-seq against p53 in MCF7 after Nutlin-3a stimulation and control^65^ and ChIP-seq against MITF in MM011 and MM031 from this study). For scoring, the maximum score of the peaks was used (signalValue or fold_enrichment from encodePeak file format or MACS2 peaks, respectively).

For motif discovery, all 1.2 Mio regions are scored with a collection of 9,713 PWMs (Position Weight Matrices or motifs) from different resources^65^. PWM scoring is performed with Hidden Markov Models, one PWM at a time, across all 1.2 Mio regions, and across all orthologous regions in 10 other vertebrate species (orthologous regions determined by the UCSC *liftover* tool). Rankings across species are integrated using order statistics. A set of co-regulated input peaks is first mapped to the 1.2 Mio regions, and for each feature (motif or track), the area under the cumulative recovery of these ‘foreground' regions is calculated (at 0.25% cutoff). The areas for all features are normalized using a Normalized Enrichment Score (AUC-μ/σ). Similar enriched motifs are clustered together using STAMP. The significance was computed using the hypergeometric test and Bonferroni's method was used to correct for multiple hypothesis testing.

### Analysis of ChIP-seq and FAIRE-seq data

ChIP-seq and FAIRE-seq reads were mapped to the genome (hg19-Gencode v18) using Bowtie2 2.1.0. The *sensitive-local* setting for Bowtie2 was used to correct for a high percentage of mismatches at the start of a read, prompting the removal of the first five base pairs of each read. The coverage of candidate regulatory regions (described above) was computed using BEDTools. Subsequently, regularized log transformation and *DESeq* function from R/Bioconductor package DESeq2 (ref. 51; DESeq2_1.4.5) were used to detect differentially active regions between the two invasive and nine proliferative samples. On the H3K27ac signal, applying threshold adjP ≤0.05 and log2FC ≥|1| lead to 13,671 regions more active in invasive samples and 7,146 regions more active in proliferative samples. These regions were then filtered using differential peaks called by MACS2 (ref. 66) algorithm (*q*<0.05, *nomodel*), with the proliferative samples as treatment and invasive samples as control. This supported differentially called regions resulting in final sets of 13,453 invasive and 6,669 proliferative regions.

MITF ChIP-seq peaks were called using MACS2 (ref. 66) algorithm with default options. Peaks were called for each replicate independently, and only those with *q*-value below 0.05 were selected for further analysis (3,907 for MM011 and 810 for MM031) and visualized in the Melanoma Track Hub (see Accession codes). Signal enrichment at each genomic locus was detected using the F-seq software^67^ (version 1.84) with default options, and identified peaks were visualized (with the exception of MITF ChIP-seq) in the Melanoma Track Hub.

### Region-to-gene assignment

To assign the differentially active regions to genes we tested several approaches. The most common is the assignment to the closest gene without considering gene expression. For this basic approach were used two different gene annotations, namely RefSeq and Gencode v18 annotations. To obtain more accurate assignment, we also applied more sophisticated approach considering differential gene expression between proliferative and invasive samples. The assignment was performed to the genes with log2FC≥|1| without considering significance (which corresponds to the gene set of 1,936 proliferative and 1,437 invasive genes). The genes that are not differentially expressed are ignored. We tested different parameters, namely distance (proximal and intronic with distance ≤10 kb from the gene, and then the closest or all the genes in the distance of 10 kb, 20 kb, 100 kb, 1 Mb or 2 Mb from the region), adjusted *P*-value of differential gene expression (0.05, 0.1 and 1), correlation between H3K27ac peak and gene expression (positive or absolute values of 0, 0.1, 0.3, 0.5 and 0.7). In order to construct the gene regulatory network depicted in Fig. 5b, candidate target regions of the four TFs were assigned to genes:

- that are within 1 Mb distance from the region
- that are expressed differentially in the invasive–proliferative contrast at the adjusted *P*-value level of 0.1 and log2FC>=|1|
- for which the expression correlates with the H3K27ac peak of the region with absolute correlation coefficient of 0.3 or more.

For Fig. 5c, the optimal parameters were selected on the basis of the GSEA enrichment results for genes ranked according to GENIE3 scores of the corresponding factor (for SOX10, MITF and AP-1 targets). For TEAD target predictions, TAZ perturbation data^25^ were used (with gene ranking based on log2FC on TAZ activation), since TEAD-based GENIE3 ranking did not result in significant enrichment.

### Analysis of the publicly available perturbation data

The raw data for SOX10 (GSE37059)^8^ and JUN and FRA1 (GSE46440)^26^ perturbations were downloaded from GEO. For the SOX10 (GSE37059) data analysis, the *limma*^45^ package was used for normalization and differential expression, while JUN and FRA perturbation data were normalized with the *aroma.affymetrix* package and differential gene expression analysis were subsequently performed with the *limma* package in R/Bioconductor. TAZ perturbation data^25^ were kindly provided by Krishna Bhat and Brian Vaillant. Genes were ranked on the basis of the log-fold change and this ranking was used for enrichment analysis using GSEA. MITF perturbation data were obtained from Supplementary Table 5 in ref. 21. The genes were ranked on the basis of the −log10 (*P*-value) for enrichment analysis.

### Circularized chromosome conformation capture (4C)

The protocol was adapted from ref. 68. In brief, 10 million cells were collected for MM047 or MM011, treated with formaldehyde and cross-linked chromatin was digested with a primary 6-bp restriction enzyme, diluted and re-ligated to fuse the ends of DNA fragments. After cross-link removal by heating a second round of digestion, using a 4-bp restriction enzyme, was followed by ligation. Inverse PCR primers specific for the viewpoint were then used for amplifying captures ligated to that viewpoint. A total of 600 ng of template was used over five PCR reactions, pooled and then purified for next-generation sequencing using two columns per sample of the High Pure PCR Product Purification kit (Roche). The primers were designed as described previously^68^. Viewpoints selected are as follows: viewpoint 2 in the promoter region of SOX9 and viewpoint 1, located in a distal enhancer region 1 Mb upstream of SOX9. The following restriction enzymes were selected for each viewpoint: EcoRI and DpnII for the Sox9 promoter and HindIII and DpnII for the distal enhancer region. The primers used during the protocol can be found in Supplementary Table 6. Sequenced reads were cleaned and mapped as described for RNA-seq with an additional removal of the primer sequences from each read. A publicly available bioinformatics software package r3Cseq (ref. 69) was used to identify interaction-enriched regions per restriction enzyme fragment. Each 4C-seq sample was processed independently. Interactions with *P*-value<0.05 were considered significant. Interactions within 1 Mb around the viewpoint were visualized as domainogram plots. Note that the overlap of predicted interactions with the H3K27ac peaks (Fig. 6a) is not always exact because of the predefined restriction sites required for 4C-seq.

### Quantitative PCR

The invasive melanoma cell lines were transfected with scramble short interfering RNA (siRNA), a pool of siRNAs against the four known TEADs or against one specific TEAD. Total RNA was harvested 72 or 96 h after transfection and extracted according to the RNeasy protocol. Reverse transcription was performed using the GoScript reverse transcription system (Promega). Alternatively, RNA was collected for qPCR on SOX10, MITF and EGFR by washing cells in ice-cold PBS, scraped and pelleted at 1,500 r.m.p. for 5 min at 4 °C. Supernatant was discarded and the pellet resuspended in Qiazol (Qiagen). RNA was extracted with the miRNeasy mini kit (Qiagen). Overall, 500 ng of mRNA was converted to cDNA with the AB High Capacity cDNA Reverse Transcription Kit (Life Technologies). Real-time quantitative PCR reactions were run on LightCycler480 (Roche) in 384-well format, using SYBR-Green Fast Universal PCR Master Mix (Applied Biosystems). Melting curve analysis confirmed the amplification of a single product while normalization was carried out with the most stable of three reference genes, assessed using the GeNorm analysis. Normalized relative fold changes of at least three biological replicates were averaged before performing a Student's *t*-test to determine significance levels. RT–qPCR primer sequences can be found in Supplementary Table 6.

### KD experiments

Cells were seeded at a density of 1 million cells per 6-cm plate or 100,000 cells per well of a 12-well plate. Transfections using Lipofectamine RNAimax transfection reagents (Life Technologies) were performed for two consecutive days with 10 nM of a pool of siRNAs against all four TEAD mRNAs or a nontargeting siControl pool (Dharmacon, D-001810-10). Alternatively, KD of single TEAD was achieved similarly by dual transfection using 10 nM of a pool of siRNAs against TEAD1 (Dharmacon, L-012603-00-0005) or a specific siRNA against TEAD2. Analysis of the KD efficiency and other assays were conducted between 72 and 120 h after the first transfection.

### Matrigel invasion assay

Matrigel basement membrane matrix gel (corning) was plated at 12.5 μg in 24-well transwell inserts (Corning) 1 day in advance and was let to solidify. Cells were collected, washed once with PBS and resuspended in serum-free medium. Overall, 100,000 cells were plated per well and left to invade for 24 h. The medium was removed and cells were stained using crystal violet (90% methanol (80%); 10% formaldehyde; and 5 g l^−1^ crystal violet) for 10–20 min. After washing 3 × with water, inserts were left to dry and any excess gel was removed from the inside of the inserts using a Q-tip. Each condition was performed in duplicate. Five pictures per transwell were taken and analysed for the presence of cells using imageJ. The average number of cells across at least three biological replicates was normalized and the significance was calculated using a Student's *t*-test.

### Cell viability assay

Cells were plated at 10,000 cells per well in a 96-well plate. After 24 h, cell viability was measured using the cellTiter-Glo luminescent cell viability assay (Promega). Each condition was performed in triplicates. Averages across three biological replicates were normalized and subjected to a Student's *t*-test for significance.

### IC50 determination

Cells were plated at 10,000 or 3,000 cells per well in a 96-well plate depending on the length of the experiment. After 24 h, a dilution series for BRAF (PLX4032, Selleck Chemicals) or MEK inhibitor (Pimasertib, Selleck Chemicals) was created ranging between 20 and 0.05 μM. Forty-eight or seventy-eight hours after stimulation, cell viability was measured. Measurements were normalized using cells treated with dimethylsulphoxide (DMSO) control as maximal viability. Each condition was performed in triplicates and final data points are the average of at least duplicate biological replicates. Curves were fitted and IC_50_ calculated using a nonlinear regression analysis in Graphpad Prism (version 6.0 for mac OS X, GraphPad Software, San Diego, CA, USA, www.graphpad.com).

### Western blot analysis

Cells were washed with ice-cold PBS and lysed in lysis buffer (50 mM HEPES; 150 mM NaCl; 1 mM EGTA; 10 mM sodium pyrophosphate (pH 7.4)) containing 100 mM NaF, 10% glycerol, 1.5 mM MgCl_2_, 1% Triton X-100, protease and phosphatase inhibitor (Roche). Extracts were incubated on ice for 20 min and spun down at 20,800*g* for 20 min. Protein concentration was determined using the BCA protein assay reagent (Pierce). Equal amounts of protein from each sample were separated with electrophoresis through SDS–PAGE and transferred to a polyvinylidene difluoride membrane (Applichem). Membranes were blocked for 1 h at room temperature in Tris-buffered saline containing 0.1% Tween-20 (T-BST) and 5% nonfat dry milk. Membranes were incubated overnight at 4 °C with primary antibody diluted in 5% nonfat dry milk in TBS-T. Proteins were detected using antibodies against TEAD1 (1/2,000, BD Biosciences, 610922), β-actin (1/50,000, Sigma, A2066), MITF (1/1,000, Abcam, ab12039), SOX10 (1/1,000, Santa Cruz, sc-17342) and EGFR (1/1,000, Cell Signaling Technology, no. 4267S). Membranes were then washed and incubated for 1 h at room temperature with peroxidase-conjugated secondary antibody (Thermo Scientific) or 2 h with horseradish peroxidase-labelled secondary antibodies (Cell Signaling Technology). Protein bands were visualized using enhanced chemiluminescence as described by the manufacturer (GE Healthcare, Amersham).

### Analysis of the melanoma cell lines from CCLE

Raw expression values for 39 cell lines were downloaded from GEO and processed using the *limma*^45^ package in R/Bioconductor platform. IC50 values for PLX4720 and AZD6244 were obtained from the Supplementary Table 4 of ref. 41. Pearson's correlation coefficient was calculated between the IC50 value of the drug and the average expression of the TEAD activity signature (which is composed of 112 genes that are predicted as TEAD targets and also show log2FC>|1| with adjusted *P*-value threshold of 0.05 in the TEAD KD experiment ( Supplementary Data 6) using *cor.test* function in R.

### Drug inhibition assays

Overall, 200,000 cells were plated per six wells. Cells were transfected to KD all TEADs as described above. On day 3, 10,000 or 3,000 cells were seeded in 96 wells in triplicates. On day 4, variable concentrations ranging between 60 and 0.05 μM of BRAF inhibitor (PLX4032, Selleck Chemicals), MEK inhibitor (Pimasertib, Selleck Chemicals) were added. Alternatively, BRAF or MEK inhibitors were added at concentrations approximating the predicted IC50 value for each individual culture to measure potential additive effect of the TEAD KD. Cells treated with DMSO were used as a control and they signify maximum cell viability. Forty-eight or seventy-two hours after stimulation, cell viability was measured as described above. All measurements were carried out in triplicates. Final data points are averages of at least three biological replicates. IC50 shift curves were generated using the appropriate analysis protocol from Graphpad Prism.

## Author contributions

S.A. obtained funding, conceived and supervised the study. A.V. performed most experiments with the help of V.C., M.D., F.R., F.V., S.C. and L.Vd.M. Z.K.A. and H.I. performed all bioinformatics analyses with the help of G.Hu. and M.F. C.H. analysed the TCGA methylation data. D.S. analysed the 4C-seq data. M.F. helped with the TCGA data and contributed to the manuscript. G.Ha. contributed to the manuscript. A.V., H.I., Z.K.A., J.-C.M. and S.A. wrote the manuscript.

## Additional information

**Accession codes:** The data generated for this study (Supplementary Tables 5 and 7) have been deposited in NCBI's Gene Expression Omnibus and are accessible through GEO Series accession number GSE60666 ( http://www.ncbi.nlm.nih.gov/geo/query/acc.cgi?acc=GSE60666). This series contains 11 Paired-End RNA-seq data sets, 11 FAIRE-seq data sets, 11 ChIP-seq data sets for H3K27ac, 11 ChIP-seq data sets for H3K27me3, 2 ChIP-seq data sets for MITF, 3 replicates single-end RNA-seq scrambled siRNA and 3 replicates TEAD siRNA. All processed data are, furthermore, available as a track hub in the UCSC Genome Browser using the following link: http://genome.ucsc.edu/cgi-bin/hgTracks?db=hg19&hubUrl=http://ucsctracks.aertslab.org/papers/melanoma_paper/hub.txt

**How to cite this article:** Verfaillie, A. *et al.* Decoding the regulatory landscape of melanoma reveals TEADS as regulators of the invasive cell state. *Nat. Commun.* 6:6683 doi: 10.1038/ncomms7683 (2015).

## Supplementary Material

Supplementary Figures, Supplementary Tables, Supplementary Notes and Supplementary References Supplementary Figures 1-29, Supplementary Tables 1-7, Supplementary Notes 1-2 and Supplementary References

Supplementary Data 1 Differential expression results of the in-house RNA-seq data. Differential expression between invasive and proliferative samples (logFC and adj. p-value).

Supplementary Data 2 Candidate regulatory regions to genes. Extended information concerning all differentially active regions based on H3K27ac analysis, with predicted upstream regulators and all the differentially expressed genes (logFC>|1|) within a 2Mb distance from these regions as well as the closest genes. Supporting information of predicted upstream regulators based on FAIRE data analysis includes only SOX10, MITF and AP1 targets, since no TEAD motif was detected.

Supplementary Data 3 Candidate TEAD target genes are annotated with expression information (in-house and public datasets), biological function and involvement in melanoma.

Supplementary Data 4 Detailed regulatory and literature information on a selected subset of TEAD target genes. For the genes that are displayed in Figure 7b the number of predicted AP1 and TEAD enhancers are presented here (together with their spatial decomposition). The genes that are mir200a or mir200b targets based on Bracken *et al*. publication is indicated in the table under the Mir-200 section. And finally, the publications (along with their PMID) that support the role of the gene in invasion and/or melanoma is indicated in the Literature section of the table.

Supplementary Data 5 Differentially expressed genes upon knock down of the TEADs. All genes differentially expressed upon knock down of all 4 TEADs versus control siRNA.

Supplementary Data 6 TEAD-target signature. The genes that are predicted as TEAD targets and have log2FC>|1| expression in TEAD-KD.
