# Supplementary material for: Decoding the regulatory landscape of melanoma reveals TEADS as regulators of the invasive cell state
Source: Nat Commun. 2015 Apr 9;6:6683. doi: 10.1038/ncomms7683 (PMC4403341; doi:10.1038/ncomms7683)
Supplement: Supplementary Figures, Supplementary Tables, Supplementary Notes and Supplementary References — Supplementary Figures 1-29, Supplementary Tables 1-7, Supplementary Notes 1-2 and Supplementary References [file ncomms7683-s1.pdf]

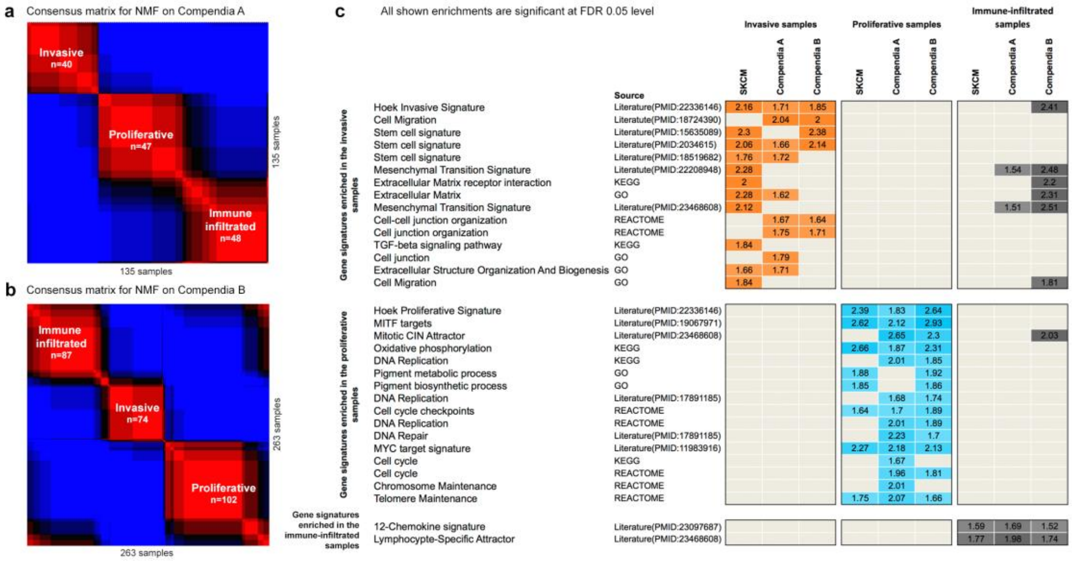

Supplementary Figure 1 – **Characterization of publicly available melanoma gene expression datasets**. Non-negative matrix factorization on compendia A (**a**) and compendia B (**b**) result in three clear sample clusters. (**c**) Characteristics of the sample clusters in each compendium are revealed by GSEA.

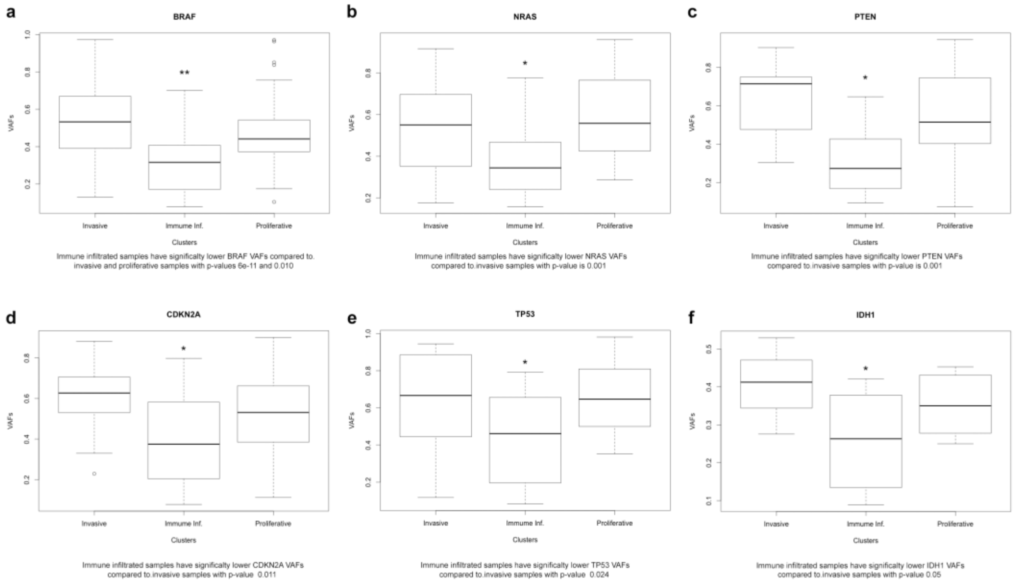

Supplementary Figure 2 – Variant allele frequencies (VAF) of known melanoma drivers across distinct sample clusters. The samples within the immune infiltrated cluster show a significantly lower VAF for BRAF (**a**) compared to both the invasive and proliferative clusters. Additionally the VAF is also significantly lower compared to the invasive cluster for *NRAS* (**b**), *PTEN* (**c**), *CDKN2A* (**d**), *TP53* (**e**) and *IDH1* (**f**). This suggests infiltration of host cells without the mutant allele.

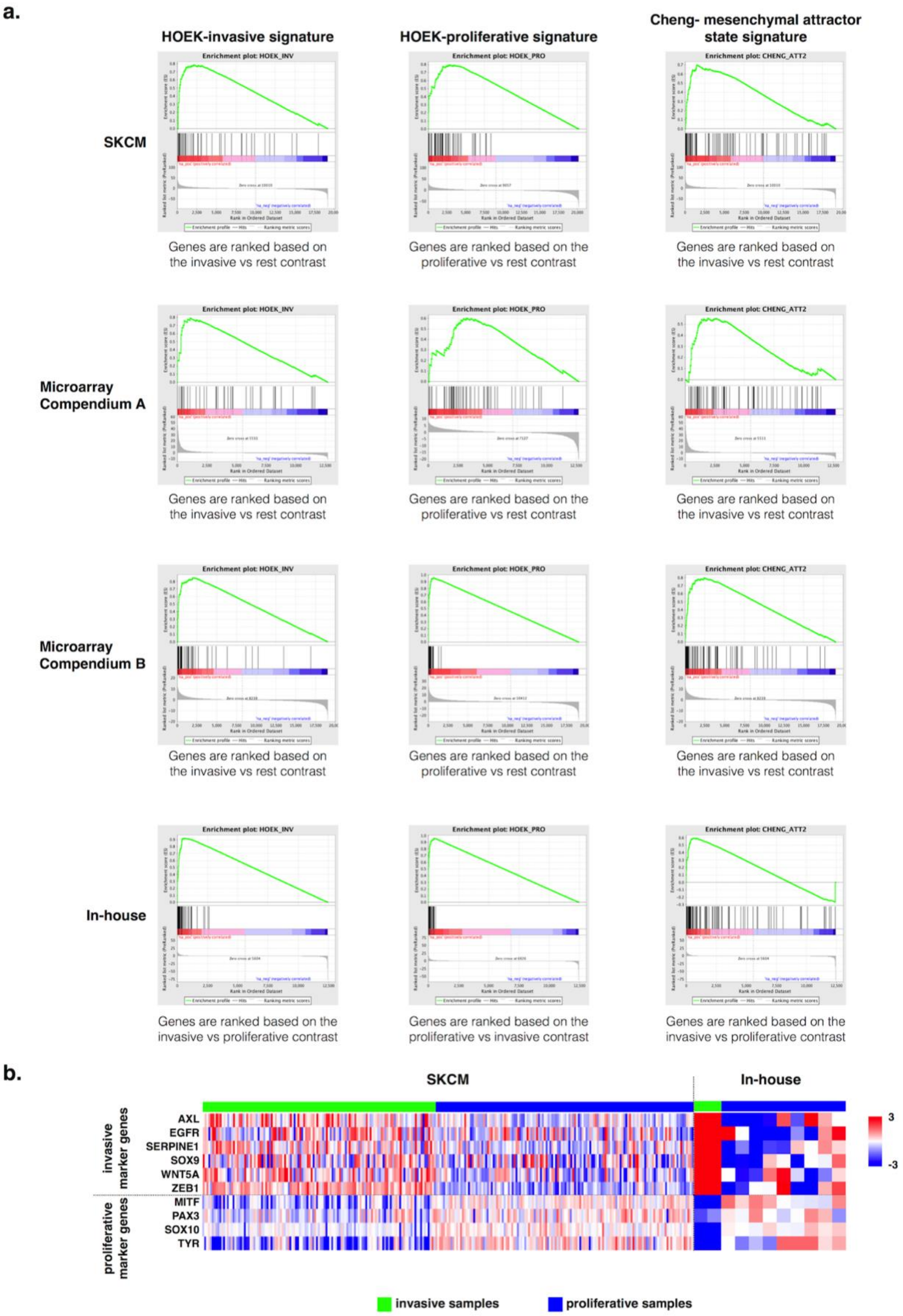

Supplementary Figure 3 - Characterization of publicly available and in-house melanoma gene expression datasets in the light of invasive/proliferative markers.

(**a**) Enrichment plots for gene rankings (x-axis) in four different gene expression datasets show significant enrichment for invasive and proliferative Hoek[1] gene signatures as well as the mesenchymal attractor state signature[2]. (b) Expression of marker genes for invasive and proliferative state correspond with the sample phenotypes in *in vivo* (TCGA –SKCM) and in *in vitro* (in-house) samples.

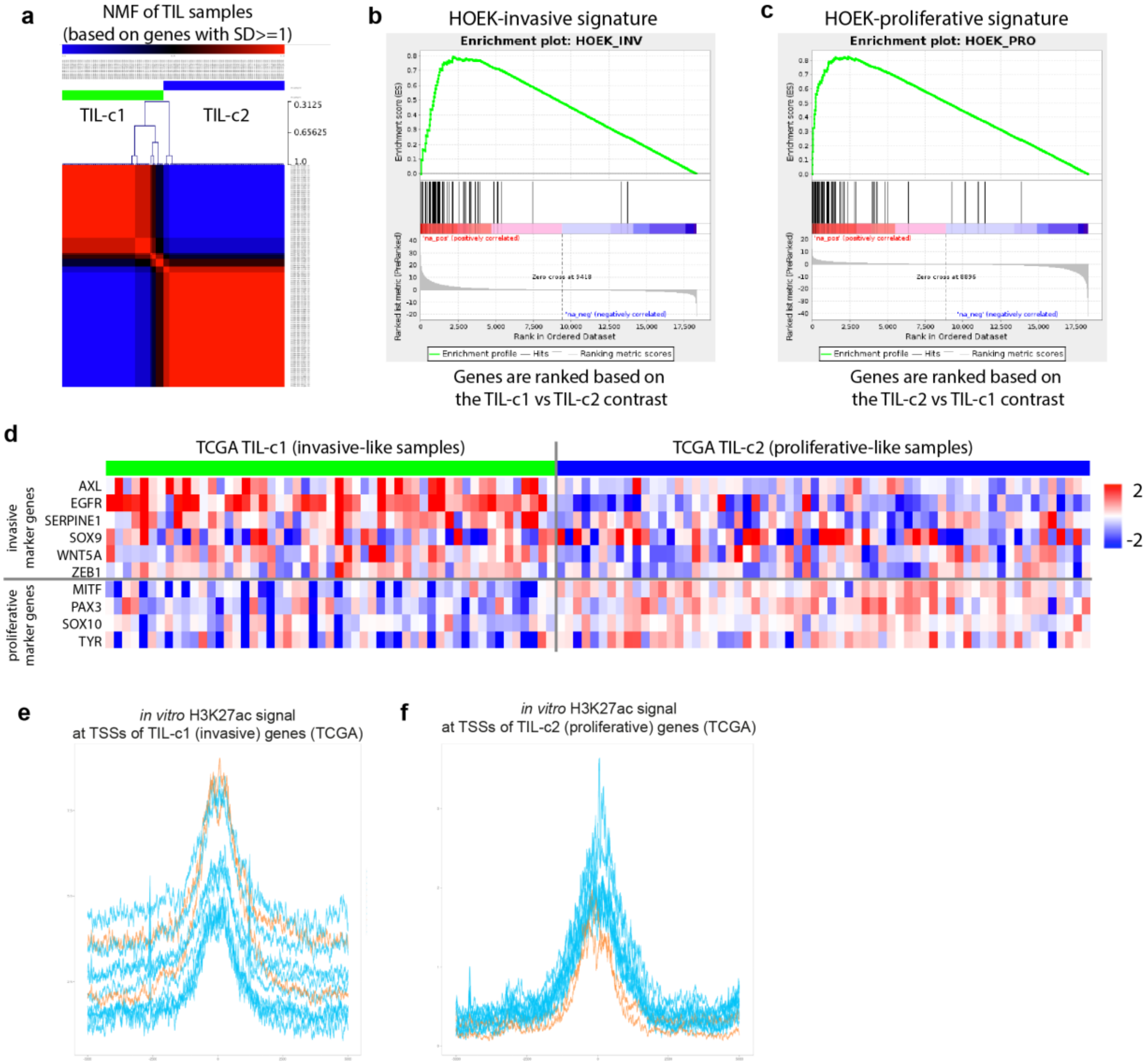

Supplementary Figure 4 – TCGA TIL group includes proliferative and invasive-like samples. (a) NMF on genes having SD>=1 within TIL group split TIL samples into two clusters and subsequent gene ontology analysis revealed that TIL-c1 genes are enriched for invasive-like processes (biological/cell adhesion, motility), while TIL-c2 genes are enriched for proliferative-like processes (melanocyte/pigment cell differentiation). Enrichment plots for gene rankings (x-axis) based on the difference between the two TIL clusters show significant enrichment for invasive (b) and proliferative (c) Hoek[1] gene signatures. (d) Expression of marker genes for invasive and proliferative state corresponds with the sample phenotype. (e) Significantly up-regulated genes in TIL-c1 (invasive) samples are mostly enriched for H3K27ac signal in invasive cell cultures, while up-regulated genes in TIL-c2 (proliferative) samples are depleted for H3K27ac signal in invasive cell cultures.

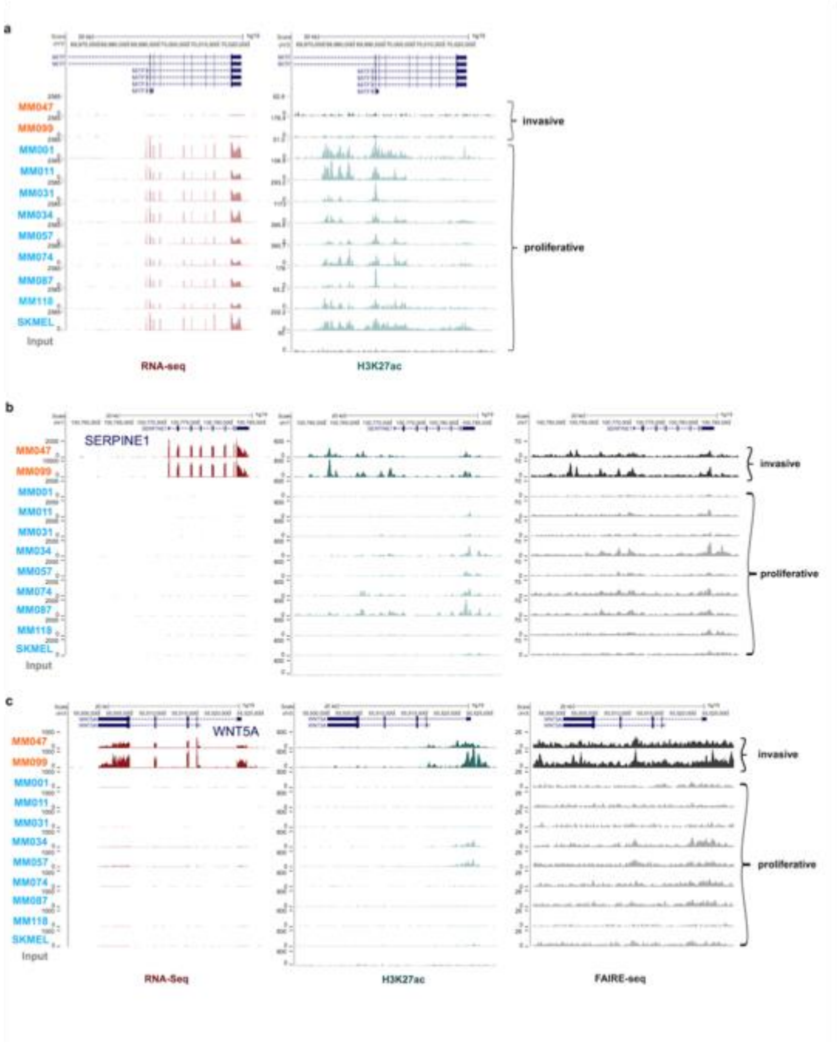

Supplementary Figure 5 - Tracks of the in-house data for three example genes involved in melanoma. (**a**) RNA-seq and H3K27ac profiles at the promoter site of the *MITF* gene, expressed exclusively in the proliferative state. (**b**) RNA-seq, H3K27ac and FAIRE-seq profiles around *SERPINE1* (**b**) and *WNT5A* (**c**), two genes involved in the melanoma invasive state.

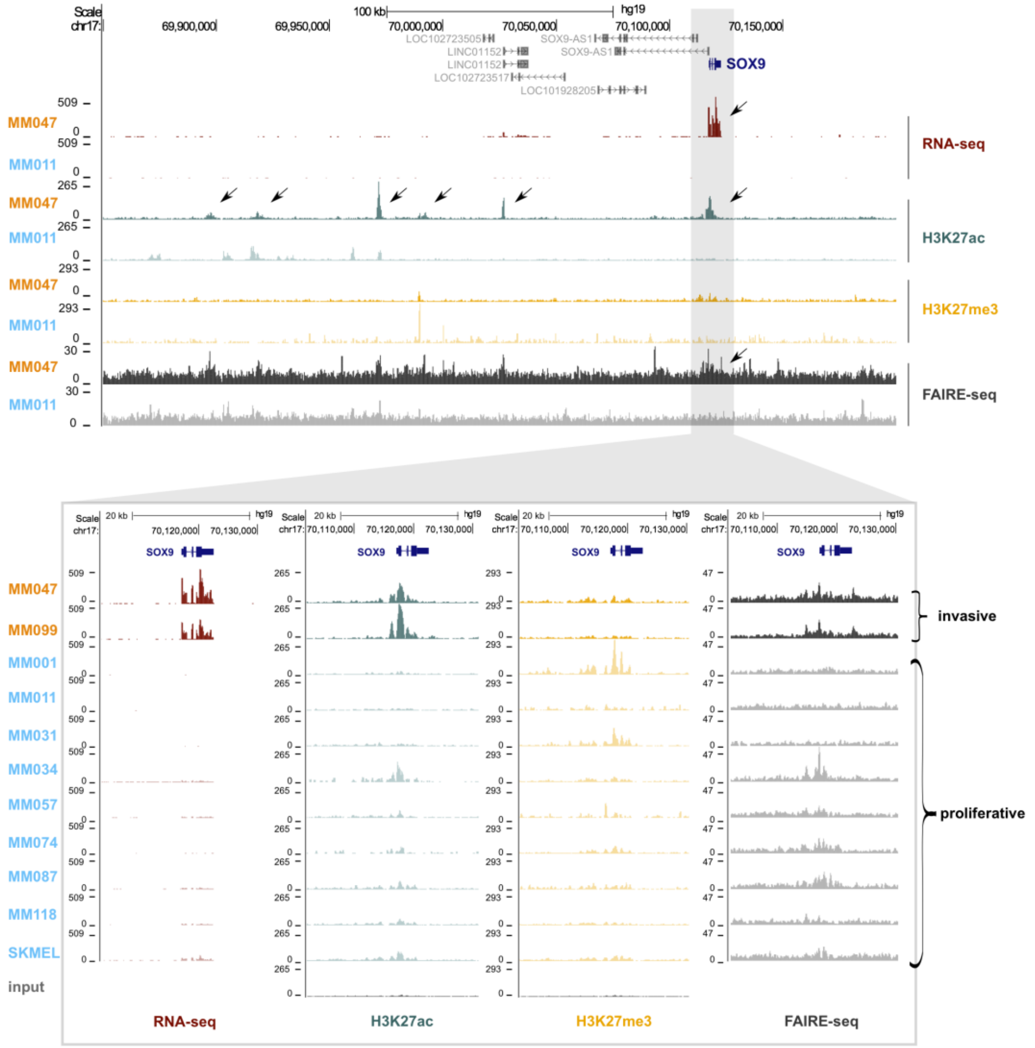

Supplementary Figure 6 - Tracks of the in-house data for SOX9. Upper panel displays a 300kb region around *SOX9*, with RNA-seq (red), H3K27ac (green), H3K27me3 (orange) and FAIRE-seq (grey) for one invasive (MM047) and one proliferative (MM011) cell line. Lower panels showing zoom in of 20 kb around *SOX9* with tracks for all 11 samples for each of the 4 profiles. Arrows indicate regions of interest that are different between proliferative and invasive states.

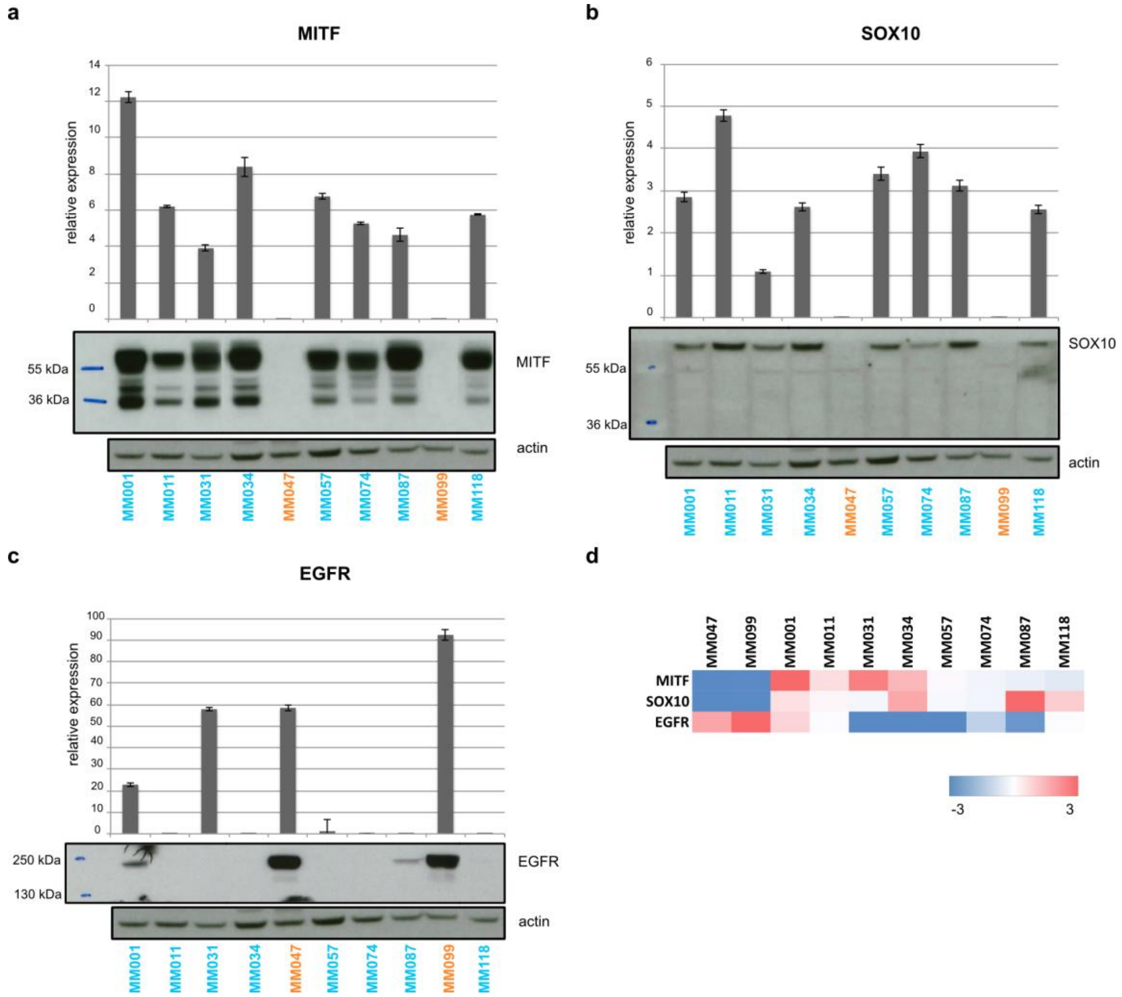

Supplementary Figure 7 – Western blot and qPCR of three markers across 11 melanoma cultures. Relative expression values of *MITF* (**a**), *SOX10* (**b**) and *EGFR* (**c**) within each culture after normalization against housekeeping genes. Additionally western blot showing the expression of their respective proteins for each of the cultures. (**d**) Heatmap showing the RNA-seq expression values (median row normalized) of these three genes.

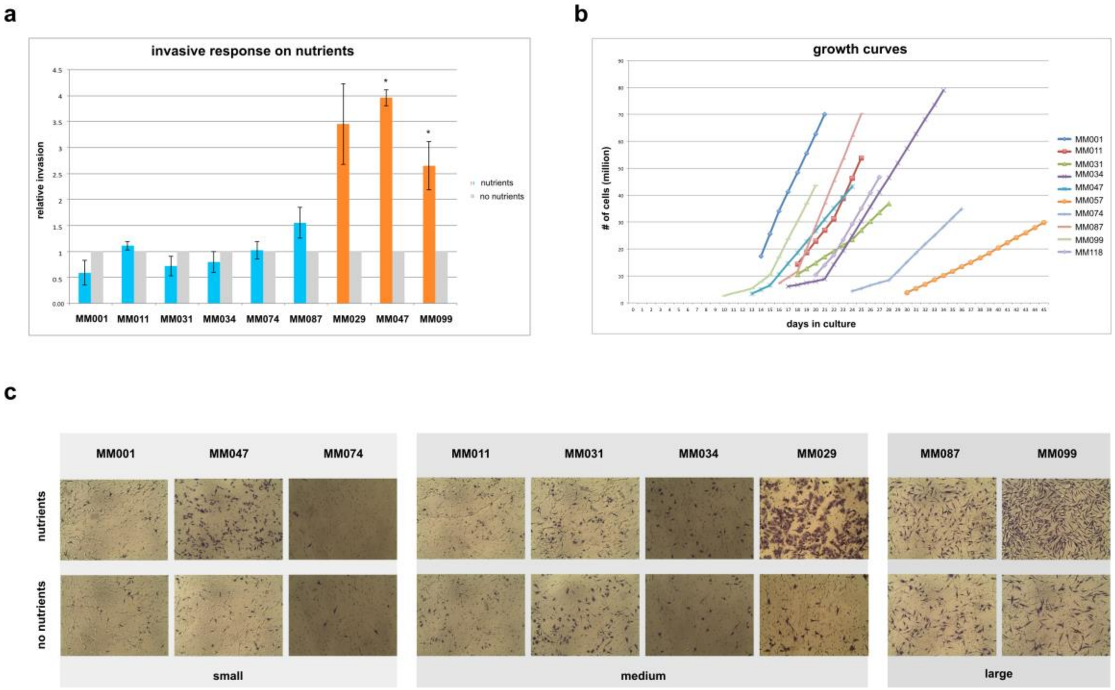

Supplementary Figure 8 – In vitro characterization of melanoma primary cultures. (**a**) Relative invasive response of 9 cultures when exposed to nutrients (orange or blue) as normalized against starved conditions (gray) within each culture. MM029, MM047 and MM099 show invasive capacity (orange, * indicates significance with $p < 0.05$). (**b**) Growth curves for each of the 10 cultures. (**c**) When exposed to nutrients only MM029, MM047 and MM099 show increased invasive capacity (top versus bottom row).

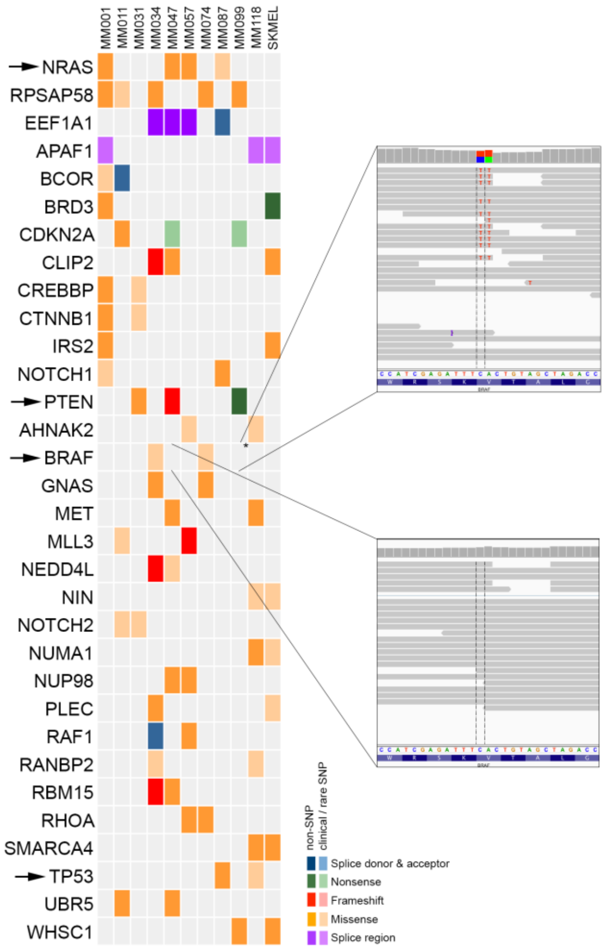

Supplementary Figure 9 – Mutational profile of in-house samples. List of potential driver genes found to be mutated in at least two of the 11 cultures. Arrows indicate genes most frequently altered in melanoma. No clear sample clusters can be identified based on the mutational profile. *The BRAF*^{V600E} mutation in MM099 is not included in the heatmap because it did not pass the depth-of-coverage filter nor the variant allele frequency filter in the variant calling pipeline (see Methods). However, inspection of the alignment with Integrative Genomics Viewer (IGV) reveals that the site indeed harbors the mutation. MM047 lacks the *BRAF*^{V600E} mutation and the IGV screenshot confirms its absence.

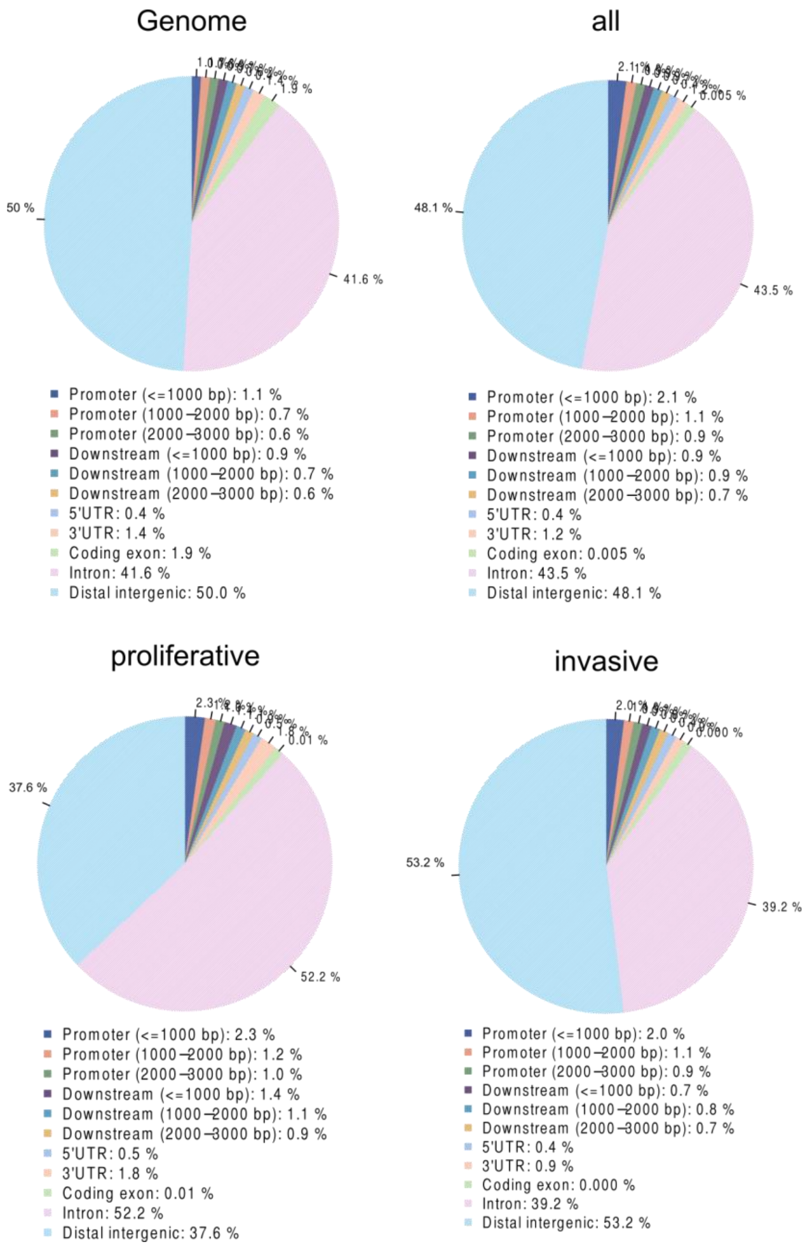

Supplementary Figure 10 – CEAS pie charts depicting the distribution of regulatory regions across the genome. The genome pie chart reflects the reference distribution of various genomic elements across the genome. In comparison, all (combination of differentially active invasive and proliferative regions based on H3K27ac (20112)) showing differences in distribution, with more regions assigned to promoters, downstream areas, 5' or 3' UTRs and introns. The last two charts reflect distribution for proliferative and invasive regulatory regions separately. It should be noted that no regions are distributed to coding exons for the proliferative and invasive regions since this assignment was based on the *i-cistarget* regions, which excludes exons a priori (see Methods).

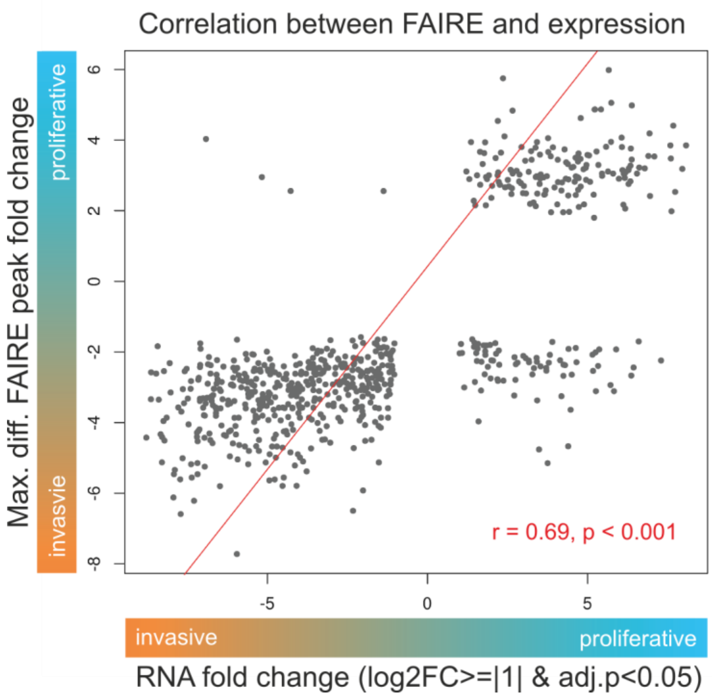

Supplementary Figure 11 - Correlation between open chromatin and expression data. Spearman correlation coefficient showing a correlation between the differential expression of genes and the differential openness of nearby regions in the genome. If a gene is significantly expressed in a proliferative sample then the differential peak with the largest fold change (called by MACS2 from FAIRE-seq data) within a space of 20kb around TSS is most likely to be significantly differentially open in the proliferative sample as well.

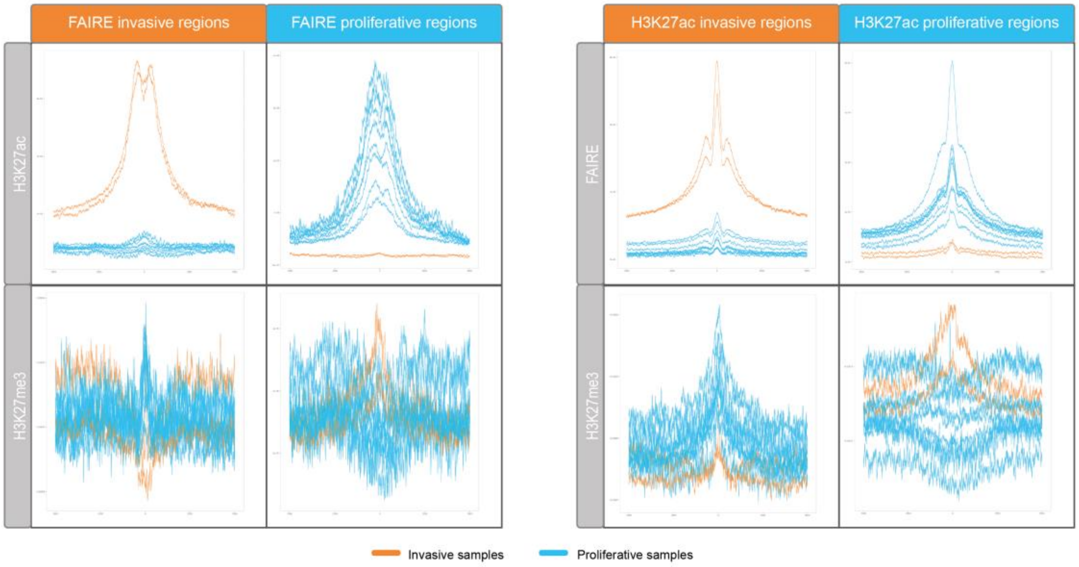

Supplementary Figure 12 – Correlation among various chromatin profiles. Left: aggregation plots centered on differentially active FAIRE regions (6408 invasive and 1223 proliferative) showing high H3K27Ac signal in the corresponding samples (e.g., high FAIRE in invasive ~ high H3K27Ac in invasive). Right: Correlations with H3K27me3 are reversed, whereby proliferative FAIRE regions show invasive H3K27me3 peaks. Right: analogous plots, now using H3K27Ac regions (13453 invasive and 6669 proliferative) to as center, showing aggregate data on FAIRE and H3K27me3.

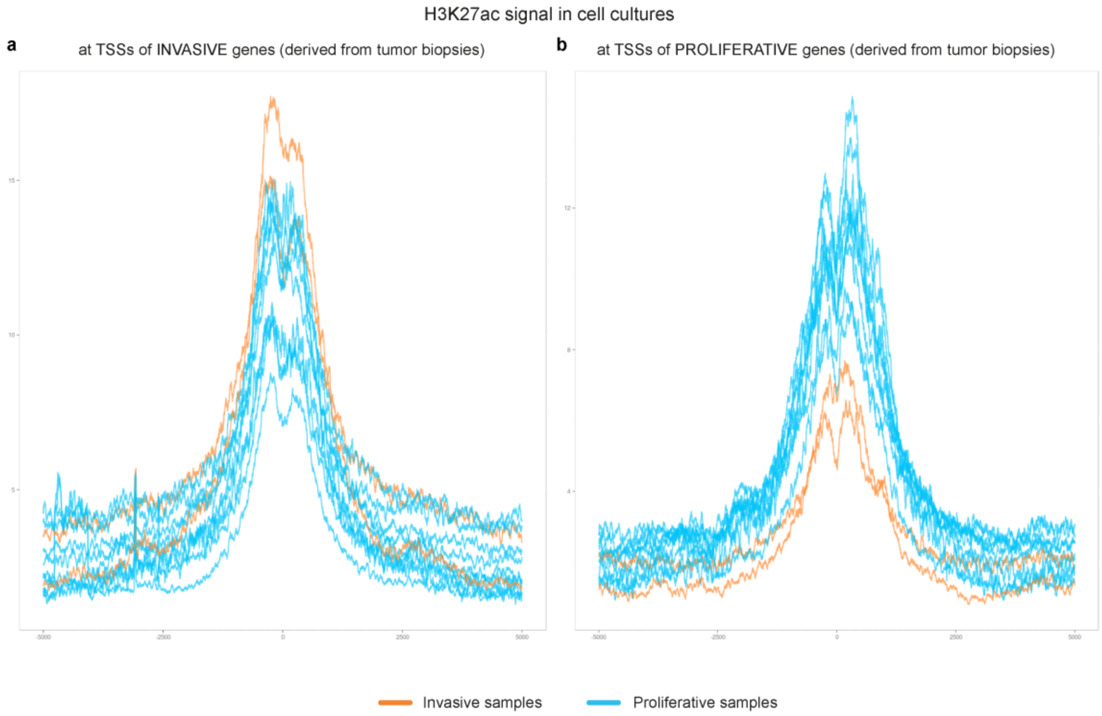

Supplementary Figure 13 – Comparison of *in vitro* H3K27ac signal with *in vivo* significantly differentially expressed genes.

Aggregation plots centered on TSSs of significantly differentially expressed genes between proliferative and invasive TCGA samples (TSSs of invasive genes on the left, TSSs of proliferative genes on the right). As expected, these aggregation plots are not so clear as the ones at TSSs of significantly differentially expressed genes derived from the cell cultures themselves (Fig. 3e), but there is still the same trend. Invasive TSSs are enriched by H3K27ac signal mostly in invasive cell cultures, while proliferative TSSs are enriched more by H3K27ac signal in proliferative cell cultures.

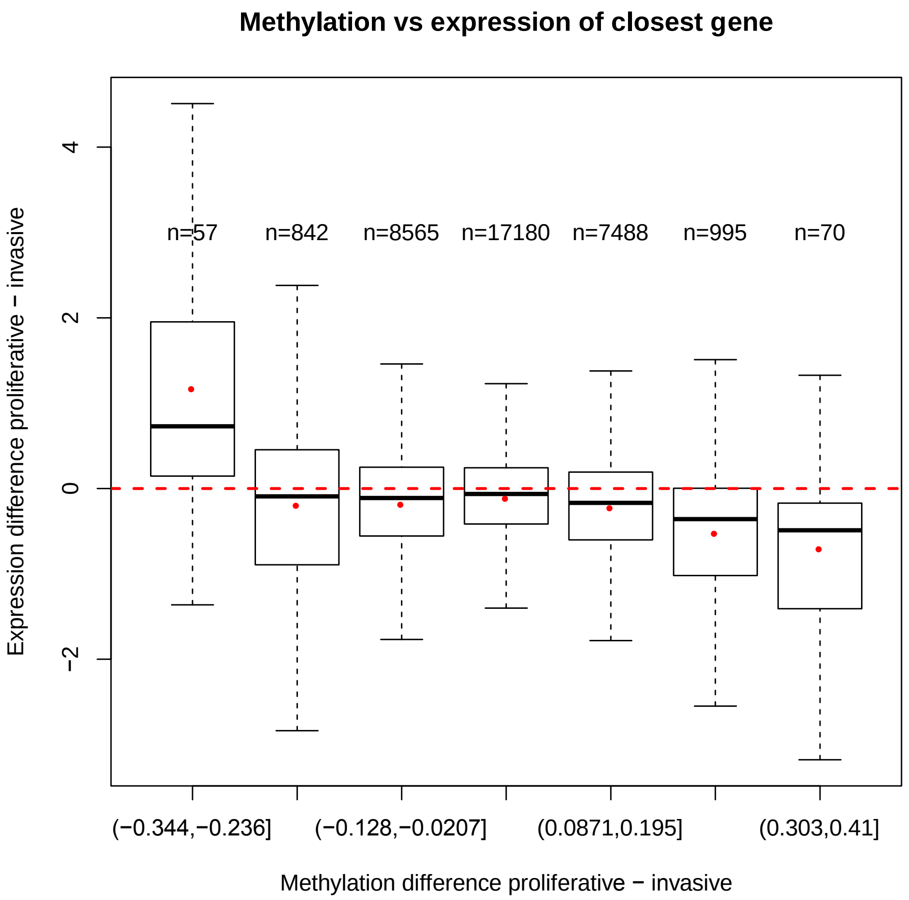

Supplementary Figure 14 – Correlation of DNA methylation and expression in TCGA samples. Boxplots showing the correlation between differentially methylated regions (proliferative versus invasive) and the expression of the closest gene. Genes with hypomethylation in proliferative samples (left) are higher expressed in the proliferative samples.

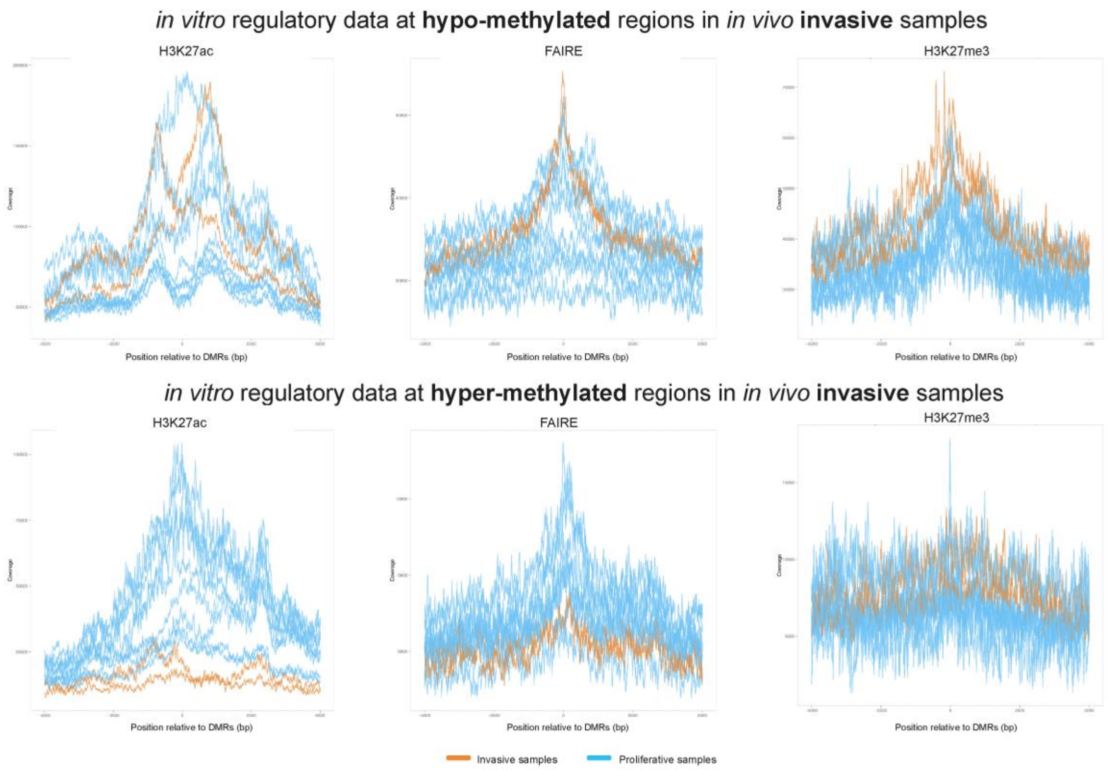

Supplementary Figure 15 – Comparison of *in vitro* chromatin profiling data at sites of *in vivo* differential methylation. Aggregation plots centered on differentially methylated regions between in vivo proliferative and invasive TCGA samples (regions hypo-methylated in invasive samples on top, hyper-methylated regions in invasive samples at bottom). Similar to regulatory signal at TSSs of proliferative/invasive genes derived from TCGA cohort, also the signal at differently methylated regions in TCGA samples is noisier but the trend is still clear.

Regions hypo-methylated regions in invasive TCGA samples are mostly enriched by H3K27ac signal in the invasive cell cultures (top left); while hyper-methylated regions in invasive TCGA samples have high H3K27ac signal in the proliferative samples (bottom left). Regions hypo-methylated regions in invasive TCGA samples have high FAIRE signal in the invasive cell cultures (top middle); while hyper-methylated regions in invasive TCGA samples have high FAIRE signal in the proliferative samples (bottom middle). There is slightly opposite tendency in H3K27me3 signal.

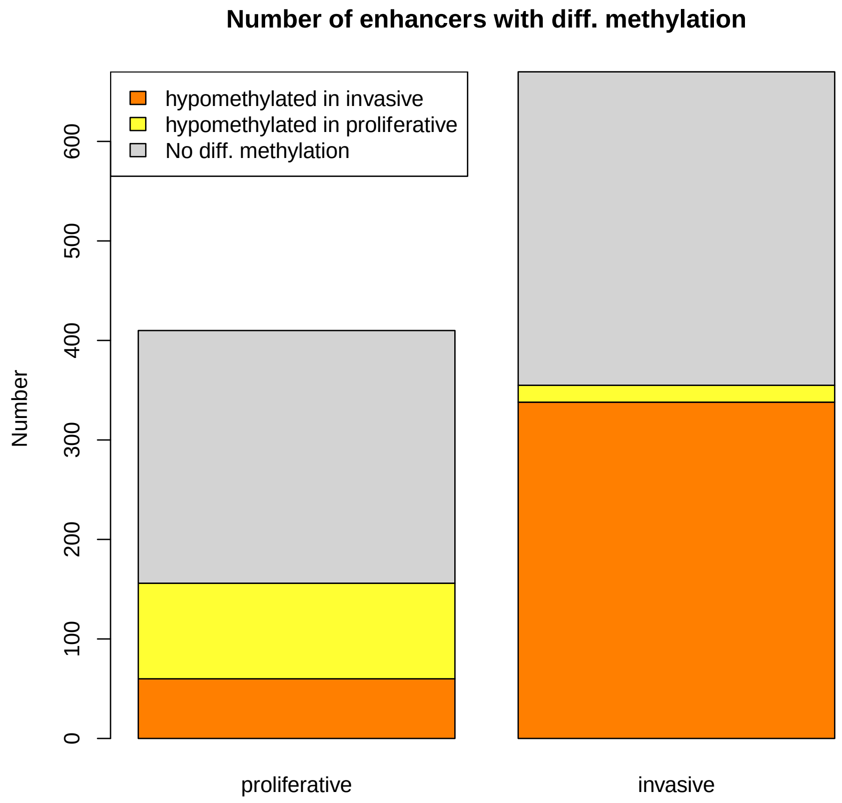

Supplementary Figure 16 – Enhancers and differential methylation. For all differentially active regulatory regions in vitro, based on differential H3K27Ac (proliferative enhancers on the left, invasive enhancers on the right), the number of hypomethylated regions is plotted. Many invasive enhancers in culture (right) show also signs of activation in vivo, as they are hypomethylated. The number of enhancers that can be assessed (see y-axis) is limited because only enhancers that have minimally two Illumina probes are included.

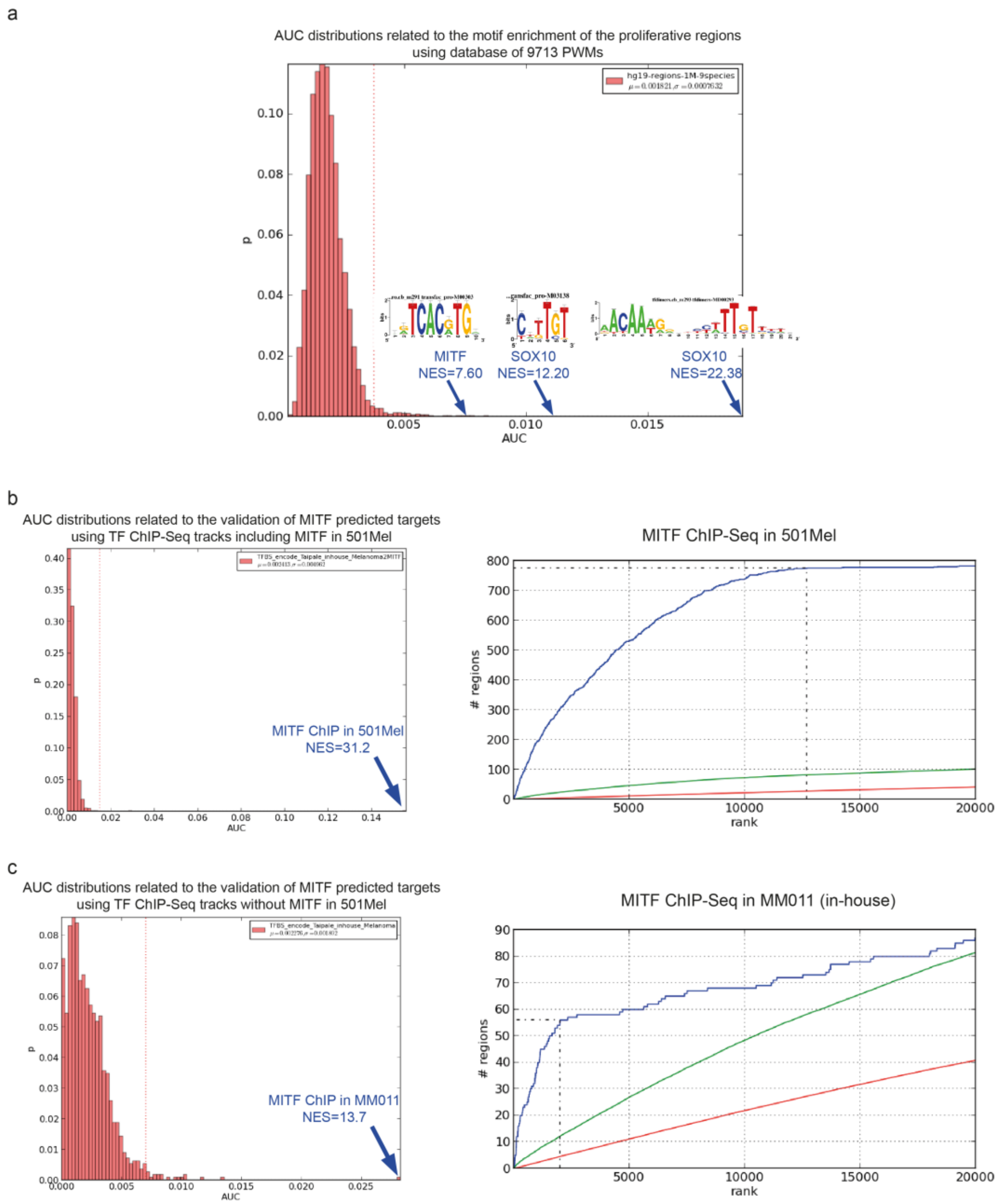

Supplementary Figure 17 – Motif and track discovery results for the proliferative enhancer signature. **(a)** Motif discovery on proliferative regulatory regions shows enrichment of MITF motifs and of the SOX10 monomer and dimer motifs. **(b)** The predicted MITF target regions are used as input for track discovery. Histograms shows that the strongest enrichment is found for the MITF ChIP-seq track from Strub *et al*[3] (x-axis, "rank"). The scores used in the histogram are calculated as the area under the recovery curve, shown on the right. Red recovery curve is the average across all rankings (one ranking per ChIP-seq track). The green curve is 1.96 std. above the mean curve. **(b)** Our in-house ChIP-seq data is also found as strongest enriched in the track database (when the Strub et al. data set is not included, otherwise in-house is second best). Right shows cumulative recovery curve along in-house MITF ChIP-seq (x-axis) for predicted MITF target regions (y-axis) (NES = 13.7). These analyses are analogous to earlier described methods i-cisTarget[4], and iRegulon[5].

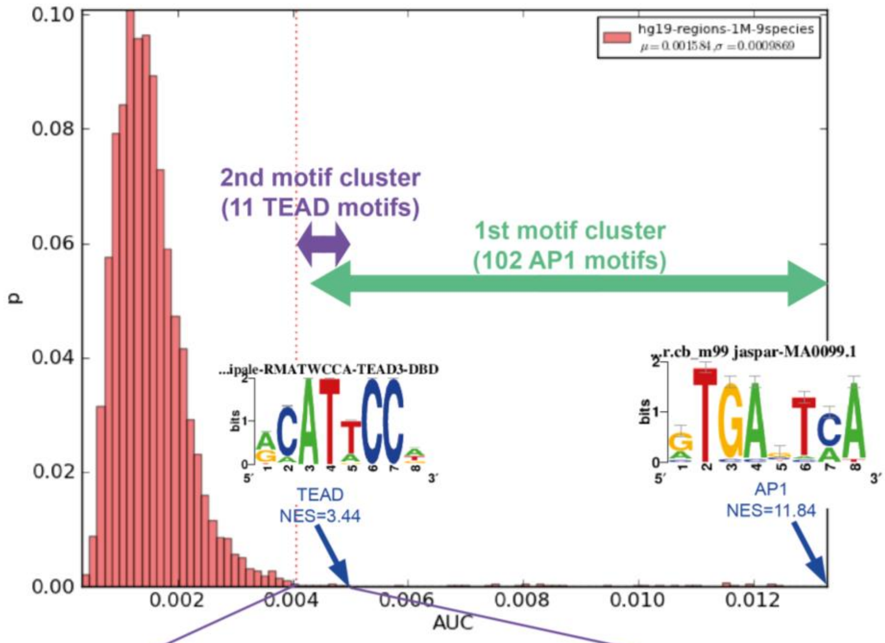

TEAD motifs (NES>=2.5)

Motif	Library source	NES	Logo
taipale-RMATWCCA-TEAD3-DBD	Taipale (Jolma et al., 2013)	3.44	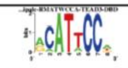
taipale-RCATTCCNNRCATTCCN-TEAD3-DBD	Taipale (Jolma et al., 2013)	3.25	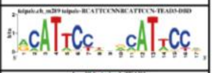
factorbook-TEAD1	Factorbook (Wang et al., 2012)	3.17	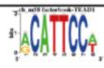
homer-M00203	HOMER (Heinz et al., 2010)	3.15	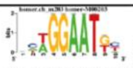 (rc)
taipale-RCATTCCNNRCATWCCN-TEAD1-full	Taipale (Jolma et al., 2013)	3.11	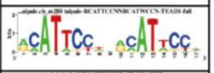
taipale-NRCATTCCWN-TEAD4-DBD	Taipale (Jolma et al., 2013)	3.02	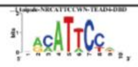
swissregulon-TEAD1.p2	SwissRegulon (Pachkov et al., 2013)	2.87	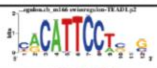
jaspar-PF0079.1	Jaspar (Portales-Casamar et al., 2010)	2.56	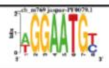 (rc)
transfac_pro-M01305	TRANSFAC PRO (Matys et al., 2006)	2.52	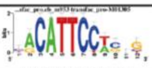
taipale-NRMATWCCWN-TEAD1-full	Taipale (Jolma et al., 2013)	2.52	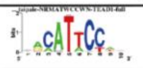
homer-M00204	HOMER (Heinz et al., 2010)	2.51	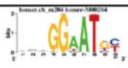 (rc)

Supplementary Figure 18 – Motif enrichment results the invasive enhancer signature finds enriched AP1 and TEAD motifs. Distribution of the area under the curve results for all 10K motifs tested using all invasive candidate regions as input set. The arrows indicate the first motif found for AP-1 and TEAD and their respective enrichment scores. All motifs found in between correspond to alternative AP-1 motifs. Table: an overview of the various TEAD motifs found enriched with their respective origins, NES and motif logos.

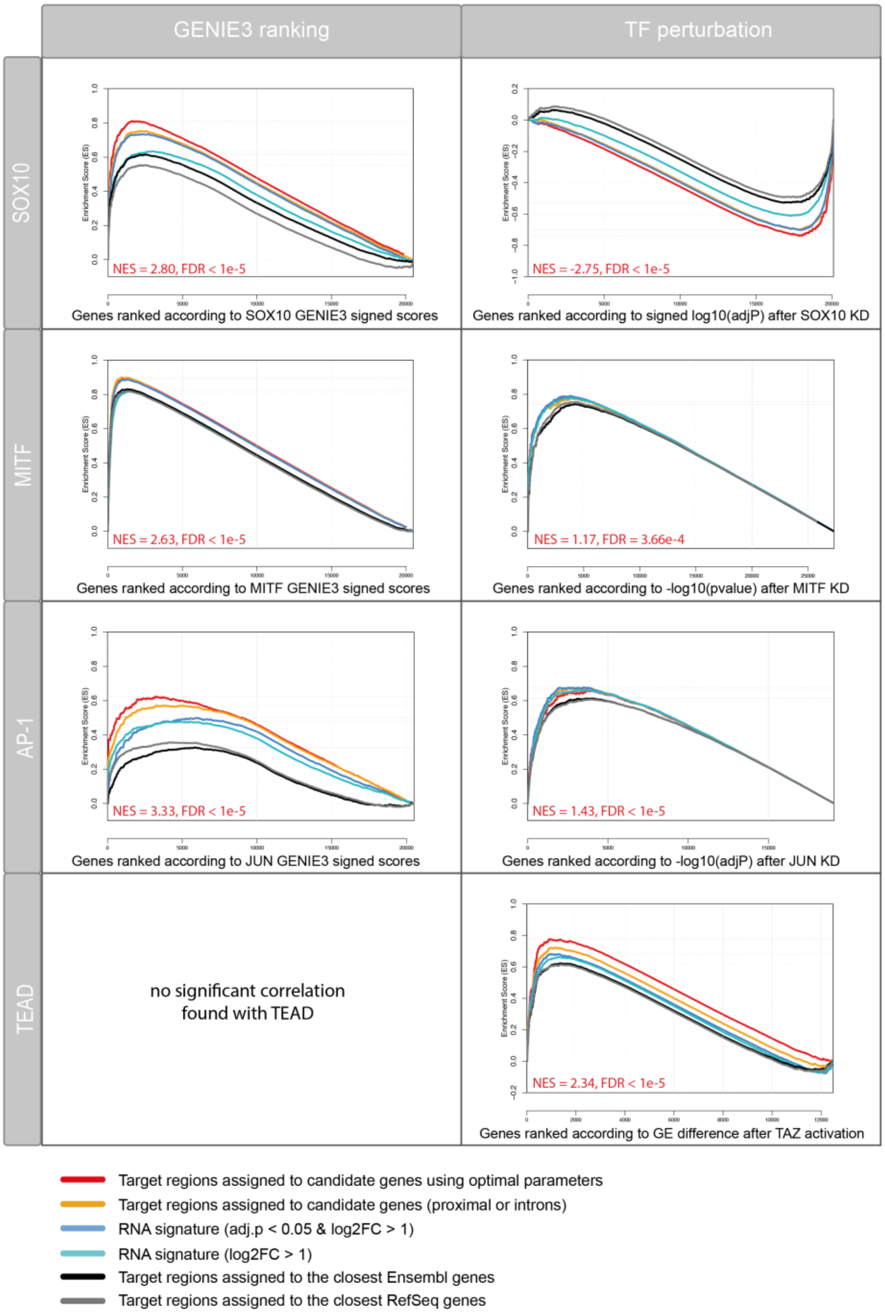

Supplementary Figure 19 – Network validation using Gene Set Enrichment Analysis (GSEA). We used GSEA to validate the predicted target sets for each master regulator using publicly available perturbation data. For the left column, all genes are ranked according to the GENIE3 signed scores for differential expression across the TCGA cohort. On the left is the highest co-expression with the factor. In the right column, the gene ranking is based on the -log10(pvalue) or the logratio for perturbation over control. Perturbation data are for MITF KD, SOX10 KD, FOSL1 KD, and TAZ constitutive activation. Optimal region-to-gene association parameters used for the gene sets represented by red curves are: SOX10 [d=100kb & closest, ge=0.05, corr=0.1], MITF [d=2Mb, ge=0.05, corr=0.3], AP-1 [d=20kb, ge=1, corr=0.1], TEAD [d=100kb & closest, ge=1, corr=0.1 & abs].

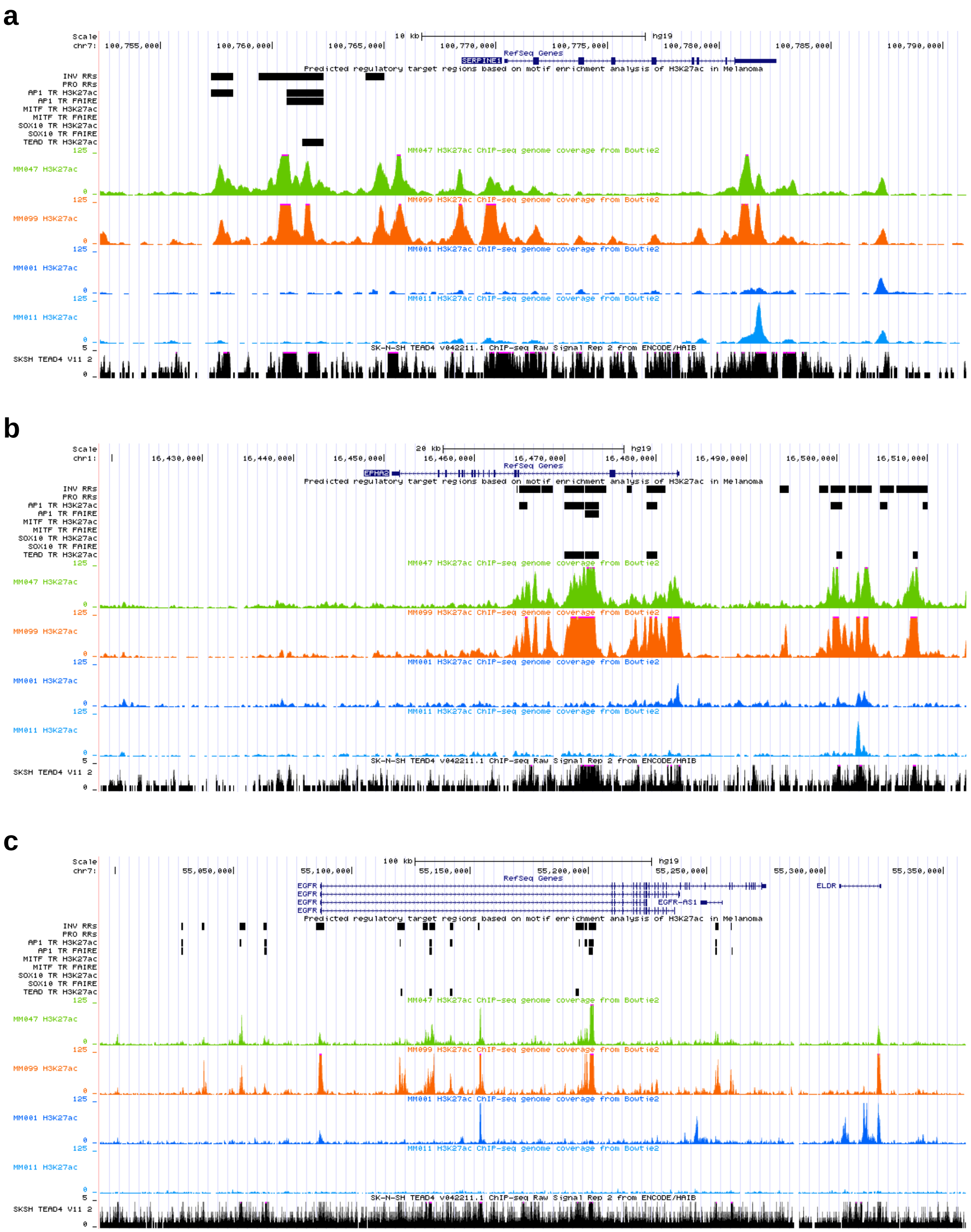
a
Scale
chr7:
100,755,000
100,760,000
100,765,000
10 kb
hg19
100,770,000
100,775,000
100,780,000
100,785,000
100,790,000
RefSeq Genes
SERPINE1
Predicted regulatory target regions based on motif enrichment analysis of H3K27ac in Melanoma
INV RRs
PRO RRs
AP1 TR H3K27ac
AP1 TR FAIRE
MITF TR H3K27ac
MITF TR FAIRE
SOX10 TR H3K27ac
SOX10 TR FAIRE
TEAD TR H3K27ac
MM047 H3K27ac
MM047 H3K27ac ChIP-seq genome coverage from Bowtie2
MM099 H3K27ac
MM099 H3K27ac ChIP-seq genome coverage from Bowtie2
MM001 H3K27ac
MM001 H3K27ac ChIP-seq genome coverage from Bowtie2
MM011 H3K27ac
MM011 H3K27ac ChIP-seq genome coverage from Bowtie2
SKSH TEAD4 V11 2
SK-N-SH TEAD4 v042211.1 ChIP-seq Raw Signal Rep 2 from ENCODE/HAIB
b
chr1:
16,430,000
16,440,000
16,450,000
20 kb
16,460,000
16,470,000
16,480,000
16,490,000
16,500,000
16,510,000
EPHA2
c
chr7:
55,050,000
55,100,000
100 kb
55,150,000
55,200,000
55,250,000
55,300,000
55,350,000
EGFR
EGFR-AS1
ELDR

d

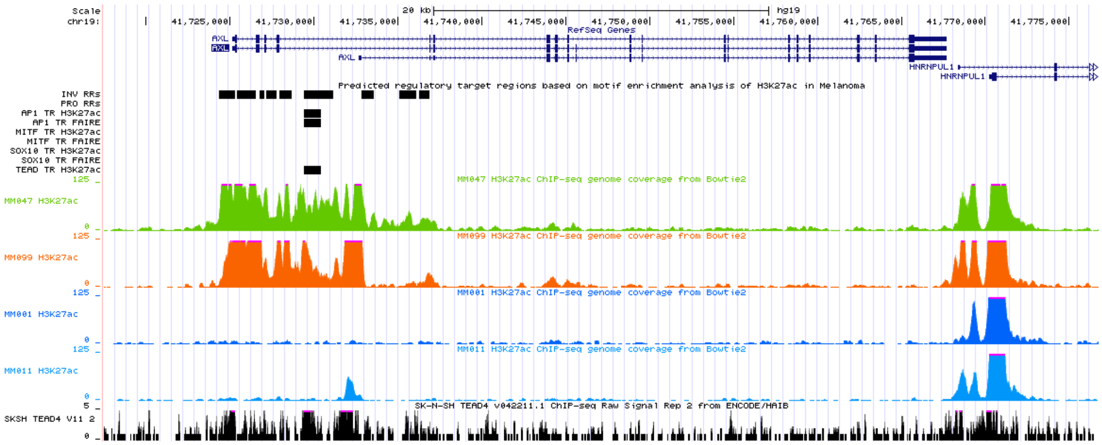

Supplementary Figure 20. Chromatin marks around (a) SERPINE1, (b) EPHA2, (c) EGFR and (d) AXL genes – UCSC screenshots display the gene loci of interest with in-house tracks (predicted regulatory target regions, H3K27AC tracks for MM047, MM099, MM001, and MM011) and TEAD ChIP-seq data from ENCODE (SKSH TEAD4 V11). Additional genes can be investigated through the UCSC track hub (see Methods).

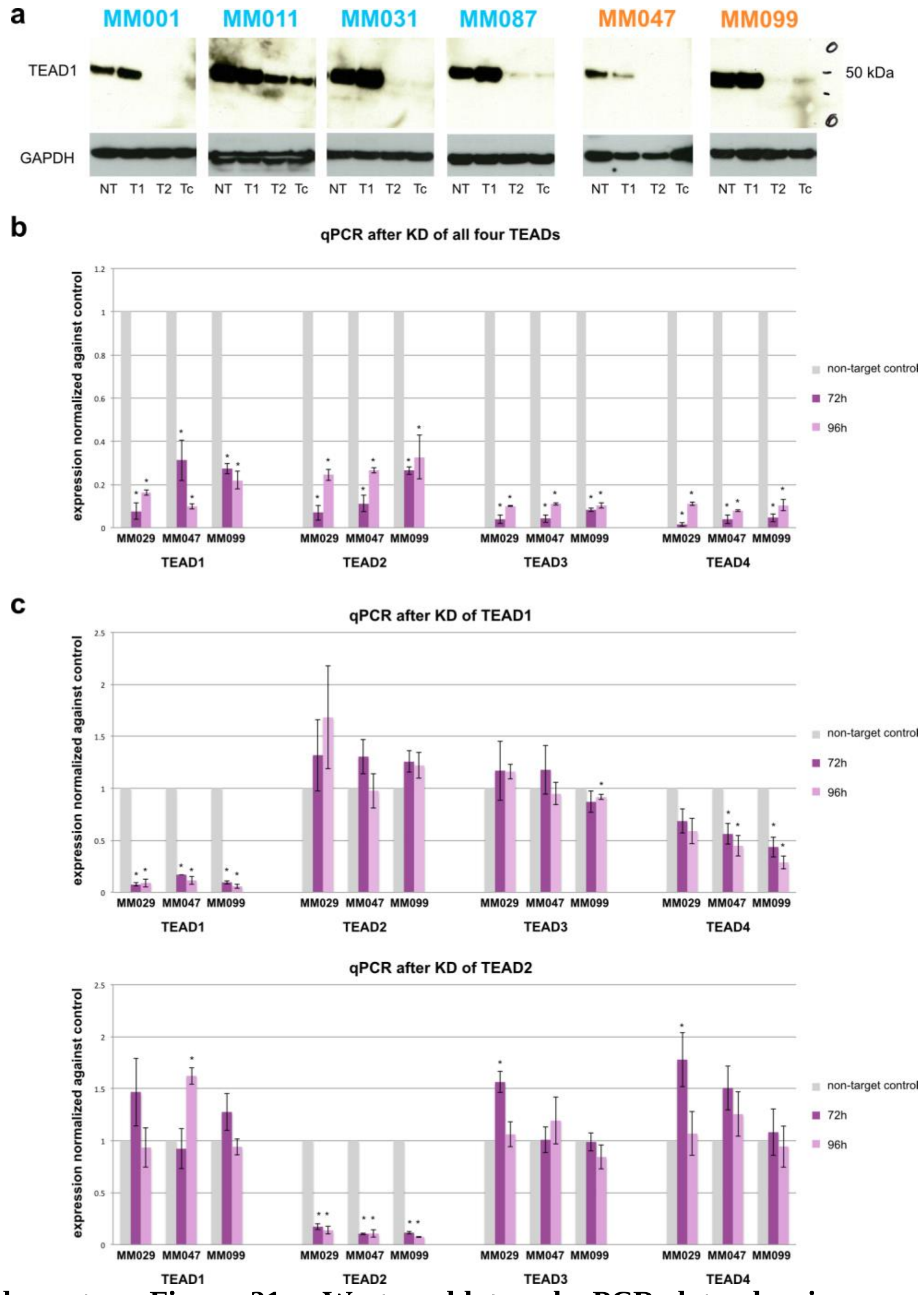

Supplementary Figure 21 – Western blot and qPCR data showing expression of TEAD upon combined or single TEAD knock down. **(a)** Western blot on TEAD1 shows a successful knock down in 6 cultures. **(b)** qPCR data showing the significant knock down of all four TEADs for the invasive cell lines. (**c**) qPCR data showing the significant knock down of targeted TEAD1 (top) or TEAD2 (bottom), additionally showing some some significant changes in expression amongst the non-targeted other TEADs. Average of three biological replicates. Asterisk (*) indicates significance of p-value < 0.05.

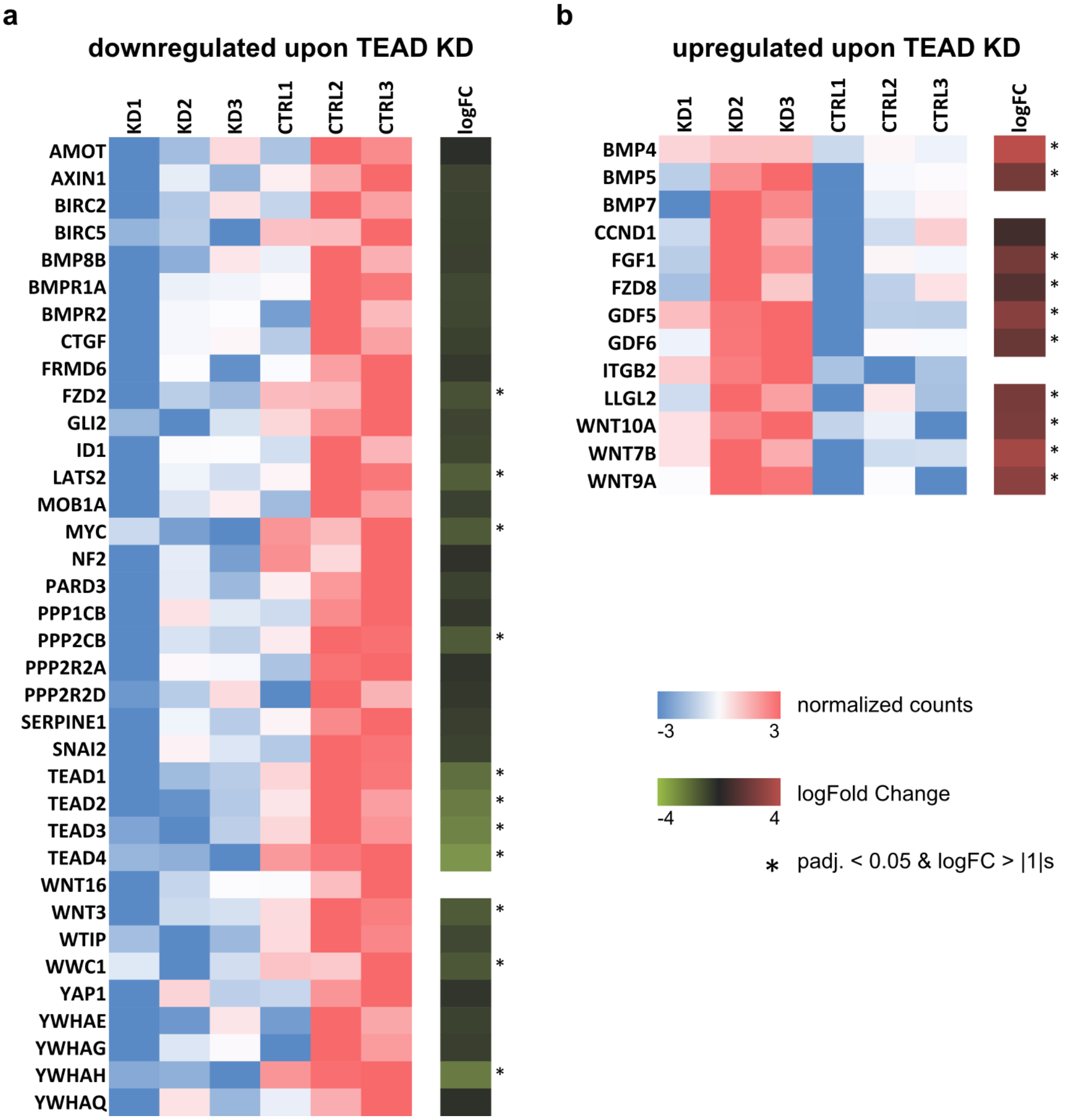

Supplementary Figure 22 – Hippo signaling members changing expression upon TEAD KD. Members of the Hippo signaling pathway (KEGG) that are down-regulated upon TEAD KD **(a)** or that are up-regulated upon TEAD KD **(b)**. Genes significantly differentially expressed (adjusted p-val < 0.05 and log2(fold change > |1|) are indicated by an asterisk (*).

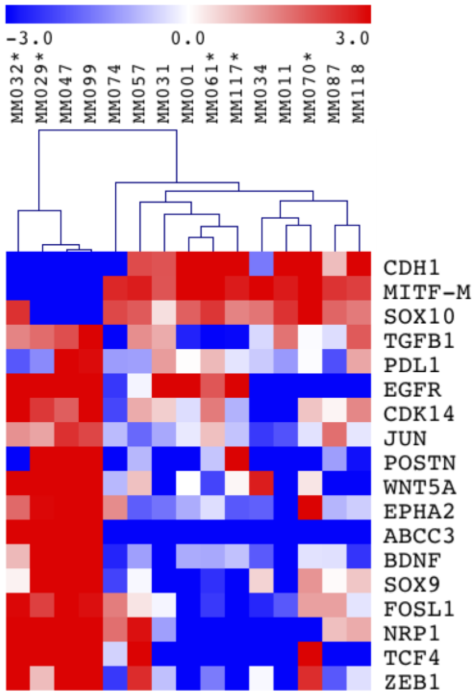

Supplementary Figure 23 – q-RTPCR on a small screen results in identification of 2 additional invasive and 3 proliferative samples. The heatmap is generated based on q-RTPCR measurement of a set of invasive and proliferative marker genes. Samples are row normalized, median centered and clustered using TMEV analysis package. Additional samples (which are not included in the initial analysis) are indicated with an asterisk (*). Among those, MM029 and MM032 are predicted to be invasive and MM061, MM117 and MM070 are predicted to be proliferative.

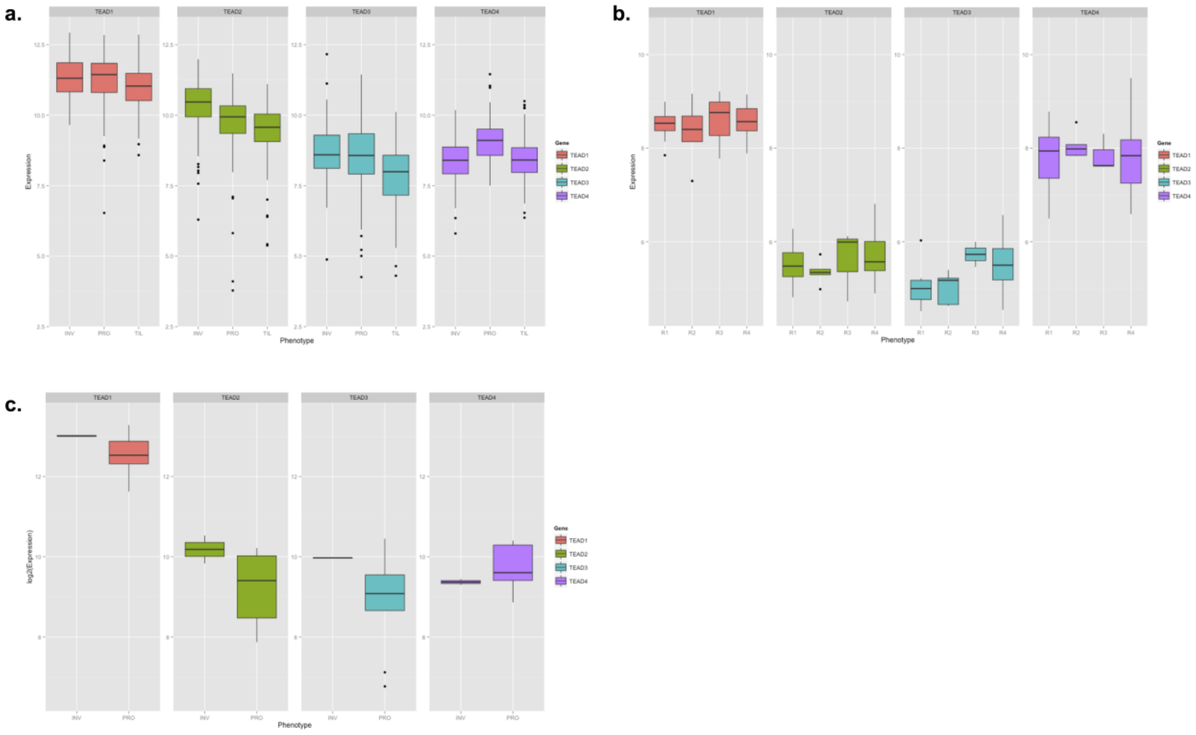

Supplementary Figure 24 - Expression of TEAD genes across (a) TCGA-SKCM, (b)CCLE and (c) in-house datasets. Overall TEAD1 expression is the highest among all TEADs. (**a**) Normalized and log2-scaled expression values from TCGA-SKCM dataset for all four TEAD genes are shown in the barplots. For each gene, the expression further stratified into samples belonging to different cell states. (**b**) Normalized and log2-scaled expression values from CCLE dataset (across 29 BRAF V600E-mutant melanoma cell lines) for all four TEAD genes are shown in the barplots. For each gene, the expression further stratified according to BRAFi resistance (R1 is for IC50-values between 0 and 2, R2 is for IC50-values between 2 and 4, R3 is for IC50-values between 4 and 6, and R4 is for IC50-values between 6 and 8). (**c**) Normalized and log2-scaled expression values from TCGA-SKCM dataset for all four TEAD genes are shown in the barplots. Here the classification for each gene is based on cell states.

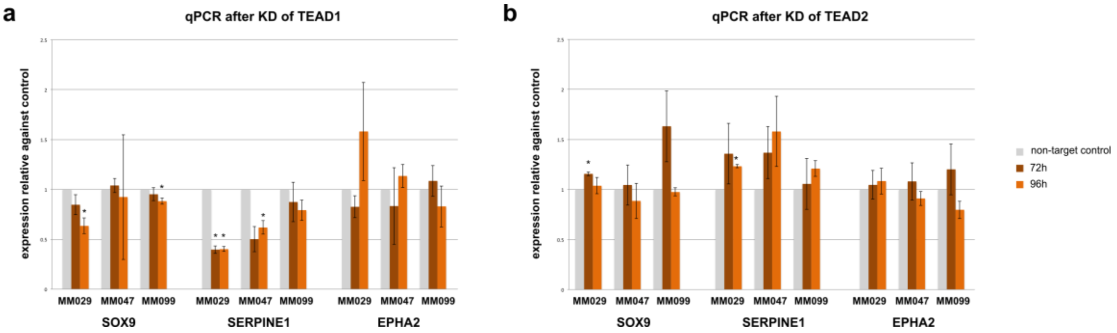

Supplementary Figure 25 - Expression for three TEAD predicted targets after knock down of a single TEAD. (**a**) qPCR results showing expression values for SOX9, SERPINE1 and EPHA2 after specific knock down of TEAD1. Aall values are normalized against cells transfected with non-target control siRNA. Asterisk (*) indicates significance with p-value < 0.05. (**b**) qPCR results showing expression values for SOX9, SERPINE1 and EPHA2 after specific knock down of TEAD2.

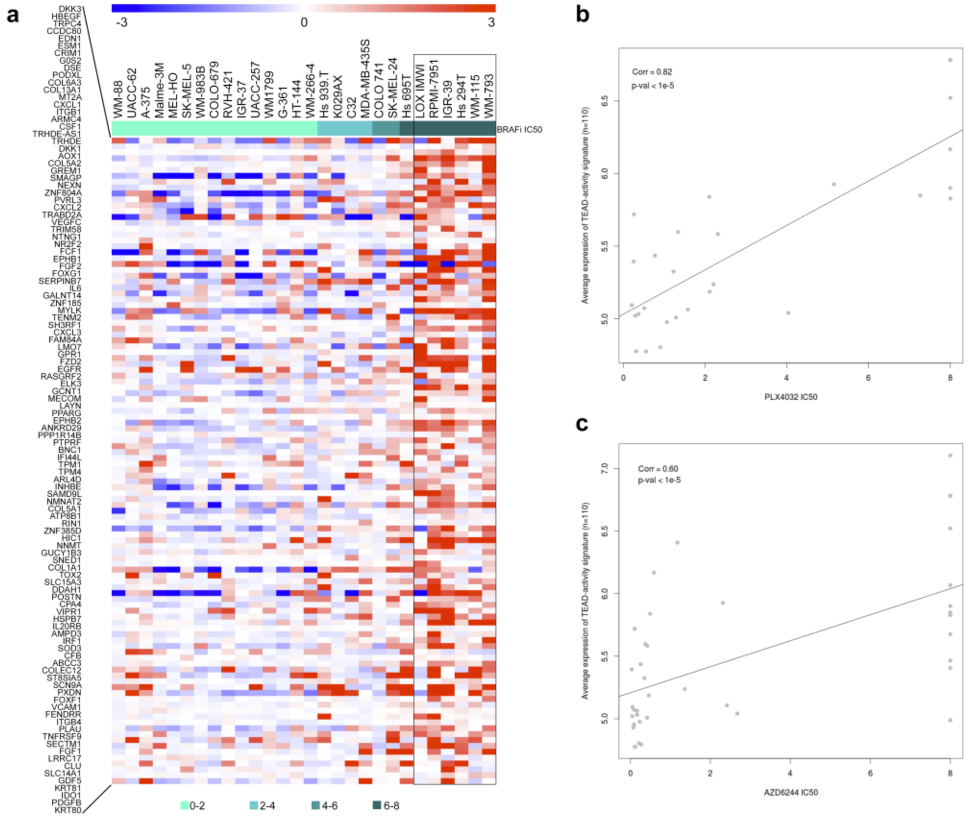

Supplementary Figure 26 – Expression of TEAD-targets correlate with drug resistance in melanoma cell lines. (**a**) Expression heatmap of 110 TEAD target genes (candidate TEAD-targets that have |log2FC|>1 and p-adj<0.05 in TEAD-KD experiment in MM047) across 29 BRAF^{V600E}-mutant cell lines in Cancer Cell Line Encyclopedia. BRAFi IC50 values are color-coded on top of the heatmap with green gradient. In total, we have identified 6 cell lines (shaded in the heatmap) that have high TEAD target expression and resistance to BRAFi (IC50>6). (**b**) There is a significant correlation between the average expression of TEAD-target signature and BRAFi IC50 values for 29 BRAF^{V600E}-mutant cell lines (Pearson's correlation coefficient of 0.82). (**c**) Average expression of TEAD-target signature is plotted against the MEKi IC50 values for 39 melanoma cell lines (29 BRAF^{V600E}-mutant cell lines and 10 BRAF^{WT} cell lines), and this also results in a significant correlation (with Pearson's correlation coefficient of 0.60).

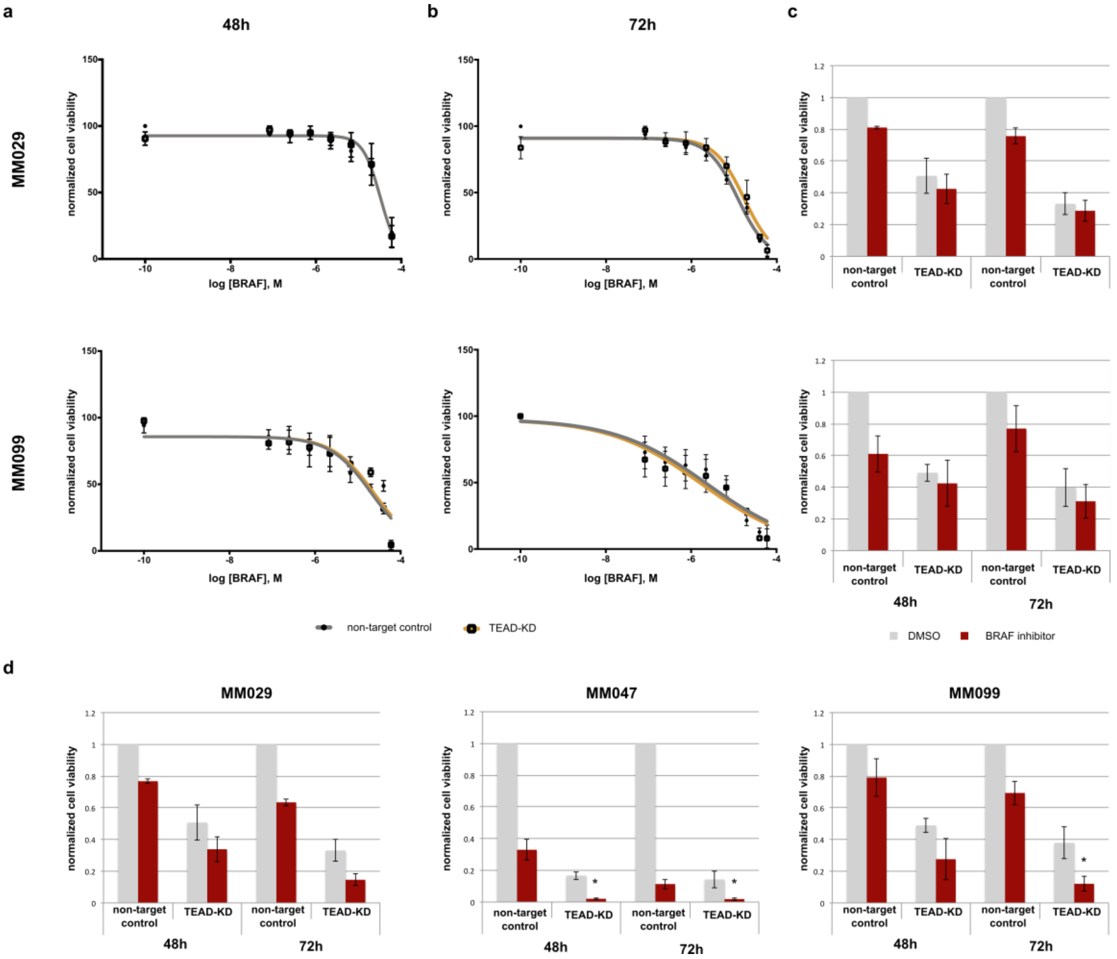

Supplementary Figure 27 - Combined effect of a general TEAD knock down and treatment with different inhibitory drugs on the invasive cell lines. (a) Dose response curves for cells transfected with siRNA against all for TEADs (orange) or non-target control siRNA (grey). Cells were treated with various concentrations of a BRAF inhibitor for 48h. (**b**) Dose response curves for cells transfected with siRNA against all for TEADs (orange) or non-target control siRNA (grey). Cells were treated with various concentrations of a BRAF inhibitor for 72h. (**c**) Bar charts showing the effect of treatment with the BRAF inhibitor, treatment with siRNA against all four TEADs alone or combined. BRAF concentrations were administered at concentrations within each cell lines drug-responsive concentration range. (**d**) Bar charts showing the effect of treatment with the MEK inhibitor, treatment with siRNA against all four TEADs alone or combined. MEK concentrations were administered at concentrations within each cell lines drug-responsive concentration range.

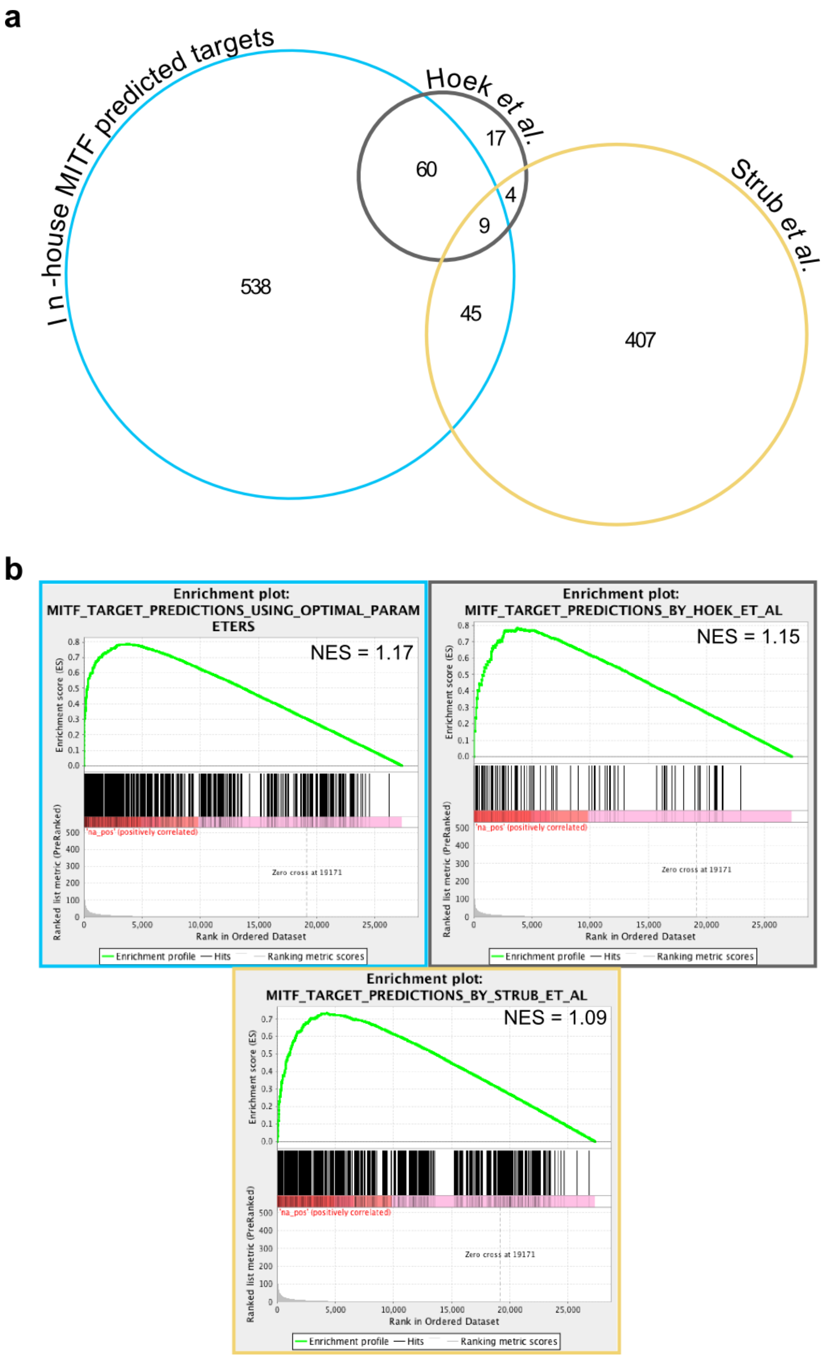

Supplementary Figure 28 - GSEA results for MITF target predictions on the MITF perturbation data. (a) Venn diagram showing the overlapping genes between our predicted MITF target genes, and MITF target gene predictions presented by Hoek *et al*[6] and Strub *et al*[3]. Our own MITF predicted targets have the best overlap with the targets from Hoek. **(b)** MITF target predictions from Hoek *et al*[6], Strub *et al*[3] and from this study were analyzed with GSEA over the MITF perturbation data (x-axis shows ranked genes in the genome upon MITF KD, with strongest down-regulated on the left) [3] . The results revealed that the MITF target predictions that we generate using optimal parameters for region-to-gene mapping have the highest NES among these three prediction sets.

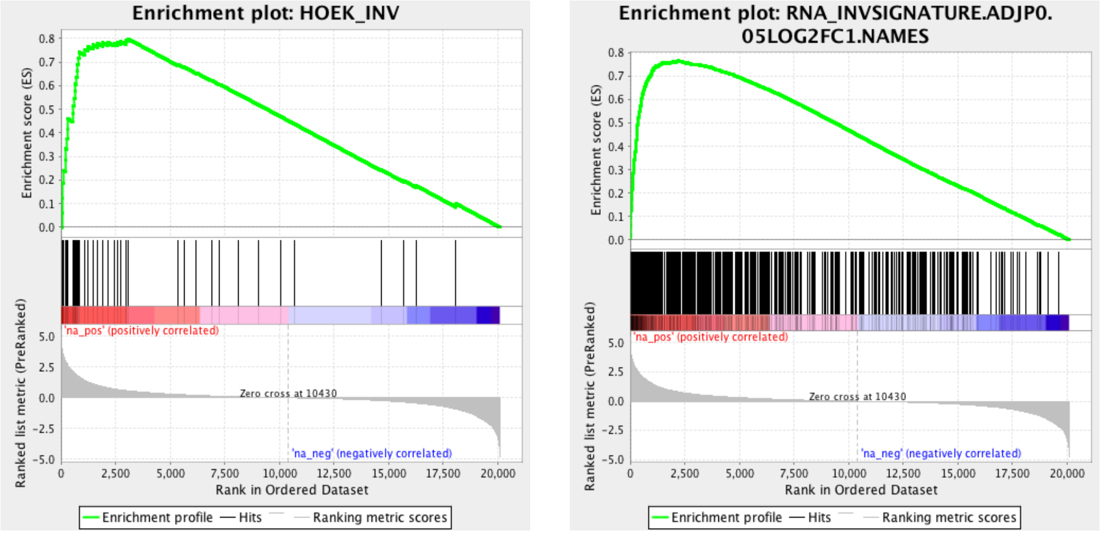

Supplementary Figure 29 – SOX10 KD causes a switch to the invasive state. GSEA of the SOX10 KD dataset[7] against the invasive signature of Hoek et al. with 45 genes (left), and against our new invasive gene signature with 643 genes (right). The gene-rankings (x-axis) were generated by contrasting the SOX10 KD samples against control. Left panel shows a strong enrichment of the invasive Hoek signature (NES = 2.23) amongst the genes that are up-regulated upon SOX10 KD. Similarly, the right panel shows the enrichment of our own invasive signature based on RNA-seq (NES = 2.97) amongst the genes up-regulated upon SOX10 KD.

Supplementary Tables

		Biopsy samples (primary or metastasis)	Culture	Cell Lines	Normal (skin or melanocyte)	Total number of samples	Total number of samples used in analysis
Compendium A	GSE12627	44	-	8	-	52	52
	GSE8401	83	-	-	-	83	83
Compendium B	GSE15605	58	-	-	16	74	58
	GSE19234	44	-	-	-	44	44
	GSE23376	22	-	-	-	22	22
	GSE33728	-	15	-	-	15	15
	GSE38312	-	5	-	5	10	5
	GSE7127	-	5	63	-	63	63
	GSE7553	54	2	-	5	87*	56
SKCM		375				375	375
Total		680	27	71	26		773

*including 26 other metastatic cancer samples

Supplementary Table 1 – Description of the publicly available melanoma gene expression datasets. Compendium A consisted of two datasets generated with Affymetrix Human Genome U133A Arrays totaling to 127 samples, while Compendium B consisted of 7 datasets generated with Affymetrix U133 PLUS 2.0 arrays and totaling 263 samples. SKCM represents a cohort of melanoma samples derived from TCGA.

A. Functional enrichment results with GSEA

Only the enrichments with FDR<0.05 are shown

	Gene Signature	NES	FDR
Gene signatures enriched in the invasive samples	Mesenchymal Transition Signature (PMID:22208948)	1.94	0.00
	Mesenchymal Transition Signature (PMID:14562044)	1.80	0.01
	Extracellular Region (GO)	1.79	0.01
	FRA Pathway (Pathway Interaction Database)	1.73	0.04
	Extracellular Matrix Organization (Reactome)	1.72	0.04
	Focal adhesion (KEGG)	1.71	0.04
	Hoek Invasive Signature (PMID:22336146)	1.98	0.00
Gene signatures enriched in the proliferative samples	Hoek Proliferative Signature (PMID:22336146)	1.98	0.00
	MITF targets (PMID:19067971)	2.11	0.00

B. Functional enrichment results with GOrilla

Top 15 most-significantly enriched GO Terms are shown

	GO Term Description	Enrichment	FDR
GO Terms enriched in the invasive samples	extracellular matrix organization	4.20	8.3E-29
	extracellular structure organization	4.18	6.0E-29
	signal transduction	1.71	7.7E-23
	response to stimulus	1.46	4.2E-22
	biological adhesion	3.16	4.9E-21
	cell adhesion	3.15	1.2E-20
	regulation of cell migration	3.97	1.1E-20
	regulation of locomotion	3.62	3.8E-19
	regulation of cell motility	3.70	1.1E-18
	cell surface receptor signaling pathway	1.95	3.8E-18
	immune system process	2.12	7.0E-18
	response to wounding	4.38	5.3E-17
	response to external stimulus	2.28	5.8E-17
	cellular response to stimulus	1.51	8.3E-17
	regulation of cellular component movement	3.45	7.9E-17
GO Terms enriched in the proliferative samples	melanocyte differentiation	14.93	1.1E-09
	pigment cell differentiation	14.25	1.5E-09
	neuroepithelial cell differentiation	12.06	2.6E-08
	melanin metabolic process	20.95	7.4E-08
	melanin biosynthetic process	26.95	1.3E-07
	ion transport	1.90	2.6E-07
	secondary metabolite biosynthetic process	24.26	5.5E-07
	single-multicellular organism process	1.56	1.1E-06
	columnar/cuboidal epithelial cell differentiation	8.96	1.6E-06
	multicellular organismal process	1.54	1.6E-06
	neurological system process	2.76	3.0E-06
	system process	2.31	4.7E-06
	response to hormone	1.95	1.2E-05
	single-organism transport	1.43	1.3E-05
	response to insulin	2.58	3.4E-05

Supplementary Table 2 – Functional characterization of 11 cultures. Gorilla (**a**) and GSEA (**b**) results on the RNA-seq data of the 11 melanoma cultures showing the various functions enriched in the identified invasive and proliferative samples.

a

Results from hypergeometric test for comparison tissue specific enhancers with predicted melanoma invasive CRRs

# Total number of genes:	1223024
# Total number of unique genes in gmt:	20797
# Total number of unique genes in geneset:	13453
# Number of common genes in geneset and gmt:	13453
# Total number of unique genes in gmt (passing min_overlap and min_genes_cluster filter):	20785
# Number of common genes in geneset and gmt (passing min_overlap and min_genes_cluster filter):	1234
# Minimum number of overlapping genes in cluster:	3
# Minimum number of genes in cluster:	3
#	

# cluster_id	cluster size	# overlap	representation factor	p-value	adjusted_p-value
CL:0000359_vascular_associated_smooth_muscle_cell_differentially_expressed_enhancers.bed	2120	387	16.596	2.60E-333	2.60E-331
**CL:0002620_skin_fibroblast_differentially_expressed_enhancers.bed**	1406	286	18.493	1.26E-260	1.26E-258
CL:0000071_blood_vessel_endothelial_cell_differentially_expressed_enhancers.bed	1982	255	11.696	1.52E-180	1.52E-178
**CL:0000134_mesenchymal_cell_differentially_expressed_enhancers.bed**	2308	242	9.532	1.45E-150	1.45E-148
CL:0002368_respiratory_epithelial_cell_differentially_expressed_enhancers.bed	1300	180	12.588	1.51E-133	1.51E-131
CL:0002518_kidney_epithelial_cell_differentially_expressed_enhancers.bed	992	159	14.571	2.40E-128	2.40E-126
CL:0000499_stromal_cell_differentially_expressed_enhancers.bed	868	141	14.768	7.70E-115	7.70E-113
CL:0002334_preadipocyte_differentially_expressed_enhancers.bed	829	135	14.805	3.57E-110	3.57E-108
CL:0000188_skeletal_muscle_cell_differentially_expressed_enhancers.bed	976	138	12.854	5.10E-104	5.10E-102
CL:0000056_myoblast_differentially_expressed_enhancers.bed	616	114	16.824	7.55E-100	7.55E-98
CL:0000127_astrocyte_differentially_expressed_enhancers.bed	846	126	13.540	5.62E-98	5.62E-96
CL:0000062_osteoblast_differentially_expressed_enhancers.bed	580	108	16.928	5.51E-95	5.51E-93
CL:0002556_fibroblast_of_periodontium_differentially_expressed_enhancers.bed	516	102	17.971	1.37E-92	1.37E-90
CL:0002552_fibroblast_of_gingiva_differentially_expressed_enhancers.bed	490	97	17.997	3.38E-88	3.38E-86
CL:0002621_gingival_epithelial_cell_differentially_expressed_enhancers.bed	660	107	14.739	1.83E-87	1.83E-85
CL:0002559_hair_follicle_cell_differentially_expressed_enhancers.bed	443	93	19.085	3.74E-87	3.74E-85
CL:0000576_monocyte_differentially_expressed_enhancers.bed	8701	339	3.542	2.13E-85	2.13E-83
CL:0002548_cardiac_fibroblast_differentially_expressed_enhancers.bed	546	89	14.819	2.96E-73	2.96E-71
CL:0000731_urothelial_cell_differentially_expressed_enhancers.bed	426	80	17.072	4.09E-71	4.09E-69
CL:0000098_sensory_epithelial_cell_differentially_expressed_enhancers.bed	833	102	11.132	4.91E-71	4.91E-69
CL:1000306_fibroblast_of_tunica_adventitia_of_artery_differentially_expressed_enhancers.bed	335	73	19.810	4.78E-70	4.78E-68
CL:0002224_lens_epithelial_cell_differentially_expressed_enhancers.bed	305	63	18.778	4.06E-59	4.06E-57
CL:0000067_ciliated_epithelial_cell_differentially_expressed_enhancers.bed	488	74	13.786	7.71E-59	7.71E-57
CL:0000136_fat_cell_differentially_expressed_enhancers.bed	673	83	11.212	2.22E-58	2.22E-56
CL:0002504_enteric_smooth_muscle_cell_differentially_expressed_enhancers.bed	353	63	16.225	6.49E-55	6.49E-53
CL:0000312_keratinocyte_differentially_expressed_enhancers.bed	627	76	11.019	4.79E-53	4.79E-51
CL:0002554_fibroblast_of_lymphatic_vessel_differentially_expressed_enhancers.bed	267	55	18.727	9.33E-52	9.33E-50
CL:0000077_mesothelial_cell_differentially_expressed_enhancers.bed	377	61	14.710	1.50E-50	1.50E-48
CL:0002327_mammary_epithelial_cell_differentially_expressed_enhancers.bed	437	64	13.314	3.80E-50	3.80E-48
CL:0002577_placental_epithelial_cell_differentially_expressed_enhancers.bed	461	64	12.621	1.16E-48	1.16E-46
CL:0002166_epithelial_cell_of_Malassez_differentially_expressed_enhancers.bed	371	58	14.212	2.88E-47	2.88E-45
CL:0000138_chondrocyte_differentially_expressed_enhancers.bed	364	56	13.986	2.67E-45	2.67E-43
CL:0000451_dendritic_cell_differentially_expressed_enhancers.bed	3753	152	3.682	4.32E-41	4.32E-39
CL:1000398_endothelial_cell_of_hepatic_sinusoid_differentially_expressed_enhancers.bed	202	41	18.452	1.00E-38	1.00E-36
CL:0002586_retinal_pigment_epithelial_cell_differentially_expressed_enhancers.bed	262	42	14.573	3.71E-35	3.71E-33
CL:0002536_amniotic_epithelial_cell_differentially_expressed_enhancers.bed	302	44	13.245	6.61E-35	6.61E-33
CL:0000388_tendon_cell_differentially_expressed_enhancers.bed	303	44	13.202	7.65E-35	7.65E-33
CL:0002231_epithelial_cell_of_prostate_differentially_expressed_enhancers.bed	366	46	11.426	1.72E-33	1.72E-31
CL:0000767_basophil_differentially_expressed_enhancers.bed	1200	75	5.682	1.43E-32	1.43E-30
CL:0002601_uterine_smooth_muscle_cell_differentially_expressed_enhancers.bed	202	36	16.202	4.52E-32	4.52E-30
CL:0002598_bronchial_smooth_muscle_cell_differentially_expressed_enhancers.bed	147	31	19.172	3.19E-30	3.19E-28
CL:0000622_acinar_cell_differentially_expressed_enhancers.bed	369	43	10.594	4.83E-30	4.83E-28
CL:1000487_smooth_muscle_cell_of_prostate_differentially_expressed_enhancers.bed	244	36	13.413	4.66E-29	4.66E-27
CL:0002367_trabecular_meshwork_cell_differentially_expressed_enhancers.bed	224	34	13.799	5.90E-28	5.90E-26
CL:0000746_cardiac_myocyte_differentially_expressed_enhancers.bed	177	29	14.895	4.66E-25	4.66E-23
CL:0002252_epithelial_cell_of_esophagus_differentially_expressed_enhancers.bed	193	28	13.189	9.40E-23	9.40E-21
CL:0002565_iris_pigment_epithelial_cell_differentially_expressed_enhancers.bed	114	23	18.342	2.34E-22	2.34E-20
CL:0000575_corneal_epithelial_cell_differentially_expressed_enhancers.bed	213	28	11.951	1.46E-21	1.46E-19
CL:0002549_fibroblast_of_choroid_plexus_differentially_expressed_enhancers.bed	224	28	11.364	5.82E-21	5.82E-19
CL:0000632_hepatic_stellate_cell_differentially_expressed_enhancers.bed	211	26	11.202	2.18E-19	2.18E-17
CL:0000775_neutrophil_differentially_expressed_enhancers.bed	2194	79	3.273	3.65E-19	3.65E-17
UBERON:0002048_lung_differentially_expressed_enhancers.bed	445	34	6.946	3.10E-18	3.10E-16
CL:0002138_endothelial_cell_of_lymphatic_vessel_differentially_expressed_enhancers.bed	332	29	7.941	2.54E-17	2.54E-15
CL:0000084_T_cell_differentially_expressed_enhancers.bed	4360	116	2.419	3.47E-17	3.47E-15
UBERON:0000995_uterus_differentially_expressed_enhancers.bed	173	21	11.035	8.11E-16	8.11E-14
CL:0000097_mast_cell_differentially_expressed_enhancers.bed	3313	93	2.552	1.94E-15	1.94E-13
CL:0002557_fibroblast_of_pulmonary_artery_differentially_expressed_enhancers.bed	125	18	13.091	4.31E-15	4.31E-13
UBERON:0000178_blood_differentially_expressed_enhancers.bed	1432	55	3.492	6.19E-15	6.19E-13
CL:0002600_smooth_muscle_cell_of_trachea_differentially_expressed_enhancers.bed	110	17	14.050	7.33E-15	7.33E-13
CL:0002550_fibroblast_of_the_conjuctiva_differentially_expressed_enhancers.bed	127	17	12.169	8.49E-14	8.49E-12
CL:0002599_smooth_muscle_cell_of_the_esophagus_differentially_expressed_enhancers.bed	99	14	12.856	5.98E-12	5.98E-10
CL:0000623_natural_killer_cell_differentially_expressed_enhancers.bed	3121	80	2.330	1.40E-11	1.40E-09
UBERON:0002113_kidney_differentially_expressed_enhancers.bed	200	18	8.182	1.53E-11	1.53E-09
UBERON:0001043_esophagus_differentially_expressed_enhancers.bed	146	15	9.340	1.14E-10	1.14E-08
CL:0000669_pericyte_cell_differentially_expressed_enhancers.bed	269	19	6.421	2.78E-10	2.78E-08
CL:0000235_macrophage_differentially_expressed_enhancers.bed	1513	46	2.764	1.81E-09	1.81E-07
UBERON:0001723_tongue_differentially_expressed_enhancers.bed	151	14	8.429	1.82E-09	1.82E-07
CL:0002363_keratocyte_differentially_expressed_enhancers.bed	94	11	10.639	8.49E-09	8.49E-07
UBERON:0001013_adipose_tissue_differentially_expressed_enhancers.bed	124	10	7.332	1.35002E-06	0.000135002
UBERON:0002367_prostate_gland_differentially_expressed_enhancers.bed	127	10	7.158	1.67932E-06	0.000167932
UBERON:0000992_female_gonad_differentially_expressed_enhancers.bed	101	9	8.101	1.97586E-06	0.000197586
UBERON:0002110_gallbladder_differentially_expressed_enhancers.bed	93	8	7.820	9.49649E-06	0.000949649
UBERON:0001255_urinary_bladder_differentially_expressed_enhancers.bed	123	9	6.652	1.00291E-05	0.001002908
UBERON:0002106_spleen_differentially_expressed_enhancers.bed	307	13	3.850	4.61231E-05	0.004612311
UBERON:0001987_placenta_differentially_expressed_enhancers.bed	96	7	6.629	9.86959E-05	0.009869593
UBERON:0001134_skeletal_muscle_tissue_differentially_expressed_enhancers.bed	99	7	6.428	0.000119799	0.011979944
UBERON:0000996_vagina_differentially_expressed_enhancers.bed	73	6	7.472	0.000160404	0.016040371
UBERON:0002360_meninx_differentially_expressed_enhancers.bed	107	7	5.947	0.000194464	0.019446366

b

Results from hypergeometric test for comparison tissue specific enhancers with predicted melanoma proliferative CRRs

# Total number of genes:	1223024
# Total number of unique genes in gmt:	20797
# Total number of unique genes in geneset:	6669
# Number of common genes in geneset and gmt:	6669
# Total number of unique genes in gmt (passing min_overlap and min_genes_cluster filter):	19504
# Number of common genes in geneset and gmt (passing min_overlap and min_genes_cluster filte	298
# Minimum number of overlapping genes in cluster:	3
# Minimum number of genes in cluster:	3
#	

# cluster id	cluster size	# overlap	representation factor	p-value	adjusted p-value
CL:0000148_melanocyte_differentially_expressed_enhancers.bed	500	85	31.176	1.20E-96	4.79E-95
CL:0000576_monocyte_differentially_expressed_enhancers.bed	8701	138	2.909	2.51E-27	1.00E-25
CL:0000097_mast_cell_differentially_expressed_enhancers.bed	3313	69	3.819	2.47E-20	9.89E-19
UBERON:0000955_brain_differentially_expressed_enhancers.bed	642	27	7.713	9.82E-16	3.93E-14
CL:0000235_macrophage_differentially_expressed_enhancers.bed	1513	40	4.848	1.22E-15	4.87E-14
CL:0000451_dendritic_cell_differentially_expressed_enhancers.bed	3753	57	2.785	1.99E-11	7.95E-10
CL:0000623_natural_killer_cell_differentially_expressed_enhancers.bed	3121	40	2.350	1.28E-06	5.14E-05
CL:0000945_lymphocyte_of_B_lineage_differentially_expressed_enhancers.bed	1266	21	3.042	1.08E-05	4.33E-04
CL:0000084_T_cell_differentially_expressed_enhancers.bed	4360	45	1.893	6.19E-05	2.47E-03
UBERON:0002370_thymus_differentially_expressed_enhancers.bed	365	8	4.019	1.02E-03	4.06E-02

Supplementary Table 3 – Tissue-specific enhancers from Andersson *et al* [8] that significantly overlap with the invasive (a) and proliferative (b) candidate regulatory regions. Overlap statistics are calculated using the hypergeometric statistic.

	GBP[9]	CpG	Proximal promoters	CNS	UCR	OReganno[10]	Vista Enhancers[11]	CRMs[12]	DHS[13]
# regions	61550	27718	34722	232101	15931	23112	1339	123500	1281988
% of the genome	1.77	0.73	0.67	2.25	0.13	0.39	0.07	2.05	13.36

Supplementary Table 4 – Publicly available data used for generating candidate regulatory regions. Different sources were used to define a total of 1,223,024 candidate regulatory regions.

	Sequencing										
	RNA										
	# RAW READS	# CLEAN READS	# UNMAPPED READS*	# MAPPED READS* (unique read names)	% MAPPED READS* (unique read names) of CLEAN READS	# MAPPED READS*	# MAPPED READS* Properly paired	% MAPPED READS* Properly paired	# MAPPED READS* without DUPLICATES	# MAPPED READS* without DUPLICATES Properly paired	% MAPPED READS* without DUPLICATES Properly paired
MM001	93822066	90980960	7520908	83460052	91.73%	94771359	67990710	71.74%	78140404	56622892	72.46%
MM011	103316636	100270594	6236765	94033829	93.78%	106038783	76359718	72.01%	86457192	61976094	71.68%
MM031	79011534	76180436	7294421	68886015	90.42%	80849809	55132052	68.19%	66316611	45402406	68.46%
MM034	160543040	154348024	19778040	134569984	87.19%	166313574	129291836	77.74%	129183994	102241540	79.14%
MM047	91059680	86053680	11011347	75042333	87.20%	86772527	62882148	72.47%	72976473	54920784	75.26%
MM057	93178250	90086804	7116606	82970198	92.10%	94326356	64918130	68.82%	75981969	52121586	68.60%
MM074	95763358	92784540	7521390	85263150	91.89%	98229952	73361056	74.68%	80274378	60515572	75.39%
MM087	73471020	71298154	5558251	65739903	92.20%	73277230	50963846	69.55%	62819496	44217190	70.39%
MM099	93524616	88425212	11819670	76605542	86.63%	88175315	65353482	74.12%	72824060	56141666	77.09%
MM118	83764848	77922408	11061342	66861066	85.80%	84107579	61170088	72.73%	67945790	50732168	74.67%
SKMEL	104626208	99847642	12232501	87615141	87.75%	105681493	80501398	76.17%	86196996	67740596	78.59%
	TEAD KD RNA-seq on MM047										
	# RAW READS	# CLEAN READS	# UNMAPPED READS*	# MAPPED READS* (unique read names)	% MAPPED READS* (unique read names) of CLEAN READS	# MAPPED READS*	# MAPPED READS* Properly paired	% MAPPED READS* Properly paired	# MAPPED READS* without DUPLICATES	# MAPPED READS* without DUPLICATES Properly paired	% MAPPED READS* without DUPLICATES Properly paired
KD1	31003964	27844266	1575659	26268607	0.94341172	15141544	NA	NA	NA	NA	NA
KD2	28399714	25094382	591424	24502958	0.97643202	14867351	NA	NA	NA	NA	NA
KD3	14869388	14764698	662604	14102094	0.95512241	8216180	NA	NA	NA	NA	NA
Control1	39531842	38924342	814999	38109343	0.97906197	23092542	NA	NA	NA	NA	NA
Control2	35269276	34452900	704966	33747934	0.97953827	20540360	NA	NA	NA	NA	NA
Control3	15180362	15094890	657527	14437363	0.95644042	8432544	NA	NA	NA	NA	NA

*numbers of reads for data where the first 1 bp were removed from each read

Supplementary Table 5 – Sequencing and mapping statistics of RNA-seq data analyses. Overview of the number of reads after each step in the analysis of RNA-seq data of each of the 11 melanoma samples and for the biological replicates of the TEAD KD in MM047. The analysis pipeline starts with the number of raw reads after sequencing (first column) and ends with the number of cleaned, mapped, properly paired reads, where all duplicates are removed (last column).

4C#primers	fwd	rev
viewpoint#1	GACATTCAAGATACAAATC	CAAGCCTTTTTATTCCCCCT
viewpoint2	TGGAGGATGATGGAGAATCG	AAACACAAATGTCCAAAGGGA
	TAAGAAATTCTCCCGGAAGGA	GCTGAGATTTATATTTGCAG

qPCR#primers	fwd	rev
SOX9	GCAAGCTCTGGAGACTTCTG	GTACTTGTAATCCGGGTGGTC
EPHA2	GGAGGGATCTGGCAACTTGG	CTTCCTCCTGCGGTGGATAA
SERPINE1	GCTTTTGTGTGCCTGGTAGAAA	TGGCAGGCAGTACAAGAGTGA
TEAD1	CTTGAATGTGCAATGAAGCG	CGAAGTTTGCCTCGGACTC
TEAD2	CTCACTCCGTAGAAGCCACC	TGCCTTCTTCCTGGTCAAGT
TEAD3	GCACCTTCTTCCGAGCTAGA	TACGGCCGAAATGAGTTGAT+
TEAD4	GCTCCACTCGTTGGAGGTAA	CTTAGCGCACCCATCCC
SOX10	CCAGTACCCGCACCTGCAC	CTTTCGTTCAGCAGCCTCCAG
MITF	CATTGTTATGCTGGAAATGCTAGAA	GGCTTGCTGTATGTGGTACTTGG
EGFR	TGCACCTACGGATGCACTG	CGATGGACGGGATCTTAGGC
UBC	ATTTGGGTCGCGGTTCTTG	TGCCTTGACATTCTCGATGGT
TBP	CGGCTGTTTAACTTCGCTTC	CACACGCCAAGAAACAGTGA
YWHAZ	ACTTTTGGTACATTGTGGCTTCAA	CCGCCAGGACAAACCAGTAT

siRNAs	
TEAD#1/3/4	UGAUCAACUUCAUCCACAA
TEAD#2/4	GCAAGCAGGUGGUGGAGAA
TEAD2	CGAAGGAAAUCAAGGGAAA

Supplementary Table 6 – siRNAs and primer sequences. Forward and reverse primer sequences used in qPCR and the sequences of the siRNAs aimed against the different TEADs. TEAD 1/3/4 indicates that the siRNA is directed against TEAD1, TEAD3 and TEAD4. TEAD 2/4 is directed against TEAD2 and TEAD4. TEAD2 siRNA was used for targeting TEAD2 specifically.

	Sequencing														
	FAIRE					H3K27ac					H3K27me3				
	# RAW READS	# CLEAN READS**	# MAPPED READS*	# MAPPED READS (minMQ4)*	# MAPPED READS (minMQ4) without DUPLICATES*	# RAW READS	# CLEAN READS**	# MAPPED READS*	# MAPPED READS (minMQ4)*	# MAPPED READS (minMQ4) without DUPLICATES*	# RAW READS	# CLEAN READS**	# MAPPED READS*	# MAPPED READS (minMQ4)*	# MAPPED READS (minMQ4) without DUPLICATES*
**MM001**	67289408	63465652	62882385	54786506	52665315	48462782	42934171	41893228	37643136	25134160	69049005	68363311	67049982	57009717	16925341
**MM011**	75253802	70169169	69580540	60148237	57935783	52970463	35356022	34386043	31093752	22539992	36072080	35861934	35224780	30705380	6343387
**MM031**	42414818	41336220	41038055	36224489	35287269	60451940	38912647	37753355	33260082	24827544	57642203	41918685	38359442	32149446	21172175
**MM034**	76419768	73307133	72743525	63814475	56781142	44126961	30947016	29125972	26881036	6854609	47225284	27358725	26119945	22352342	19136338
**MM047**	69301467	68592347	68111835	60186464	58380264	37271404	35713446	35359381	31265129	26310659	37568881	34749585	32599883	28087225	15191247
**MM057**	105009641	103615601	102790003	90770914	87105049	51994847	48702333	47937974	43384573	18421497	51147715	49369723	48371555	40912749	9206636
**MM074**	92755234	81744247	80911105	69815144	67463718	48220305	35266499	28865060	26623504	8218885	41814610	27730607	26307348	22545671	19030624
**MM087**	63874389	62156806	61639767	53571138	51875191	34740785	32929151	32474545	28331557	24808835	62181559	59479385	58312624	48478182	15588288
**MM099**	87250082	85803470	85155726	75544575	73015661	39326298	33142192	31865756	28891762	20316940	50462758	38528986	35411805	29929722	22889946
**MM118**	80738583	79326504	78666561	69443298	66053890	43489615	36407147	35049562	30960315	27180978	52790663	46508417	45654304	39197701	25972444
**SKMEL**	74613691	72877110	72261046	62894814	60511591	54601467	42115332	40958353	35986788	30396641	46638037	34508176	33104831	28284455	24584858

	TFBS_ChIP				
ChIP	# RAW READS	# CLEAN READS**	# MAPPED READS*	# MAPPED READS (minMQ4)*	# peaks
**ChIP_MITF_MM011**	74760962	73595953	71902685	61894857	3907
**ChIP_MITF_MM031**	18865678	13426544	13079978	11343375	810

	Input			
	# RAW READS	# CLEAN READS**	# MAPPED READS*	# MAPPED READS (minMQ4)*
**Input_MM057**	39734807	35795094	34779690	30816803
**Input_MM011**	12421636	10151978	10026070	8608944
**Input_MM047**	16753245	16007186	15827695	13736901

*numbers of reads for data where the first 5 bp were removed from each read
**numbers of reads for data with and without the first 5 bp – numbers are the same

Supplementary Table 7 – Sequencing and mapping statistics concerning regulatory data analyses. Overview of the number of reads after each step in the analysis of all regulatory data. The analysis pipeline starts with the number of raw reads after sequencing (first column) and ends with the number of cleaned, mapped reads (columns 4). Additionally, for FAIRE-seq (blue), H3K27ac (green) and H3K27me3 (orange) an additional column indicates the number of reads after duplicate removal. Similarly, for the two ChIP-seq data sets (light blue) the number of peaks called with MACS2 is shown in column 5. Finally, the statistics for three different input samples are given (grey), which were then merged and used as one input file.

Supplementary Notes

Supplementary Note 1 – Mutations and copy number aberrations in relation to cell states

To test the association between the mutation status and cell states we focused on 116 genes, which are significantly mutated according to Firehose analysis (analyses__2014_04_16), and three cell states (invasive, proliferative and immune-infiltrated). There were 343 samples that had both a cluster assignment as a result of the NMF analysis and somatic mutation calls, thus these samples were used for analysis. Two-tailed Fisher's exact test [14] was used to estimate p-values using the '*fisher.test*' function in R, and Q-values were calculated with Benjamini & Hochberg method [15] to correct for multiple hypothesis correction.
At Q-value threshold of 0.05, no significant association was detected (see **Table 1**). With Q-value threshold of 0.25 and p-value threshold of 0.05 mutation status of two genes were associated significantly with cell states: THEMIS (**Figure 1**) and GNAI2 (**Figure 2**).

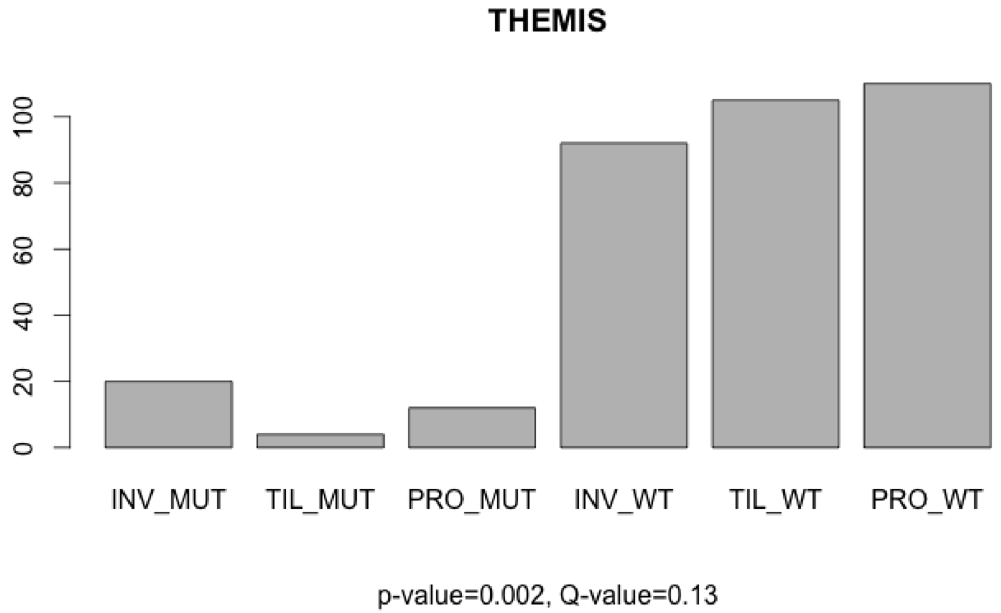

Figure 1. Mutation frequency plot for THEMIS gene showing the number of mutated and wild-type samples in three cell states.

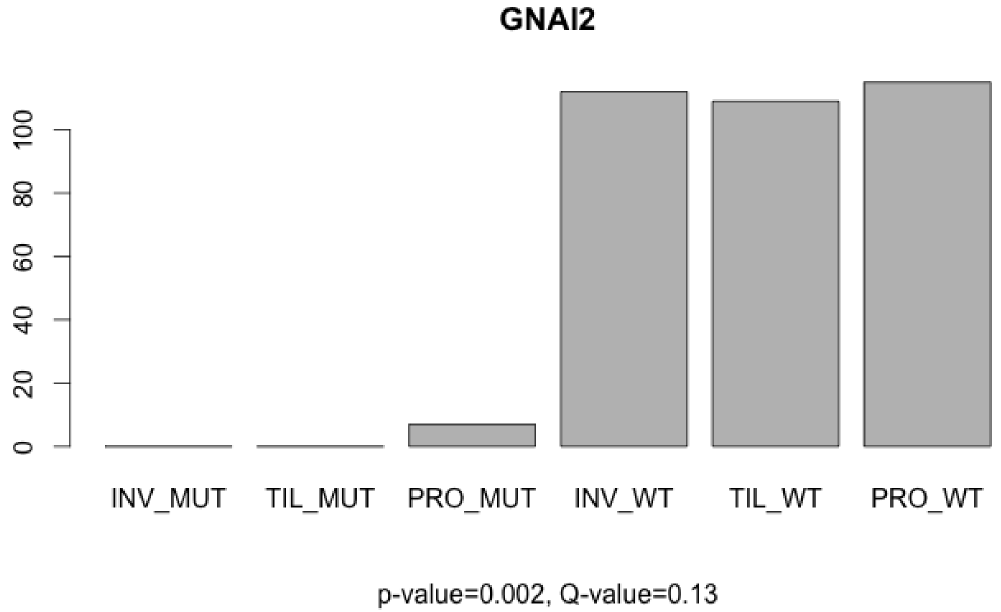

Figure 2. Mutation frequency plot for GNAI2 gene showing the number of mutated and wild-type samples in three cell states.

Note that we also checked the existence of these mutations in our short-term cultures and detected no protein-altering GNAI2 mutations nor THEMIS mutations.

We also investigated a second type of aberration, namely copy number alterations (CNAs). There were 290 samples that had both a cell state assignment as a result of the NMF analysis and copy number aberrations (54), thus these samples were used for analysis. We analyzed whether any specific CNA is specifically enriched in any of the three cell states, and found only one significant correlation (at the threshold of Q<0.05) (**Table 2**), namely amplification of 7q34 locus, which is observed in 39 samples in total (**Figure 3**). This locus contains 89 genes including BRAF. Amplification of BRAF is one of the ways that melanomas re-activate MAPK pathway as a therapy resistance mechanism [16]. For 34 out of 39 samples, clinical information is available and reveals that all the samples that carry this amplification is a metastatic sample, leading to the possibility that this observed genomic aberration might stem from therapy resistance.

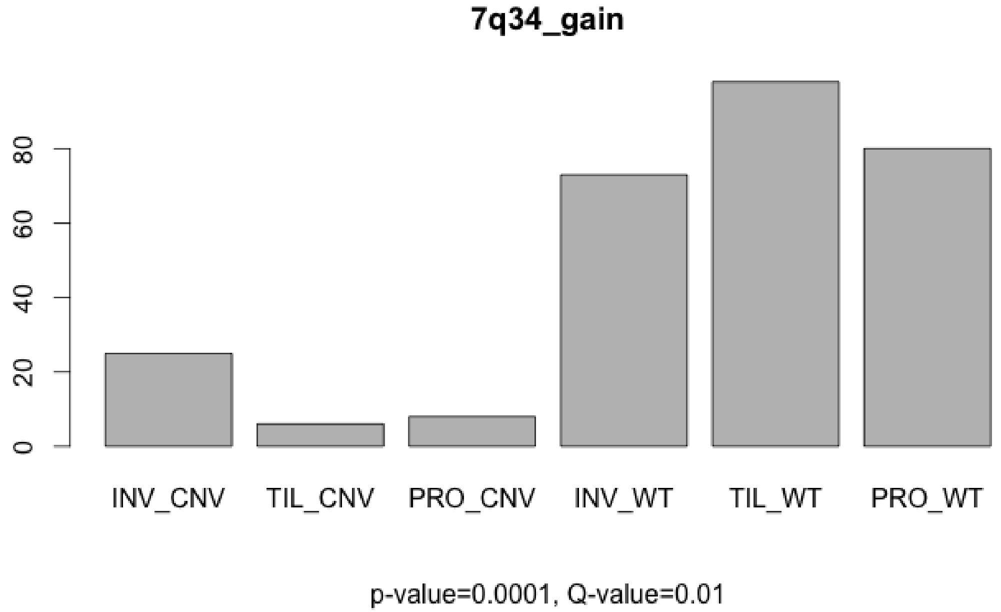

Figure 3. CNA frequency plot for 7q34 amplification showing the number of copy-number aberrant and wild-type samples in three cell states.

Table 1. Overview of the mutation status of 116 genes in relation to three cell states

	Invasive MUT	Immune-infiltrated MUT	Proliferative MUT	Invasive WT	Immune-infiltrated WT	Proliferative WT	Fisher's test p-value	Fisher's test q-value
THEMIS	20	4	12	92	105	110	0.002	0.130
GNAI2	0	0	7	112	109	115	0.001	0.130
TSHB	0	5	1	112	104	121	0.017	0.640
PROL1	8	13	4	104	96	118	0.041	0.784
NMNAT3	0	5	6	112	104	116	0.036	0.784
NOP10	0	3	0	112	106	122	0.031	0.784
LUZP2	13	4	13	99	105	109	0.060	0.885
C15orf23	11	4	4	101	105	118	0.076	0.885
OR4K1	6	14	16	106	95	106	0.082	0.885
PCDP1	6	3	12	106	106	110	0.084	0.885
MS4A2	1	7	4	111	102	118	0.076	0.885
C1QTNF9	7	9	3	105	100	119	0.124	0.956
CYP4Z1	5	10	14	107	99	108	0.127	0.956
ANKRD20A4	4	0	2	108	109	120	0.125	0.956
TMEM216	1	1	6	111	108	116	0.126	0.956
TBC1D3B	0	4	3	112	105	119	0.132	0.956
PREX2	29	19	28	83	90	94	0.305	0.995
TP53	20	20	13	92	89	109	0.183	0.995
CDKN2A	18	16	11	94	93	111	0.214	0.995
ITGA4	17	11	11	95	98	111	0.311	0.995
C7orf58	15	13	9	97	96	113	0.288	0.995
FUT9	15	7	11	97	102	111	0.218	0.995
FAM113B	14	6	10	98	103	112	0.184	0.995
SYCP1	14	9	8	98	100	114	0.303	0.995
OR2W1	13	6	7	99	103	115	0.184	0.995
LIPI	12	6	9	100	103	113	0.360	0.995
GLYATL2	11	5	12	101	104	110	0.246	0.995
RPS27	11	6	13	101	103	109	0.340	0.995
SIRPB1	10	4	10	102	105	112	0.246	0.995
LPAR1	8	3	6	104	106	116	0.313	0.995
DNAJC5B	7	6	2	105	103	120	0.148	0.995
GPR141	6	8	3	106	101	119	0.216	0.995
SLC10A2	6	9	13	106	100	109	0.326	0.995
BAGE	5	1	4	107	108	118	0.287	0.995
PENK	5	9	11	107	100	111	0.348	0.995
PPIAL4G	5	7	3	107	102	119	0.319	0.995
SLC38A1	4	8	11	108	101	111	0.216	0.995

GALNTL5	3	4	9	109	105	113	0.234	0.995
IDH1	3	6	9	109	103	113	0.262	0.995
SPANXN5	3	4	1	109	105	121	0.326	0.995
CDK4	1	4	2	111	105	120	0.355	0.995
FAM58A	1	4	1	111	105	121	0.291	0.995
PCDHAC2	62	58	63	50	51	59	0.849	1.000
PCDHGC5	59	53	54	53	56	68	0.441	1.000
BRAF	57	56	64	55	53	58	0.972	1.000
NRAS	34	30	30	78	79	92	0.610	1.000
PTPRT	33	27	35	79	82	87	0.698	1.000
COL4A4	24	26	30	88	83	92	0.851	1.000
STXBP5L	24	17	24	88	92	98	0.511	1.000
CRB1	23	17	22	89	92	100	0.647	1.000
KIAA2022	22	18	20	90	91	102	0.780	1.000
ARMC4	21	19	20	91	90	102	0.906	1.000
SCN5A	20	26	28	92	83	94	0.506	1.000
SNCAIP	20	20	18	92	89	104	0.737	1.000
HRNR	18	22	26	94	87	96	0.565	1.000
DSG1	17	15	19	95	94	103	0.927	1.000
STK31	17	12	13	95	97	109	0.525	1.000
TRHDE	17	16	18	95	93	104	1.000	1.000
SERPINB3	16	14	17	96	95	105	0.961	1.000
TCHHL1	16	15	15	96	94	107	0.921	1.000
TMC5	16	16	19	96	93	103	0.981	1.000
LRRC4C	14	15	16	98	94	106	0.979	1.000
PDE1A	14	15	12	98	94	110	0.636	1.000
CHGB	13	11	12	99	98	110	0.904	1.000
COL19A1	13	13	13	99	96	109	0.954	1.000
ZNF99	13	17	17	99	92	105	0.709	1.000
AOAH	12	7	8	100	102	114	0.425	1.000
LRTM1	12	9	9	100	100	113	0.679	1.000
NF1	12	18	17	100	91	105	0.454	1.000
OGDHL	12	10	17	100	99	105	0.515	1.000
ST6GAL2	12	16	16	100	93	106	0.696	1.000
CYP3A7	11	9	10	101	100	112	0.914	1.000
MPP7	11	11	13	101	98	109	1.000	1.000
SLC15A2	11	14	13	101	95	109	0.785	1.000
TPTE2	11	12	15	101	97	107	0.825	1.000
PCSK1	10	7	12	102	102	110	0.671	1.000
POTEG	10	10	15	102	99	107	0.695	1.000
TCEB3C	10	11	9	102	98	113	0.766	1.000

ANGPT1	9	12	12	103	97	110	0.740	1.000
C8orf34	9	12	15	103	97	107	0.558	1.000
CD2	9	6	9	103	103	113	0.750	1.000
GCOM1	9	9	9	103	100	113	0.968	1.000
OR4E2	9	6	12	103	103	110	0.487	1.000
OR51S1	9	14	11	103	95	111	0.462	1.000
PTEN	9	10	11	103	99	111	0.943	1.000
RPGRIP1	9	8	10	103	101	112	1.000	1.000
TMPRSS11B	9	9	8	103	100	114	0.874	1.000
CAPZA3	8	11	11	104	98	111	0.721	1.000
PPP6C	8	8	10	104	101	112	0.967	1.000
C18orf26	7	9	5	105	100	117	0.420	1.000
LOC649330	7	11	12	105	98	110	0.504	1.000
CTNNB1	6	5	6	106	104	116	1.000	1.000
CYP4X1	6	8	9	106	101	113	0.829	1.000
TC2N	6	7	8	106	102	114	0.922	1.000
TUBAL3	6	3	3	106	106	119	0.504	1.000
FASLG	5	3	4	107	106	118	0.816	1.000
MRPS31	5	8	8	107	101	114	0.664	1.000
NDUFB9	5	3	2	107	106	120	0.438	1.000
RAC1	5	7	8	107	102	114	0.775	1.000
RCAN2	5	6	5	107	103	117	0.901	1.000
ACD	4	5	5	108	104	117	0.943	1.000
EMG1	4	3	4	108	106	118	1.000	1.000
NMS	4	5	4	108	104	118	0.882	1.000
STK19	4	6	4	108	103	118	0.703	1.000
CTAGE6P	3	1	4	109	108	118	0.547	1.000
LCE1B	3	6	5	109	103	117	0.585	1.000
OXA1L	3	4	3	109	105	119	0.852	1.000
RERG	3	4	6	109	105	116	0.684	1.000
STARD6	3	4	5	109	105	117	0.874	1.000
APCS	2	5	6	110	104	116	0.406	1.000
ELF5	2	3	5	110	106	117	0.617	1.000
PRSS48	2	1	3	110	108	119	0.875	1.000
RQCD1	2	5	3	110	104	119	0.478	1.000
AREG	1	3	3	111	106	119	0.638	1.000
EIF3D	1	1	3	111	108	119	0.627	1.000
VSNL1	1	2	1	111	107	121	0.695	1.000

Table 2. Overview of the 54 copy number aberrations in relation to three cell states

	Invasive MUT	Immune-infiltrated MUT	Proliferative MUT	Invasive WT	Immune-infiltrated WT	Proliferative WT	Fisher's test p-value	Fisher's test q-value
7q34_gain	25	6	8	73	98	80	0.000	0.007
1q44_gain	15	8	21	83	96	67	0.008	0.110
5p15.33_gain	6	5	16	92	99	72	0.005	0.110
7p22.1_gain	20	6	12	78	98	76	0.007	0.110
8q24.21_gain	20	7	14	78	97	74	0.013	0.141
1p12_gain	10	4	12	88	100	76	0.041	0.277
1q21.3_gain	15	7	16	83	97	72	0.040	0.277
8q11.21_gain	13	5	12	85	99	76	0.054	0.277
11q13.3_gain	2	5	9	96	99	79	0.050	0.277
20q13.33_gain	16	6	9	82	98	79	0.051	0.277
22q13.2_gain	11	5	13	87	99	75	0.056	0.277
6p24.3_gain	19	10	16	79	94	72	0.103	0.419
11q13.4_gain	2	5	8	96	99	80	0.100	0.419
17q25.3_gain	2	6	8	96	98	80	0.109	0.419
4q12_gain	3	2	7	95	102	81	0.122	0.439
12q15_gain	4	3	7	94	101	81	0.276	0.931
5q35.3_gain	1	2	4	97	102	84	0.304	0.966
3p13_gain	4	8	8	94	96	80	0.380	1.000
6q12_gain	4	2	5	94	102	83	0.372	1.000
12q14.1_gain	4	3	5	94	101	83	0.662	1.000
13q12.3_gain	5	2	5	93	102	83	0.347	1.000
15q26.2_gain	3	2	4	95	102	84	0.581	1.000
19p13.2_gain	5	2	2	93	102	86	0.438	1.000
1p36.31_loss	0	0	0	98	104	88	1.000	1.000
1p22.1_loss	0	0	0	98	104	88	1.000	1.000
2q37.3_loss	0	0	0	98	104	88	1.000	1.000
3p24.3_loss	0	0	0	98	104	88	1.000	1.000
3q23_loss	0	0	0	98	104	88	1.000	1.000
4q34.3_loss	0	0	0	98	104	88	1.000	1.000
5p15.31_loss	0	0	0	98	104	88	1.000	1.000
5q11.2_loss	0	0	0	98	104	88	1.000	1.000
5q31.3_loss	0	0	0	98	104	88	1.000	1.000
6q22.31_loss	0	0	0	98	104	88	1.000	1.000
6q26_loss	0	0	0	98	104	88	1.000	1.000
8p23.3_loss	0	0	0	98	104	88	1.000	1.000
9p23_loss	0	0	0	98	104	88	1.000	1.000
9p21.3_loss	0	0	0	98	104	88	1.000	1.000

10p15.3_loss	0	0	0	98	104	88	1.000	1.000
10q23.31_loss	0	0	0	98	104	88	1.000	1.000
10q26.3_loss	0	0	0	98	104	88	1.000	1.000
11p11.2_loss	0	0	0	98	104	88	1.000	1.000
11q23.3_loss	0	0	0	98	104	88	1.000	1.000
12q23.3_loss	0	0	0	98	104	88	1.000	1.000
13q12.11_loss	0	0	0	98	104	88	1.000	1.000
13q34_loss	0	0	0	98	104	88	1.000	1.000
14q23.3_loss	0	0	0	98	104	88	1.000	1.000
14q32.2_loss	0	0	0	98	104	88	1.000	1.000
15q13.3_loss	0	0	0	98	104	88	1.000	1.000
15q14_loss	0	0	0	98	104	88	1.000	1.000
15q15.2_loss	0	0	0	98	104	88	1.000	1.000
16p13.3_loss	0	0	0	98	104	88	1.000	1.000
16q12.1_loss	0	0	0	98	104	88	1.000	1.000
16q24.3_loss	0	0	0	98	104	88	1.000	1.000
19p13.3_loss	0	0	0	98	104	88	1.000	1.000

Supplementary Note 2 – Explanation of Self-Organizing Maps (SOMs) and mosaic plots

Self-organizing maps (SOM) were initially used for gene expression data analysis, however several recent studies applied SOMs also on regulatory data[17–19]. The main advantage of SOM analysis (to identify co-expressed/neighbouring genes), when combined with mosaic plots (to visualize all genes simultaneously), is the reduction of a huge number of variables into a specific number of centroids (represented by "tiles") based on their similarities across the whole dataset. Each tile represents a centroid value of very similar/co-clustered features (the features can be genes or genomic regions). In addition, the SOM analysis does not provide only clustering of the similar features into one tile but also allows clustering of the tiles themselves, which means that neighboring tiles include similarly behaving features. Finally, SOMs can be used for classification of samples not only visually but one can also use the centroid values of all the tiles for hierarchical clustering.

To generate self-organizing maps and mosaic plots from our gene expression and regulatory data, we used a software **GEDI**[20,21] (Gene Expression Dynamics Inspector) v2.1 with default setting of its static analysis (maps of 25x26 tiles per samples, Euclidean distance as a similarity metrics). The manual and the didactic flow diagram of a GEDI tool can be found here:
https://apps.childrenshospital.org/clinical/research/ingber/GEDI/GEDI_v2_1_Manual.pdf
https://apps.childrenshospital.org/clinical/research/ingber/GEDI/GEDI_sumProc.pdf

1. Mosaic plots from RNA-seq data

We used a matrix (across the 11 melanoma cell cultures) of normalized RNA-seq counts that were log2 transformed and median-gene normalized. Only genes having SD>=1 across the cohort of 11 samples were selected, which yielded in 4169 genes. GEDI assigned all the genes into one of 650 tiles (a map of 25x26 tiles) according to the similar behavior across all the samples. In our analysis, the tiles include from 0 to 52 genes. A gene density map visualizes the distribution of genes across the tiles (Figure 1). The SOMs for our cohort of 11 melanoma cell cultures generated by GEDI tool are shown in Figure 2. The red color represents up-regulated centroids/tiles, the blue represent down-regulated centroids/tiles. Because each position (a tile) in the map includes exactly the same genes across all the samples, it is very easy to see differences between samples, for instance genes represented by tiles on the left side are mainly up-regulated only in the first two samples (invasive samples, MM047 and MM099), while other samples (proliferative) have the same tiles mostly down-regulated (blue). Also the high similarity between invasive samples and variability within proliferative samples is evident.

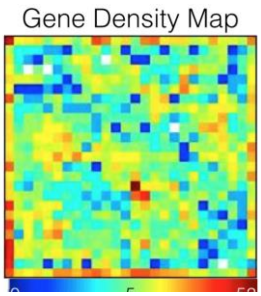

Figure 2. Gene density map generated by GEDI

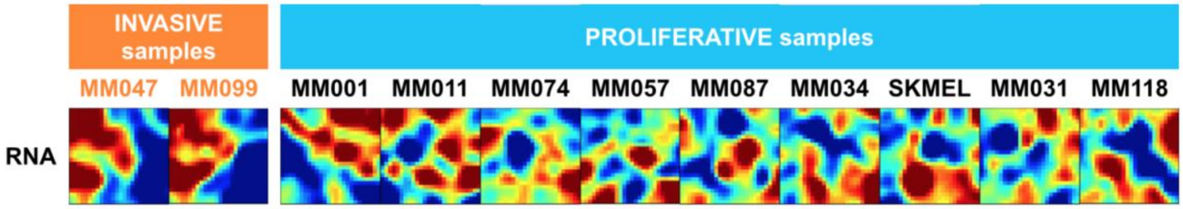

Figure 1. Self-organizing maps for RNA-seq data generated by GEDI.

Unsupervised hierarchical clustering based on centroid's values of all the tiles (650 tiles per sample) furthermore illustrates a clear separation of the invasive samples from the proliferative (Figure 3). The dendrogram also illustrates more heterogeneity between the samples in the proliferative group.

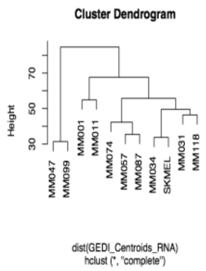

Figure 3. *Hierarchical clustering based on centroid values of gene expression*

An example of a cluster of tiles that are up-regulated in a proliferative sample (MM001) and down-regulated in an invasive sample (MM047) is shown in Figure 4. This cluster consists of 35 tiles that are marked in GEDI maps (Figure 4a). These tiles include expression information for 185 co-expressed genes. A detail of gene expression of 10 genes that were assigned to a tile marked by a diamond in the GEDI plots (including SOX10 and MITF) is represented by heatmaps (Figure 4b) across all 11 samples (both log2 values and median-gene centered are shown, where the first two samples are invasive, the rest proliferative). The expression signal of all 185 gene included in the marked cluster of 35 tiles across 11 samples is shown in Figure 4c.

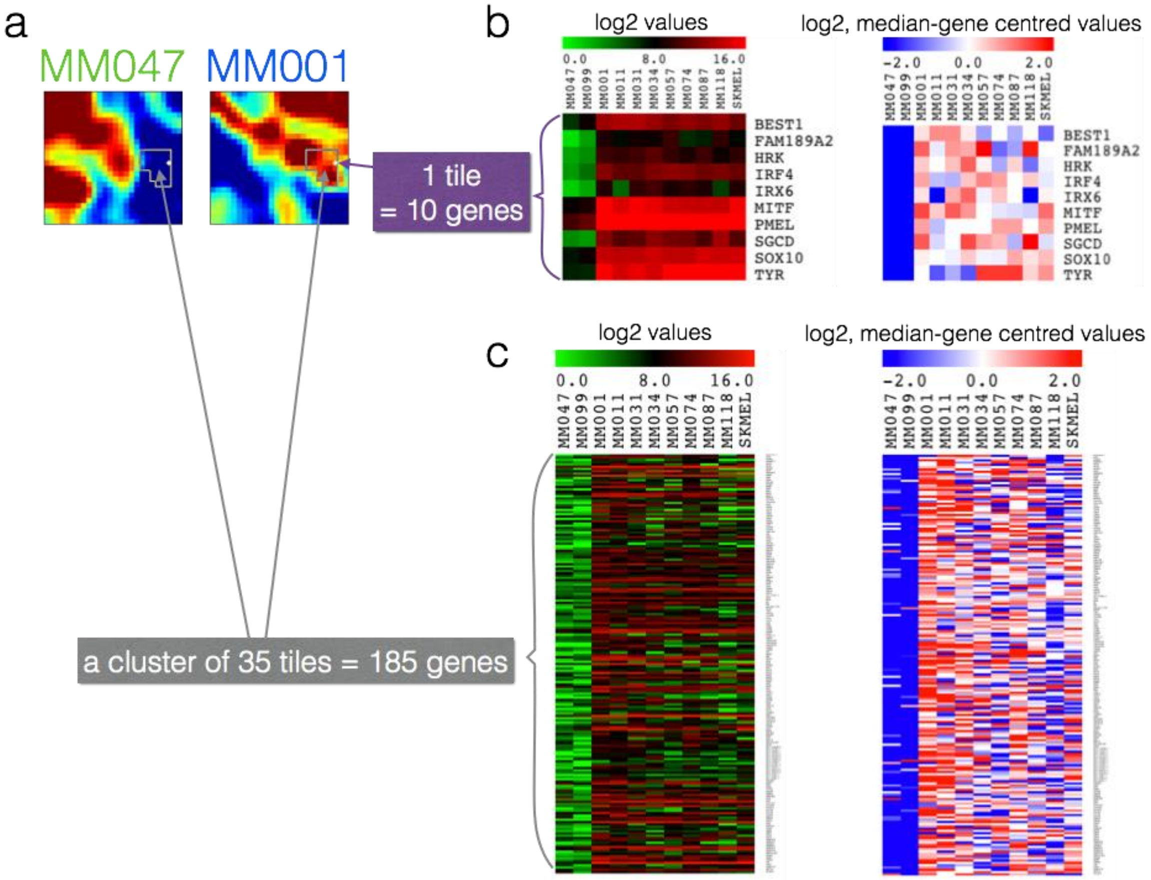

Figure 4. Genes down-regulated in an invasive sample (MM047) versus a proliferative sample (MM001).

An opposite example of a cluster of tiles with genes up-regulated in an invasive sample (MM047) and down-regulated in a proliferative sample (MM001) is shown in Figure 5. In this case a cluster of 26 tiles (Figure 5a) includes 184 genes. Expression of genes in one tile (marked by diamond, including SOX9 gene) across all 11 samples is shown in Figure 5b and the expression of all the genes in the cluster is represented in Figure 5c. Again the first two samples are invasive, the rest are proliferative.

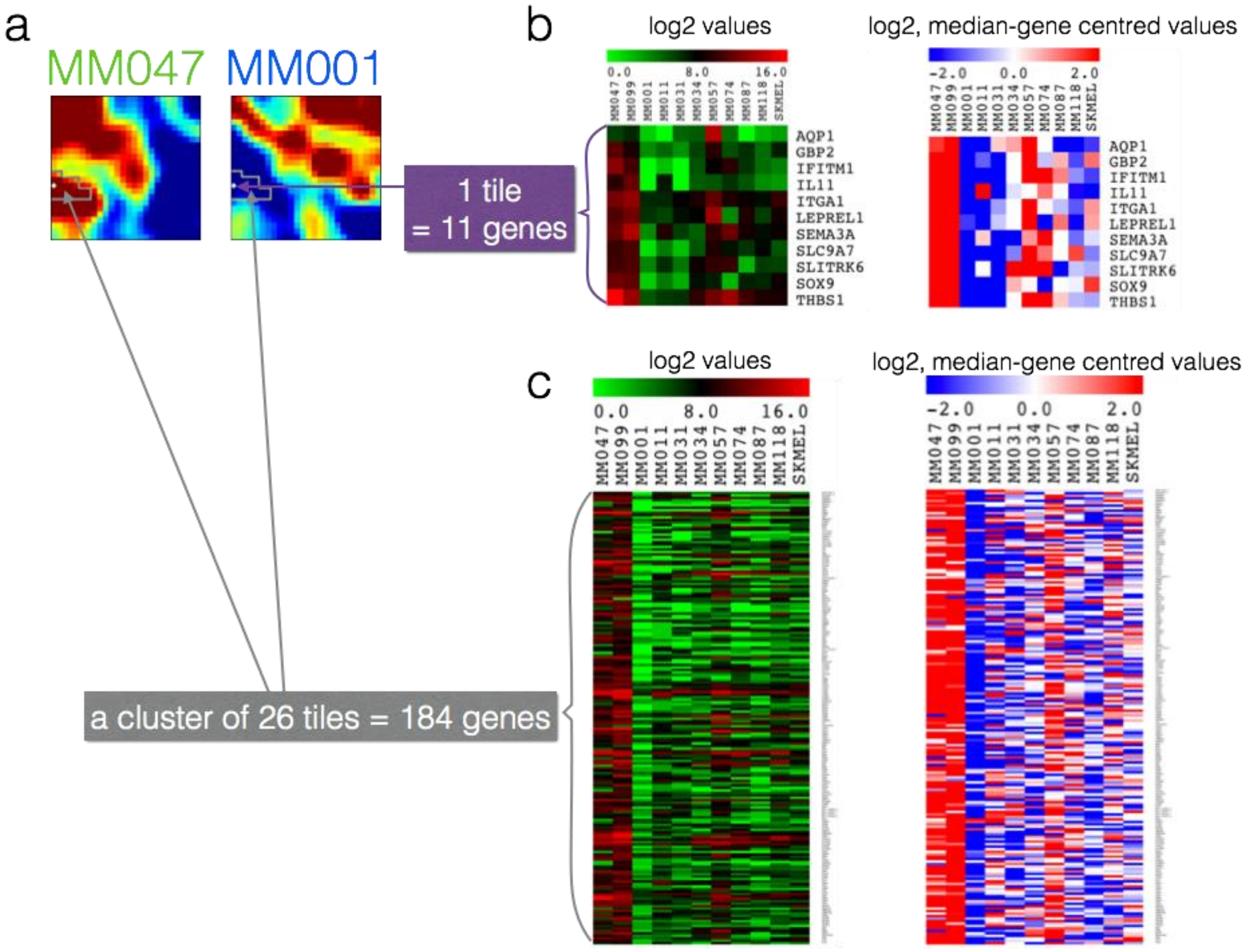

Figure 5. **Genes up-regulated in an invasive sample (MM047) versus a proliferative sample (MM001).**

2. Mosaic plots for ChIP-seq and FAIRE-seq data

The main aim of our study was to investigate the cohort of 11 melanoma cell cultures at the regulatory level. We generated H3K27ac ChIP-seq, H3K27me3 ChIP-seq and FAIRE-seq data for each sample. We then applied self-organizing maps to cluster genomic regions and used mosaic plots to visualize differences within subgroups in the cohort.

To have comparable regions between the samples, we used candidate regulatory regions that we defined a priori using publicly available regulatory datasets: DHS from ENCODE[13], General Binding Preference models[9], CpG islands, proximal promoters, conserved non-coding sequences, ultra-conserved elements, regulatory elements from OregAnno[10], VistaEnhancers[11] and predicted *cis*-regulatory modules[12]. The complete procedure of creating candidate regulatory regions yielded 1,223,024 regions (representing ~35% of the human genome) with average size 818 bp. These regions were used to aggregate the sequence reads in 11 samples (H3K27ac, H3K27me3, FAIRE) and normalized.

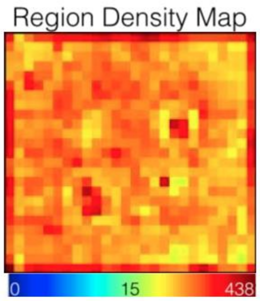

Figure 6. Region density map

The regulatory regions having SD>=1 for H3K27ac and any signal for both H3K27me3 and FAIRE after data normalization were selected (the regions with H3K27m3 and FAIRE do not necessarily have SD>1). This yielded a set of 55919 regulatory regions with signal across 3 different regulatory data layers. The values were region-median centered within each regulatory dataset separately, then scaled across the datasets. The final dataset was used for a static analysis in GEDI with default settings on 26x25 tiles. The number of regions included in each tile varies from 26 to 438 for this specific case; this is shown as a density map representing the distribution of the regions across the tiles (Figure 6). In the mosaic plots (Figure 7), each tile is represented by a centroid value of the signal across all included regions in that tile. The specific position in one map represent a centroid value of the signal across all the samples and across the three different regulatory data. This allows comparing samples between each other as well as between different data levels. For instance, there is a very high similarity between H3K27ac and FAIRE signal, which is complemented with H3K27me3 signal, especially for invasive samples. Again, we observe that invasive samples are clearly separated from the rest, with high similarity between each other, while proliferative samples show higher heterogeneity.

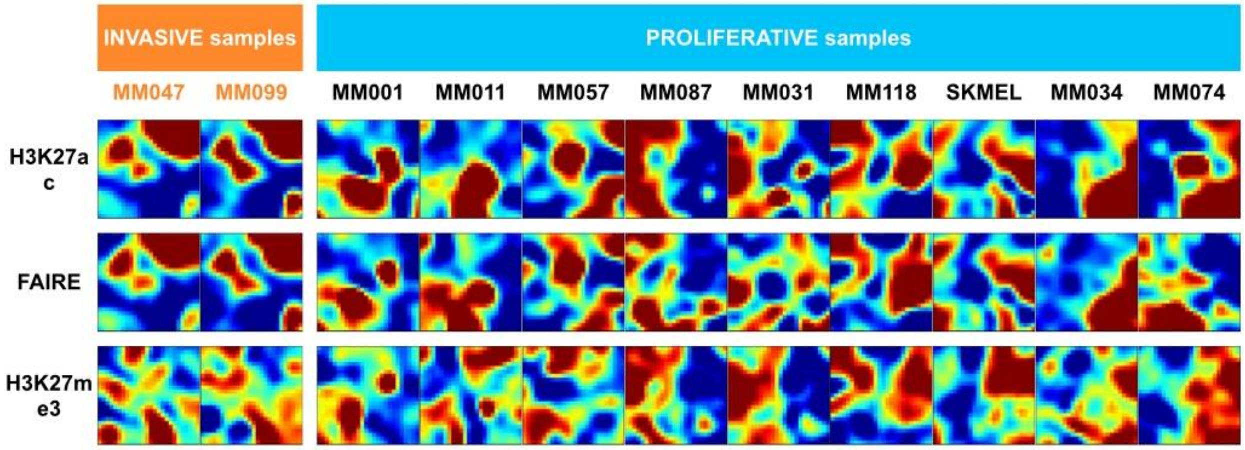

Figure 7. Self-organizing maps generated by the GEDI tool for different regulatory data across 11 cell cultures

Figure 8 represents an example of a cluster of regions that are more active based on H3K27ac in the sample MM047 (invasive) than in the sample MM001 (proliferative). The cluster is marked in GEDI maps (Figure 8a) and includes 20 tiles representing centroids of 1515 regions in total. H3K27ac signal at these 1515 regions is shown in heatmaps (Figure 8b) representing log2 values of the H3K27ac signal at regulatory regions as well as the median-centered values in all 11 samples, where the first two samples are invasive samples, the rest are proliferative. A detailed view of several example regions from this cluster are shown as UCSC screenshots (Figure 8c) where black regions represent candidate regulatory regions which were scored with H3K27ac signal in all 11 samples, the green track represents H3K27ac ChIP-seq signal in MM047 (invasive) sample, the blue track represents H3K27ac ChIP-Seq in MM001 (proliferative) sample. The highlighted region indicates the regions from the selected cluster. The centroids of all regions from this cluster are represented in Figure 8d, where the first two samples are invasive and the rest are proliferative. Figure 9a and Figure 9b represent the same cluster of 20 tiles as shown in Figure 8, but for FAIRE-seq and H3K27me3 ChIP-seq signal respectively. In both cases the signal is not so strong as in Figure 8, especially H3K27me3 is more noisy,

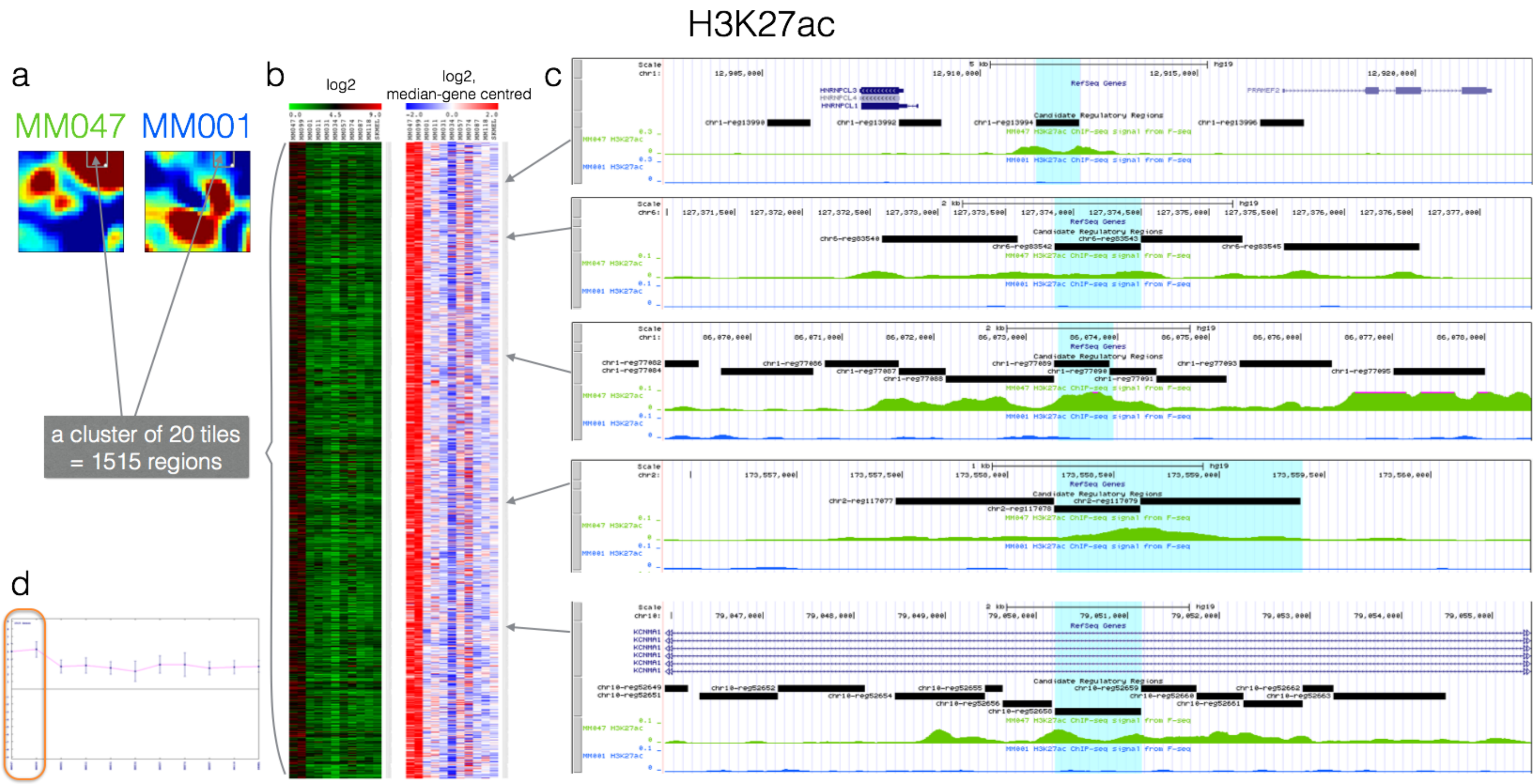

Figure 8. Example of cluster of tiles, which includes regions more active in invasive samples

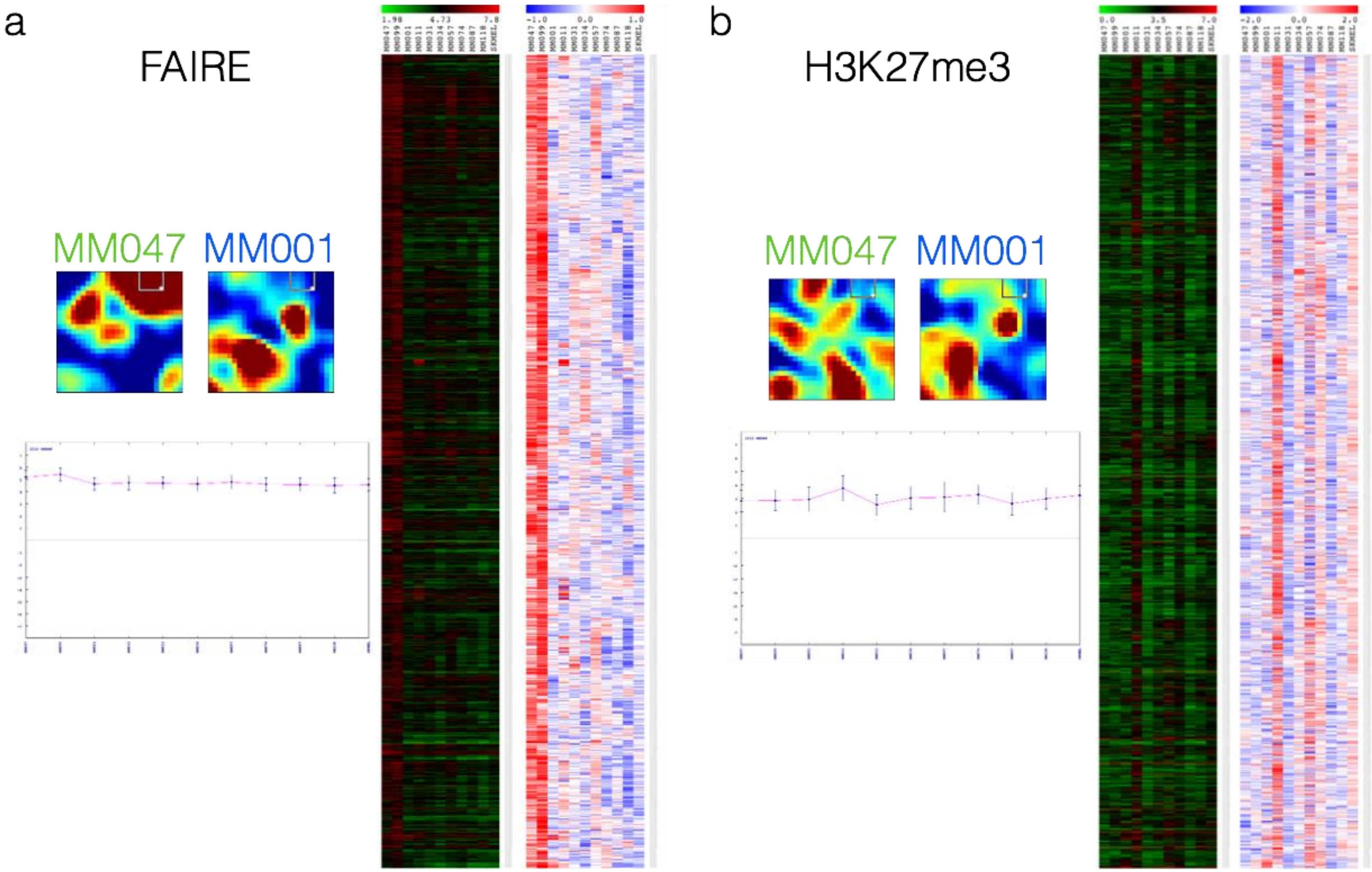

Figure 9. Example of the cluster of regions as in the Fig.8 (20 tiles including 1515 regulatory regions) with signal of FAIRE (a) and H3K27me3 (b)

Finally, Figure 10 represents an example of cluster of regions that are more active in sample MM001 (proliferative) than in sample MM047 (invasive). This cluster is marked in GEDI maps (Figure 10a) and includes 20 tiles representing centroids of 1342 regions in total. All 1342 regions are represented by heatmaps (Figure 10b) with log2 values of H3K27ac signal at regulatory regions as well as median-centered values, where the first two samples are invasive samples, the rest are proliferative. Detailed examples of several of these regions are shown as UCSC screenshots (Figure 10c) where black regions represent candidate regulatory regions that were scored with regulatory signal (H3K27ac) for all 11 samples. The green track represents H3K27ac ChIP-seq signal in MM047 (invasive) sample, the blue track represents H3K27ac ChIP-Seq in MM001 (proliferative) sample, and the highlighted region indicates the regions from the selected cluster. The centroids of all regions from this cluster are represented in Figure 10d, where the first two samples are invasive and the rest are proliferative. Figure 11a and Figure 11b represent the same cluster of 20 tiles as shown in Figure 10, but for FAIRE-seq and H3K27me3 ChIP-seq signal respectively.

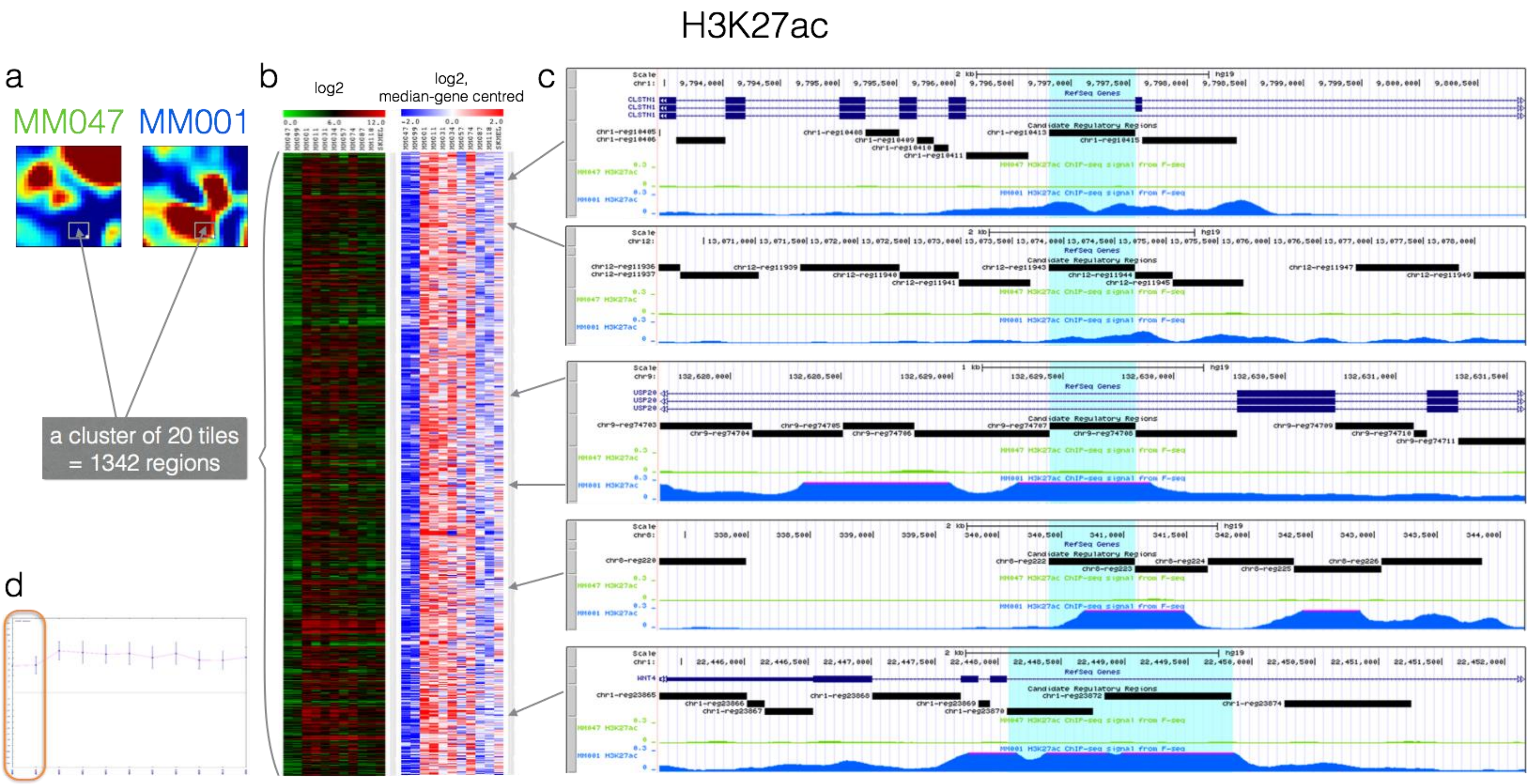

Figure 10. Example of cluster of tiles which includes regions more active in proliferative samples

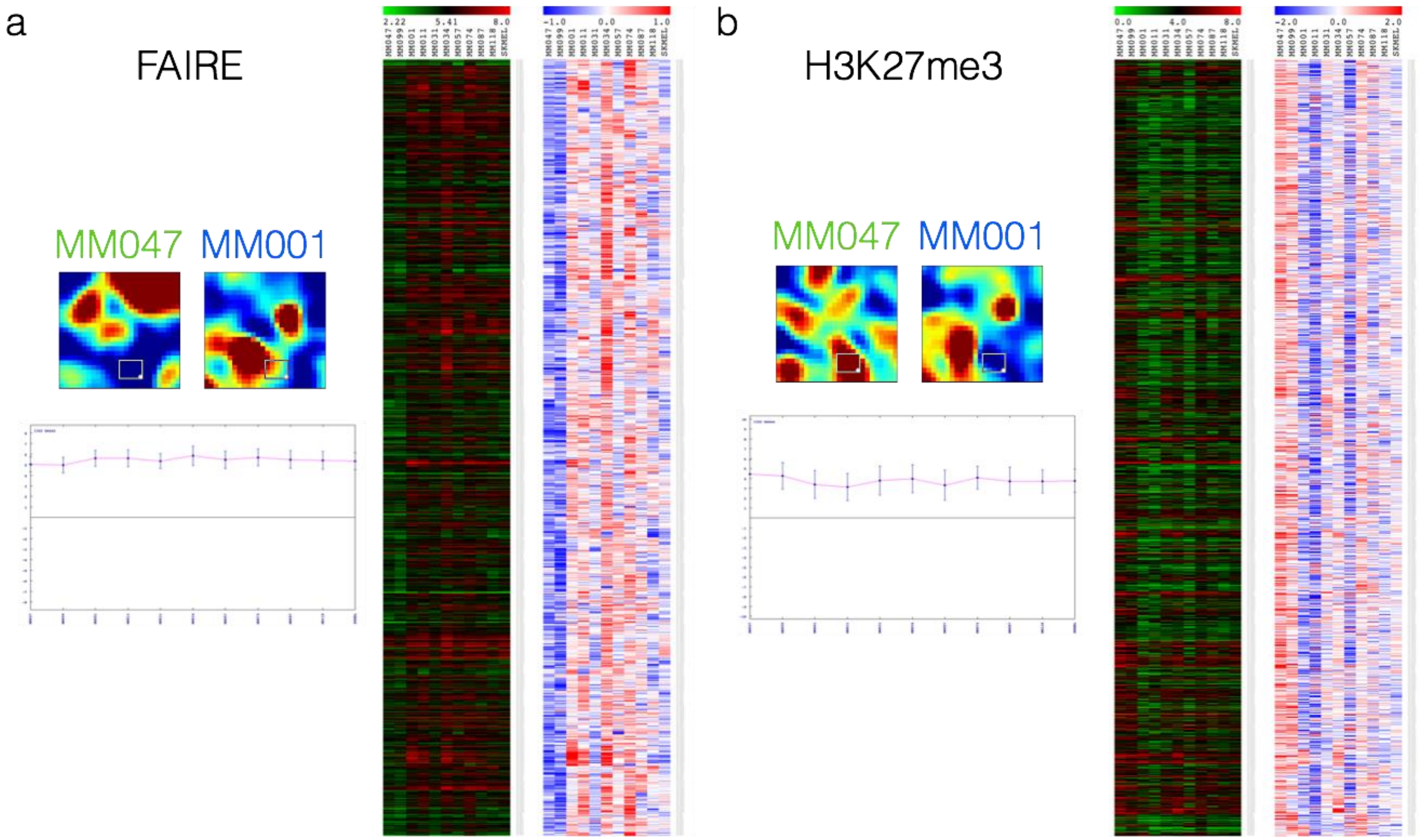

Figure 11. Example of the same cluster of regions as in the Fig.10 (20 tiles including 1342 regulatory regions) with signal of FAIRE (a) and H3K27me3 (b)

Supplementary References:

1. Hoek, K. S. *et al.* In vivo Switching of Human Melanoma Cells between Proliferative and Invasive States. *Cancer Res.* **68,** 650–656 (2008).
2. Cheng, W.-Y., Yang, T.-H. O. & Anastassiou, D. Biomolecular Events in Cancer Revealed by Attractor Metagenes. *PLoS Comput Biol* **9,** e1002920 (2013).
3. Strub, T. *et al.* Essential role of microphthalmia transcription factor for DNA replication, mitosis and genomic stability in melanoma. *Oncogene* **30,** 2319–2332 (2011).
4. Herrmann, C., Sande, B. V. de, Potier, D. & Aerts, S. i-cisTarget: an integrative genomics method for the prediction of regulatory features and cis-regulatory modules. *Nucleic Acids Res.* **40,** e114–e114 (2012).
5. Janky, R. *et al.* iRegulon: From a Gene List to a Gene Regulatory Network Using Large Motif and Track Collections. *PLoS Comput. Biol.* **10,** e1003731 (2014).
6. Hoek, K. S. *et al.* Novel MITF targets identified using a two-step DNA microarray strategy. *Pigment Cell Melanoma Res.* **21,** 665–676 (2008).
7. Shakhova, O. *et al.* Sox10 promotes the formation and maintenance of giant congenital naevi and melanoma. *Nat. Cell Biol.* **14,** 882–890 (2012).
8. Andersson, R. *et al.* An atlas of active enhancers across human cell types and tissues. *Nature* **507,** 455–461 (2014).
9. Ernst, J., Plasterer, H. L., Simon, I. & Bar-Joseph, Z. Integrating multiple evidence sources to predict transcription factor binding in the human genome. *Genome Res.* **20,** 526–536 (2010).
10. Montgomery, S. B. *et al.* ORegAnno: an open access database and curation system for literature-derived promoters, transcription factor binding sites and regulatory variation. *Bioinforma. Oxf. Engl.* **22,** 637–640 (2006).
11. Pennacchio, L. A. *et al.* In vivo enhancer analysis of human conserved non-coding sequences. *Nature* **444,** 499–502 (2006).
12. Ferretti, V. *et al.* PReMod: a database of genome-wide mammalian cis-regulatory module predictions. *Nucleic Acids Res.* **35,** D122–D126 (2007).
13. ENCODE Project Consortium. An integrated encyclopedia of DNA elements in the human genome. *Nature* **489,** 57–74 (2012).
14. Fisher, R. A. On the Interpretation of $\chi 2$ from Contingency Tables, and the Calculation of P. *J. R. Stat. Soc.* **85,** 87–94 (1922).
15. Benjamini, Y. & Hochberg, Y. Controlling the False Discovery Rate: A Practical and Powerful Approach to Multiple Testing. *J. R. Stat. Soc. Ser. B Methodol.* **57,** 289–300 (1995).
16. Shi, H. *et al.* Melanoma whole-exome sequencing identifies (V600E)B-RAF amplification-mediated acquired B-RAF inhibitor resistance. *Nat. Commun.* **3,** 724 (2012).
17. Mortazavi, A. *et al.* Integrating and mining the chromatin landscape of cell-type specificity using self-organizing maps. *Genome Res.* **23,** 2136–2148 (2013).
18. Heidari, N. *et al.* Genome-wide map of regulatory interactions in the human genome. *Genome Res.* **24,** 1905–1917 (2014).
19. Boyle, A. P. *et al.* Comparative analysis of regulatory information and circuits across distant species. *Nature* **512,** 453–456 (2014).
20. Eichler, G. S., Huang, S. & Ingber, D. E. Gene Expression Dynamics Inspector (GEDI): for integrative analysis of expression profiles. *Bioinformatics* **19,** 2321–2322 (2003).
21. Guo, Y., Eichler, G. S., Feng, Y., Ingber, D. E. & Huang, S. Towards a Holistic, Yet Gene-Centered Analysis of Gene Expression Profiles: A Case Study of Human Lung Cancers. *BioMed Res. Int.* **2006,** e69141 (2006).
